# A community-engaged approach to developing common data elements: a case study from the RADx-UP Long COVID common data elements Task Force

**DOI:** 10.1093/jamiaopen/ooaf046

**Published:** 2025-06-04

**Authors:** Helena L Pike Welch, Gregory Guest, Halima Garba, Gabriel A Carrillo, Allyn M Damman, Warren A Kibbe

**Affiliations:** Duke Clinical Research Institute, Duke University School of Medicine, Durham, NC 27701, United States; Center for Health Equity Research, University of North Carolina, Chapel Hill, NC 27516, United States; Duke Clinical Research Institute, Duke University School of Medicine, Durham, NC 27701, United States; Duke Clinical Research Institute, Duke University School of Medicine, Durham, NC 27701, United States; Department of Pediatrics, Duke University School of Medicine, Durham, NC 27701, United States; Duke Clinical Research Institute, Duke University School of Medicine, Durham, NC 27701, United States; Department of Biostatistics and Bioinformatics, Duke University School of Medicine, Durham, NC 27701, United States; Duke Cancer Institute, Duke University School of Medicine, Durham, NC 27701, United States

**Keywords:** community engagement, common data elements, community-informed research, community partners, Long COVID

## Abstract

**Objectives:**

In response to requests from several Rapid Acceleration of Diagnostics-Underserved Populations (RADx-UP) community-engaged research projects to include Long COVID common data elements (CDEs) in the existing RADx-UP CDEs, the RADx-UP Coordination and Data Collection Center (CDCC) leadership formed the Long COVID CDEs Task Force.

**Materials and Methods:**

The Task Force, composed mainly of community partners and RADx-UP project members, participated in various activities to evaluate the Long COVID CDEs fit for purpose from the Researching COVID to Enhance Recovery (RECOVER) program for RADx-UP use.

**Results and Discussion:**

The Task Force’s efforts led to a compilation of lessons learned and the creation of a novel set of 28 CDEs that are appropriate for community-engaged research in Long COVID.

**Conclusion:**

Utilization of standardized CDEs does not always work for the communities involved in the research, but creation of a community-involved task force can lead to a meaningful, rich set of CDEs.

## Background and significance

The standardization and exchange of quantitative data are becoming increasingly common in clinical research.^1^^,^^2^ By standardizing data, researchers can quickly compare and combine datasets from multiple sites, allowing for more complex analysis powered by more robust samples. Standardization not only aids in data sharing but also enhances the insights gained from translational studies.^3^ To achieve this, researchers use “common data elements” (CDEs),^2^^,^^4^ which are consistent variables (question stems and response options) common across multiple datasets.^5^ CDEs help streamline the standardization, collection, harmonization, and linkage of data among various studies.^6^

Traditionally, CDEs are often crafted by subject matter experts,^7^ with little or no input from the data-contributing communities they serve. However, this researcher-directed approach has been questioned, with researchers noting its potential risk of bias and failure to accurately reflect community experiences.^8–10^ Calls have increased among the scientific community for greater community engagement in the development and implementation of these research tools,^11^^,^^12^ with the aim of creating more valid data constructs better representing the lived experiences of the studied populations. Despite this, methodology is currently lacking for engaging communities in the CDE development process.

The National Institutes of Health (NIH) has incorporated CDEs into clinical research for over two decades, yet the creation and application of community-informed CDEs, especially during rapidly moving health crises like the COVID-19 pandemic, remain poorly researched.^13^ One notable exception, the NIH-funded Rapid Acceleration of Diagnostics-Underserved Populations (RADx-UP) program, represents the most substantial commitment to health disparities research ever made by the NIH.^14^ As of February 2024, the consortium of community-engaged research projects, with the goal of increasing access to COVID-19 testing in underserved populations, has funded more than 138 projects nationally.^15^

Nevertheless, while RADx-UP’s community-based approach to selecting and refining CDEs deployed in underserved populations was a commendable step toward inclusivity, the exigencies of the COVID-19 health crisis necessitated a faster, less-than-ideal participatory process.^16^ The process used to select and adapt these CDEs^17^ entailed working with community partners to transform an initial list of more than 700 CDEs from the NIH CDE repository, the NIH Disaster Response Resources, and the PHENotypes and eXposures (PhenX) Toolkit into a more pragmatic, well-informed CDE architecture for RADx-UP. The work by Carrillo et al^17^ generated useful and meaningful CDEs deployed in a variety of settings, but the process revealed several challenges, including the perception of being rushed forward with inadequate consideration of community concerns. Additional, notable issues raised by projects were the potential to worsen stigma with culturally insensitive CDEs, along with data privacy and CDE relevance. The authors’ report on the effort concluded by highlighting the need to foster mutual trust and respect, with the importance of “clear messaging, stating intent, assessing local factors, and providing more direct communications with stakeholders in the community.”^17^ (p. 1485).

Building on these insights in the spring of 2022, the RADx-UP consortium was tasked with creating an additional set of participant-facing CDEs to better understand post-acute sequelae of SARS-CoV-2, commonly known as “Long COVID.” Existing CDEs were considered for use, but since they were mainly traditional CDEs intended for electronic health record and longitudinal observational cohort studies,^18^ it was agreed that a novel set of participant-facing CDEs should be created.

Drawing from the experience gained and lessons learned from generating the first set of CDEs, we initiated a process to create community-engaged CDEs to address issues of relevance, cultural sensitivity, data privacy, and rushed timelines.^17^ Utilizing a participatory approach, we incorporated feedback to enhance readability, create translatable answer choices, and reduce redundancy, thereby mitigating relevance and cultural sensitivity challenges. This approach also alleviated participant burden which may reduce data privacy concerns. To ensure thorough consideration of community-engaged feedback, our process was 6 months (initially), compared to Carrillo et al’s 3-month community-engaged process.^17^

In this article, the topic of which was developed during a meeting between the authors to reflect on the CDE creation process after completion of all activities, we describe the (revised) process used and how it compared to the first experience. We also highlight additional lessons learned and limitations that we hope will help inform future national research initiatives.

## Materials and methods

### Review of RECOVER CDEs

**Figure 1.**
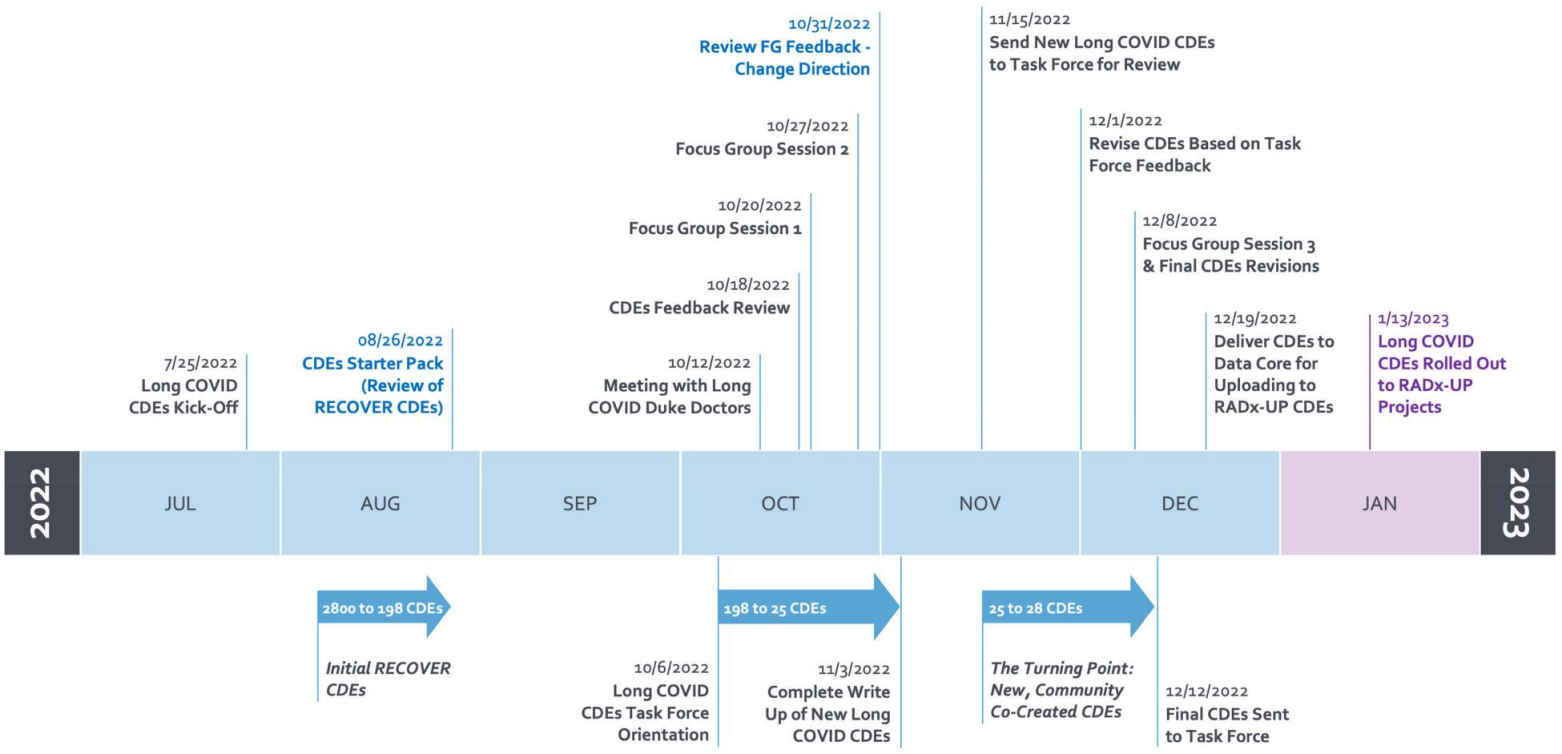
Long COVID CDEs Task Force timeline.

The RADx-UP Coordination and Data Collection Center (CDCC) program leadership formed a Long COVID CDEs Task Force charged with drawing on the lessons learned from Carrillo et al^17^ to collaborate with the RADx-UP project teams and community partners in adding Long COVID CDEs to the tier 2 (optional) RADx-UP CDEs. The Task Force Planning Team comprised faculty and staff members of the CDCC. Figure 1 illustrates the Task Force timeline from inception to end, with arrows showing the evolution of the CDEs. In August 2022, the Planning Team began developing the components and structure of the Task Force and focus group sessions.

In addition to members of the RADx-UP CDCC, the Task Force would also include roughly 25 members of the RADx-UP projects, including project staff, principal investigators, community partners, and Long COVID patients. The RADx-UP community partners lived and worked in the communities that the projects were intended to reach. Community partners collaborated with principal investigators and project staff to assist with the study from start-up to close-out. To prepare for the inaugural meeting with the Task Force, the Planning Team was tasked with identifying an initial set of existing Long COVID CDEs. After reviewing existing sources, the Planning Team selected the Long COVID CDEs developed by the NIH Researching COVID to Enhance Recovery (RECOVER) initiative, aimed at studying symptoms and finding therapies to treat Long COVID.^18^^,^^19^

Before presenting to the Task Force, the Planning Team reviewed the approximately 2700 RECOVER CDEs to build a starter pack of 198 Long COVID CDEs, which were grouped into 20 categories, and then developed a feedback survey for the Task Force to review the CDEs.

The initial Task Force meeting design was to hold an orientation session and two focus group sessions, with all sessions conducted via Zoom. A request for volunteers to join the Task Force was sent in mid-September 2022 to all RADx-UP projects and community partners. Thirty-seven respondents indicated interest in joining the Task Force, with 24 ultimately agreeing to commit to the work and time requirements of the Task Force. The Task Force comprised 15 community partners and Long COVID patients, 6 principal investigators including physicians, and 3 project staff members. The 15 community partners represented a diverse background of communities: Hispanic/Latinx, Black/African American, Asian American, Pacific Islander, Native Hawaiian, and American Indian/Alaska Native. They also worked with children, pregnant women, and/or older adults in both rural and urban settings. Three of the Task Force members were Long COVID patients.

An orientation session in early October 2022 introduced the purpose, timeline, and participation activities for the Task Force. The session also presented the Long COVID CDEs starter pack (see Supplementary CDEs S1 online) with an explanation of the feedback survey each member would complete in their review of the starter pack (see Supplementary Survey S1 online). Finally, the dates and structure of the upcoming focus groups sessions were presented as next steps. Of the 24 individuals invited to join the Task Force, 17 either attended the orientation session or watched the recording of the session, as required for active participation in the Task Force.

Ahead of the focus group sessions, the Planning Team members met with two Duke Hospital physicians who were treating Long COVID patients to gain their perspectives on what was being seen in the field relevant to the condition and to ascertain the most common complaints, symptoms, and affected body systems they were seeing with their patients. In tandem, all feedback surveys were compiled to gain an initial assessment of the utility of the CDEs starter pack for the RADx-UP community. Task Force participants were given the option to attend one or both focus group sessions held in October 2022, with 11 of 17 attending the first focus group session and 10 of 17 attending the second focus group session.

### Design of novel Long COVID CDEs

Feedback from the Task Force, following both the CDEs starter pack feedback survey and the first focus group session, made it clear that the RECOVER CDEs were marked by many of the drawbacks outlined in Carrillo et al,^17^ including the use of medical jargon, redundant and verbose questions, and a lack of examples and definitions, notably no definition for Long COVID. Table 1 illustrates the range of problems with the RECOVER CDEs with examples identified by the Task Force for each of the problems identified by Carrillo et al.^17^

**Table 1.** Challenges of RECOVER CDEs as identified by the Task Force with examples.

Challenges	Examples from RECOVER CDEs
Need to use plain language: avoid medical jargon and improve readability for those with low literacy levels.	Please tell us at what time(s) you have had any of the following symptoms. Post-exertional malaise (Symptoms worse after even minor physical or mental effort) Palpitations, racing heart, arrhythmia, skipped beats
Provide clarity using definitions and examples. (In this example, the Task Force requested clarification on whether the question pertains solely to teeth or includes all oral issues and asked for examples of specific dental problems to make the question clearer.)	Please tell us at what time(s) you have had any of the following symptoms. Check all that apply. Problems with teeth
Comparison to baseline (pre-COVID-19 infection) to distinguish Long COVID symptoms.	In the past year, have you ever lost control of your bladder function?
Burdensome survey length for both questions and answers—198 questions with multiple, confusing answer choices. (The example illustrates the confusing answer choices that the Task Force noted; also does not translate well into languages other than English, thus increasing the survey burden.)	Are you able to go for a walk of at least 15 minutes? 5, Without any difficulty 4, With a little difficulty 3, With some difficulty 2, With much difficulty 1, Unable to do
Redundancy of questions: some felt similar and condensing could reduce length.	PTSD Section: Trouble falling or staying asleep? Depression Section: Trouble falling or staying asleep, or sleeping too much:
Confusion around timespan and length of time of symptoms.	In the YEAR BEFORE your COVID infection on [DATE], where were you having pain? AROUND your COVID infection on [DATE], where were you having pain? BETWEEN 30 DAYS AFTER your COVID infection on [DATE] AND NOW, where were you having pain?
Consistency of answer choices.	For the symptom of dry mouth that you had had for the longest period of time, is this symptom: 1, I have not had any of these symptoms 2, Getting much worse 3, Getting somewhat worse 4, Staying about the same 5, Getting somewhat better 6, Getting much better 7, Completely gone …indicate how much limitation you have had due to chest pain, chest tightness, or angina over the past 4 weeks. Walking indoors on level ground 1, Extremely limited 2, Quite a bit limited 3, Moderately limited 4, Slightly limited 5, Not at all limited 6, Limited for other reasons or did not do the activity

The Planning Team devised a strategy to pivot away from the starter pack to start fresh by designing a novel set of CDEs unique to RADx-UP, and during focus group session 2 proposed the creation of this novel set of Long COVID CDEs to ensure the entire Task Force was onboard.

Once the Planning Team developed the set of novel Long COVID CDEs, the Task Force reviewed and provided feedback on the CDEs (see Supplementary Survey S2 online). Six Task Force members responded to the survey, all agreeing that the novel Long COVID CDEs accomplished their purpose, the symptom-based questions included the most critical symptoms of Long COVID, the definition accurately described Long COVID, and the Task Force accomplished its purpose to produce Long COVID CDEs appropriate for the RADx-UP community. Of the respondents, 83% agreed the sections were easy to read, 75% rated the novel CDEs as good or excellent for inclusiveness to a wide range of communities.

Several changes were suggested and incorporated to the CDEs, such as adding additional treatment options, like community health centers and staying home, for the highest level of care for any COVID infections, and changing “quality of life” to “everyday life” to be more inclusive of RADx-UP participants. This feedback and suggested changes (see Supplementary Survey Report S1 online) were incorporated into the novel CDEs, which the Planning Team presented to the RADx-UP Leadership Team. The set of novel Long COVID CDEs identified COVID-19 infection treatments, long-term symptoms experienced after the first COVID-19 infection, participants’ lived experience with Long COVID, and impact on quality of life.

The novel CDEs included a definition for Long COVID. To address Task Force feedback concerning the complexity of the definition, it was lowered to a 7.5-grade reading level (see Supplementary Survey Report S1 online). The CDEs included metadata, such as versioning, timestamps, and sources, allowing researchers to track definitions and maintain consistency across studies over time. The proposed definition aligns broadly with clinical descriptions (e.g., CDC/WHO) but intentionally remains flexible to reflect evolving science and patient-centered feedback. Table 2 identifies how feedback changed the novel Long COVID CDEs.

**Table 2.** Utilizing Task Force feedback to create novel Long COVID CDEs with examples.

Novel Long COVID CDE challenges addressed	Novel Long COVID CDE examples
Limited medical jargon and reduced Flesch-Kinkaid grade level to 7.5.	How much has having Long COVID affected your everyday life?
Provided a definition for Long COVID, and for ambiguous terms provided examples.	Since your first COVID-19 infection, have you had new or worsening fatigue (tire easily, decreased energy, etc.)?
Distinguished Long COVID symptoms from baseline by adding “since your first COVID-19 infection…”	Since your first COVID-19 infection, have you had new or worsening problems breathing?
Shortened to 28 questions with clearer answer choices.	Do you know what Long COVID is? (1) Yes (2) No (99) Prefer not to answer
Avoided redundancy by using RADx-UP Tier 1 CDEs related to positive COVID-19 testing as branching logic to open Long COVID CDEs.	RADx-UP Tier 1 CDE: Have you ever tested positive for COVID-19? (1) Yes (2) No (98) Don’t know (99) Prefer not to answer Branching Logic to open Long COVID CDEs: [tested_positive_for_covid] = “1”
Clarified time by specifying the timespan and length (ie, year, month, number of weeks).	Think about the symptoms above. How many weeks did you have these symptoms?
Utilized consistent answer choices, when possible, across sections with words that translate into other languages (eg, “somewhat” and “quite a bit” do not have literal translations in Spanish, whereas “moderately,” “very,” and “slightly” do).	How much has having Long COVID affected your everyday life? (1) Not at all (2) Slightly (3) Moderately (4) Very (5) Extremely (99) Prefer not to answer

The novel Long COVID CDEs differ from the RECOVER CDEs in that there are fewer questions reducing redundancy, the answer choices are more consistent, by not using medical jargon the language is more inclusive and less complex, and there is a definition included for Long COVID. The novel CDEs are intended to improve clarity and usability, especially for participants. It is worth noting that alignment with standardized vocabularies was outside the scope of this community-engaged process. As a result, this may make direct aggregation with older datasets more challenging. However, tools such as mapping tables and conversion rules could address this. Both methods played an important part in aggregating other RADx-UP CDEs with older datasets. The novel CDEs aim to reflect broader applicability and participant-centered feedback, which enhances future standardization.

The final focus group session was held in December 2022, with 9 Task Force members attending, to receive culminating feedback that was incorporated into the initial public release of the 28 novel Long COVID CDEs. Six Task Force members attended all three sessions with an even split between community partners and project team members. The Long COVID CDEs were officially rolled out as part of the RADx-UP tier 2 CDEs version 1.7 on January 13, 2023, for use by projects (see Supplementary CDEs S2 and S3 online). These CDEs, which we anticipate being utilized by community-engaged researchers, are available at https://radx-up.org/research/cdes/ and through the NIH RADx Data Hub (https://radx-hub.nih.gov/).

Following conclusion of the Task Force, a final evaluation survey was sent to all individuals involved in the Task Force process, including Task Force members and the Planning Team (see Supplementary Survey S3 online). Eighteen responses were received, with 4 partially completed responses excluded. Fourteen respondents gave their feedback on the process (see Supplementary Survey Report S2 online). Of these, 79% felt they could offer their expertise, 92% felt their opinions were valued, and 71% agreed the Task Force adequately represented communities impacted by Long COVID. Respondents cited a desire to serve and represent their communities as a driving force for continued participation in the Task Force. However, there were calls for greater inclusion of community members, organizations, and physicians in future community-engaged CDE development focus groups. Suggestions for engaging community members in future focus groups included offering incentives, holding sessions only for community members, increasing outreach and referrals to community partners and leaders, and involving community leaders representing certain minority or indigenous groups.

## Lessons learned

This Task Force experience offers important lessons learned that can guide future programs or consortiums that want to take part in community-engaged, multi-site quantitative research. These lessons were agreed upon and compiled by the authors during the same reflection meeting that also identified the paper topic.

The first lesson learned was that the *involvement of the RADx-UP projects and community partners* was invaluable. If the RADx-UP CDCC had not involved the community members and projects in the CDE process, the same challenges outlined by Carrillo et al may have been repeated,^17^ and the CDCC could have potentially rolled out the RECOVER CDEs as part of the RADx-UP Tier 2 CDEs.

**Figure 2.**
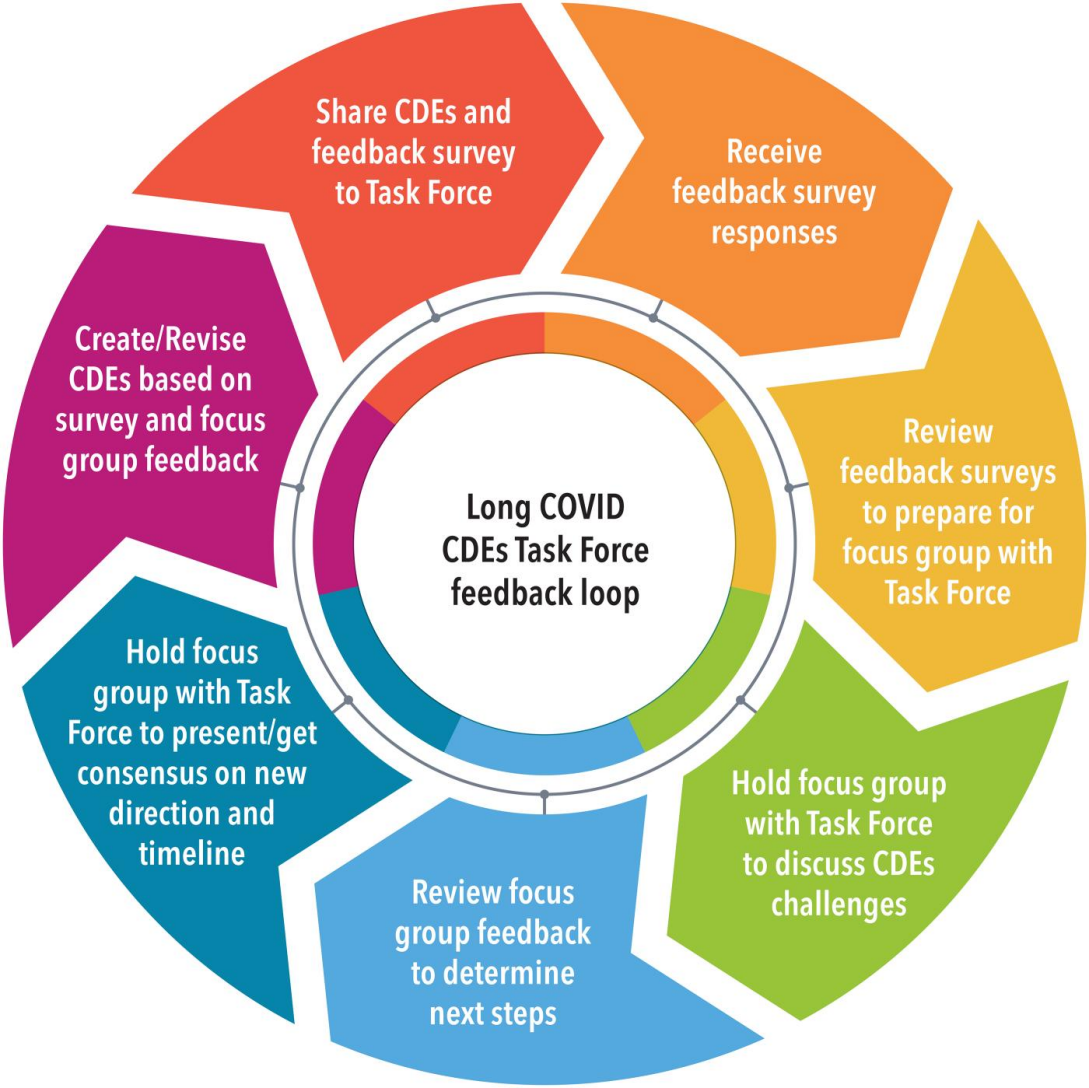
Long COVID CDEs Task Force feedback loop.

Second, involving community partners and projects in *the creation of CDEs for community-engaged research takes extra time, planning, and effort* to result in a rich set of meaningful questions of relevance to the communities being surveyed. In our experience, if community partners are asked to be part of the process, they are ready and willing to participate. In every step of the process, the Planning Team solicited feedback from the community partners and projects so their voices were incorporated into the development of the CDEs, ensuring that they were aligned with researcher and community needs. Figure 2 is a diagrammatic representation of the feedback loop, showing how input from the Task Force was solicited, reviewed, and then applied to the revisions of the CDEs. This process required the Planning Team to listen and be flexible enough to pivot in a new direction from its initial plan.

Third, to take full advantage of researcher and community partner feedback, *be prepared to revisit the plan, deliverables, and timeline*. In our case, the timeline was extended from November 2022 to December 2022 to write, review, and revise the novel Long COVID CDEs, activities not initially planned.

Lastly, as the Task Force and the Planning Team identified a need to give something back to the RADx-UP participants taking the Long COVID survey, the last item in the novel Long COVID CDEs was to develop a list of Long COVID clinics, information, and resources available in the participants’ local region. Giving back to the participants who make research possible is essential to establishing and maintaining relationships, enhancing trust, and showing participants that they are a valued, integral part of research.

## Limitations

In addition to the lessons learned, the Task Force process also presented some limitations. First, neither the Long COVID CDEs or the feedback process (Figure 2) were pilot tested prior to rolling out the CDEs. Although physicians (at Duke as well as Task Force members), biostatisticians, researchers, bioinformaticists, and community partners were consulted and ultimately influenced the CDEs, formal pilot testing could have strengthened the CDEs and ensured both construct and criterion-related validity.

Second, currently there is no plan to evaluate the novel Long COVID CDEs at the conclusion of RADx-UP. Evaluating the CDEs for real-world use could have more accurately measured the impact on the communities being surveyed. Just as pilot testing should be completed on the CDEs prior to rollout, a plan should be in place to evaluate the CDEs for real-world impact and use.

Third, although the process to develop the Long COVID CDEs took 7 months, it was initiated in the middle of the RADx-UP program as a response to a request for Long COVID CDEs to be added to the RADx-UP CDEs. Ideally, the Task Force process to develop community-engaged CDEs should have started at the outset of the program to ensure the process could be in place to allow for the appropriate time to develop, pilot test, evaluate, and roll out new CDEs.

### Informatics and data interoperability considerations

Finally, an essential consideration in the development of these CDEs is their alignment with existing informatics frameworks. While this study prioritized a community-centered approach, future efforts should explore semantic mapping of the newly developed CDEs with established vocabularies, ontologies, and clinical data models to ensure interoperability across research studies. Aligning these CDEs with standardized terminologies such as SNOMED CT or LOINC would enhance their usability in broader informatics applications. Future work may benefit from building on this foundation by mapping community-informed CDEs to existing standards such as SNOMED CT or LOINC to enhance interoperability and downstream usability.

## Conclusion

The formation of the Task Force to develop Long COVID CDEs for the RADx-UP consortium highlights not only the importance of community-engaged clinical research but also the value of community-led contributions. The process followed in developing the Long COVID CDEs reveals that utilizing standardized CDEs does not always meet the needs of the communities engaged in research, especially underserved populations. Taking extra time (in this case, one month) to create a community-involved task force that incorporates the needs and concerns of communities can lead to the creation of richer and more meaningful CDEs. Our implementation of a community-led approach led to a novel set of 28 Long COVID CDEs that aligned with the objectives of the research being done and the communities served by RADx-UP. This can pave the way for future community-engaged clinical researchers to directly incorporate their communities in the CDE development process.

## Supplementary Material

ooaf046_Supplementary_Data

## Data Availability

RADx-UP project data collected using the Long COVID CDE data will be available from the NIH RADx Data Hub. The Long COVID CDEs are available at https://radx-up.org/research/cdes/ and from the RADx Data Hub.
