# Supplementary material for: A community-engaged approach to developing common data elements: a case study from the RADx-UP Long COVID common data elements Task Force
Source: JAMIA Open. 2025 Jun 4;8(3):ooaf046. doi: 10.1093/jamiaopen/ooaf046 (PMC12136053; doi:10.1093/jamiaopen/ooaf046)
Supplement: ooaf046_Supplementary_Data [file ooaf046_supplementary_data.zip › Supplementary CDEs 1_StarterPack(RECOVER)CDEs.pdf]

Data Dictionary Codebook

08/05/2022 1:25pm

	#	Variable / Field Name	Field Label<br>*Field Note*	Field Attributes (Field Type, Validation, Choices, Calculations, etc.)
Instrument: **Pasc Symptoms** (pasc_symptoms)				
	1	[phq_intro]	Over the past two weeks, how often have you been bothered by the following problems:	text<br>Field Annotation: ||||
	2	[phq_1]	Little interest or pleasure in doing things:	radio (Matrix)<br>0 Not at all<br>1 Several days<br>2 More than half the days<br>3 Nearly every day<br>Field Annotation: % PHQ %|PHQ-9 Depression Scale (Patient Health Questionaire)||No|
	3	[phq_2]	Feeling down, depressed, or hopeless:	radio (Matrix)<br>0 Not at all<br>1 Several days<br>2 More than half the days<br>3 Nearly every day<br>Field Annotation: % PHQ %|PHQ-9 Depression Scale (Patient Health Questionaire)||No|
	4	[phq_3]	Trouble falling or staying asleep, or sleeping too much:	radio (Matrix)<br>0 Not at all<br>1 Several days<br>2 More than half the days<br>3 Nearly every day<br>Field Annotation: % PHQ %|PHQ-9 Depression Scale (Patient Health Questionaire)||No|
	5	[phq_4]	Feeling tired or having little energy:	radio (Matrix)<br>0 Not at all<br>1 Several days<br>2 More than half the days<br>3 Nearly every day<br>Field Annotation: % PHQ %|PHQ-9 Depression Scale (Patient Health Questionaire)||No|
	6	[phq_6]	Feeling bad about yourself, or that you are a failure, or have let yourself or your family down:	radio (Matrix)<br>0 Not at all<br>1 Several days<br>2 More than half the days<br>3 Nearly every day<br>Field Annotation: % PHQ %|PHQ-9 Depression Scale (Patient Health Questionaire)||No|
	7	[phq_7]	Trouble concentrating on things, such as reading the newspaper or watching television:	radio (Matrix)<br>0 Not at all<br>1 Several days<br>2 More than half the days<br>3 Nearly every day<br>Field Annotation: % PHQ %|PHQ-9 Depression Scale (Patient Health Questionaire)||No|

	8	[phq_8]	Moving or speaking so slowly that other people could have noticed. Or the opposite, being so fidgety or restless that you have been moving around a lot more than usual:	radio (Matrix)<br>0 Not at all<br>1 Several days<br>2 More than half the days<br>3 Nearly every day<br><br>Field Annotation: % PHQ %|PHQ-9 Depression Scale (Patient Health Questionaire)||No|
	9	[phq_9]	Thoughts that you would be better off dead, or of hurting yourself:	radio (Matrix)<br>0 Not at all<br>1 Several days<br>2 More than half the days<br>3 Nearly every day<br><br>Field Annotation: % PHQ %|PHQ-9 Depression Scale (Patient Health Questionaire)||No|
	10	[gad_intro]	Over the past two weeks, how often have you been bothered by the following problems:	descriptive<br>Field Annotation: ||||
	11	[gad_1]	Feeling nervous, anxious, or on edge:	radio (Matrix)<br>0 Not at all<br>1 Several days<br>2 More than half the days<br>3 Nearly every day<br><br>Field Annotation: % GAD %|Generalize Anxiety Disorder Assessment||No|
	12	[gad_2]	Not being able to stop or control worrying:	radio (Matrix)<br>0 Not at all<br>1 Several days<br>2 More than half the days<br>3 Nearly every day<br><br>Field Annotation: % GAD %|Generalize Anxiety Disorder Assessment||No|
	13	[gad_3]	Worrying too much about different things:	radio (Matrix)<br>0 Not at all<br>1 Several days<br>2 More than half the days<br>3 Nearly every day<br><br>Field Annotation: % GAD %|Generalize Anxiety Disorder Assessment||No|
	14	[gad_4]	Trouble relaxing:	radio (Matrix)<br>0 Not at all<br>1 Several days<br>2 More than half the days<br>3 Nearly every day<br><br>Field Annotation: % GAD %|Generalize Anxiety Disorder Assessment||No|
	15	[gad_5]	Being so restless that it is hard to sit still:	radio (Matrix)<br>0 Not at all<br>1 Several days<br>2 More than half the days<br>3 Nearly every day<br><br>Field Annotation: % GAD %|Generalize Anxiety Disorder Assessment||No|

	16	[gad_6]	Becoming easily annoyed or irritable:	radio (Matrix)<br>0 Not at all<br>1 Several days<br>2 More than half the days<br>3 Nearly every day<br><br>Field Annotation: % GAD %|Generalize Anxiety Disorder Assessment||No|
	17	[gad_7]	Feeling afraid as if something awful might happen:	radio (Matrix)<br>0 Not at all<br>1 Several days<br>2 More than half the days<br>3 Nearly every day<br><br>Field Annotation: % GAD %|Generalize Anxiety Disorder Assessment||No|
	18	[ps_main_intro]	Section Header:<br>Please tell us at what time(s) you have had any of the following symptoms. Check all that apply.	descriptive<br>Field Annotation: ||||
	19	[ps_fatigue_c13]	Fatigue (being very tired)	checkbox, Required<br>0 ps_fatigue_c13___0 No, I have NOT had this symptom<br>1 ps_fatigue_c13___1 Yes, I DID have it in the YEAR BEFORE [stem_my]<br>2 ps_fatigue_c13___2 Yes, I DID have it AROUND the time of [stem_my]<br>4 ps_fatigue_c13___4 Yes, I have it NOW<br>-88 ps_fatigue_c13____88 I don't know or prefer not to answer<br><br>Field Annotation: @NONEOFTHEABOVE="0,-88"|RECOVER|||
	20	[ps_malaise_c13]	Post-exertional malaise (Symptoms worse after even minor physical or mental effort)	checkbox, Required<br>0 ps_malaise_c13___0 No, I have NOT had this symptom<br>1 ps_malaise_c13___1 Yes, I DID have it in the YEAR BEFORE [stem_my]<br>2 ps_malaise_c13___2 Yes, I DID have it AROUND the time of [stem_my]<br>4 ps_malaise_c13___4 Yes, I have it NOW<br>-88 ps_malaise_c13____88 I don't know or prefer not to answer<br><br>Field Annotation: @NONEOFTHEABOVE="0,-88"|RECOVER|||
	21	[ps_fever_c13]	Fever, chills, sweats or flushing	checkbox, Required<br>0 ps_fever_c13___0 No, I have NOT had this symptom<br>1 ps_fever_c13___1 Yes, I DID have it in the YEAR BEFORE [stem_my]<br>2 ps_fever_c13___2 Yes, I DID have it AROUND the time of [stem_my]<br>4 ps_fever_c13___4 Yes, I have it NOW<br>-88 ps_fever_c13____88 I don't know or prefer not to answer<br><br>Field Annotation: @NONEOFTHEABOVE="0,-88"|RECOVER|||

<table>
<tr><td></td><td>22</td><td>[ps_sense_c13]</td><td>Loss of or change in smell or taste</td><td>checkbox, Required
<table>
<tr><td>0</td><td>ps_sense_c13___0</td><td>No, I have NOT had this symptom</td></tr>
<tr><td>1</td><td>ps_sense_c13___1</td><td>Yes, I DID have it in the YEAR BEFORE [stem_my]</td></tr>
<tr><td>2</td><td>ps_sense_c13___2</td><td>Yes, I DID have it AROUND the time of [stem_my]</td></tr>
<tr><td>4</td><td>ps_sense_c13___4</td><td>Yes, I have it NOW</td></tr>
<tr><td>-88</td><td>ps_sense_c13____88</td><td>I don't know or prefer not to answer</td></tr>
</table>
Field Annotation:
@NONEOFTHEABOVE="0,-88"|RECOVER|||</td></tr>
<tr><td></td><td>23</td><td>[ps_pain_c13]</td><td>Pain in any part of your body</td><td>checkbox, Required
<table>
<tr><td>0</td><td>ps_pain_c13___0</td><td>No, I have NOT had this symptom</td></tr>
<tr><td>1</td><td>ps_pain_c13___1</td><td>Yes, I DID have it in the YEAR BEFORE [stem_my]</td></tr>
<tr><td>2</td><td>ps_pain_c13___2</td><td>Yes, I DID have it AROUND the time of [stem_my]</td></tr>
<tr><td>4</td><td>ps_pain_c13___4</td><td>Yes, I have it NOW</td></tr>
<tr><td>-88</td><td>ps_pain_c13____88</td><td>I don't know or prefer not to answer</td></tr>
</table>
Field Annotation:
@NONEOFTHEABOVE="0,-88"|RECOVER|||</td></tr>
<tr><td></td><td>24</td><td>[ps_sob_c13]</td><td>Shortness of breath</td><td>checkbox, Required
<table>
<tr><td>0</td><td>ps_sob_c13___0</td><td>No, I have NOT had this symptom</td></tr>
<tr><td>1</td><td>ps_sob_c13___1</td><td>Yes, I DID have it in the YEAR BEFORE [stem_my]</td></tr>
<tr><td>2</td><td>ps_sob_c13___2</td><td>Yes, I DID have it AROUND the time of [stem_my]</td></tr>
<tr><td>4</td><td>ps_sob_c13___4</td><td>Yes, I have it NOW</td></tr>
<tr><td>-88</td><td>ps_sob_c13____88</td><td>I don't know or prefer not to answer</td></tr>
</table>
Field Annotation:
@NONEOFTHEABOVE="0,-88"|RECOVER|||</td></tr>
<tr><td></td><td>25</td><td>[ps_heart_c13]</td><td>Palpitations, racing heart, arrhythmia, skipped beats</td><td>checkbox, Required
<table>
<tr><td>0</td><td>ps_heart_c13___0</td><td>No, I have NOT had this symptom</td></tr>
<tr><td>1</td><td>ps_heart_c13___1</td><td>Yes, I DID have it in the YEAR BEFORE [stem_my]</td></tr>
<tr><td>2</td><td>ps_heart_c13___2</td><td>Yes, I DID have it AROUND the time of [stem_my]</td></tr>
<tr><td>4</td><td>ps_heart_c13___4</td><td>Yes, I have it NOW</td></tr>
<tr><td>-88</td><td>ps_heart_c13____88</td><td>I don't know or prefer not to answer</td></tr>
</table>
Field Annotation:
@NONEOFTHEABOVE="0,-88"|RECOVER|||</td></tr>
</table>

<table>
<tr><td></td><td>26</td><td>[ps_swelllegs_c13]</td><td>Swelling of your legs</td><td>checkbox, Required
<table>
<tr><td>0</td><td>ps_swelllegs_c13___0</td><td>No, I have NOT had this symptom</td></tr>
<tr><td>1</td><td>ps_swelllegs_c13___1</td><td>Yes, I DID have it in the YEAR BEFORE [stem_my]</td></tr>
<tr><td>2</td><td>ps_swelllegs_c13___2</td><td>Yes, I DID have it AROUND the time of [stem_my]</td></tr>
<tr><td>4</td><td>ps_swelllegs_c13___4</td><td>Yes, I have it NOW</td></tr>
<tr><td>-88</td><td>ps_swelllegs_c13____88</td><td>I don't know or prefer not to answer</td></tr>
</table>
Field Annotation:
@NONEOFTHEABOVE="0,-88"|RECOVER|||</td></tr>
<tr><td></td><td>27</td><td>[ps_gastro_c13]</td><td>Gastrointestinal (belly) symptoms (feeling full or vomiting after eating, diarrhea, constipation)</td><td>checkbox, Required
<table>
<tr><td>0</td><td>ps_gastro_c13___0</td><td>No, I have NOT had this symptom</td></tr>
<tr><td>1</td><td>ps_gastro_c13___1</td><td>Yes, I DID have it in the YEAR BEFORE [stem_my]</td></tr>
<tr><td>2</td><td>ps_gastro_c13___2</td><td>Yes, I DID have it AROUND the time of [stem_my]</td></tr>
<tr><td>4</td><td>ps_gastro_c13___4</td><td>Yes, I have it NOW</td></tr>
<tr><td>-88</td><td>ps_gastro_c13____88</td><td>I don't know or prefer not to answer</td></tr>
</table>
Field Annotation:
@NONEOFTHEABOVE="0,-88"|RECOVER|||</td></tr>
<tr><td></td><td>28</td><td>[ps_bladder_c13]</td><td>Bladder problems (incontinence, trouble passing urine or emptying bladder)</td><td>checkbox, Required
<table>
<tr><td>0</td><td>ps_bladder_c13___0</td><td>No, I have NOT had this symptom</td></tr>
<tr><td>1</td><td>ps_bladder_c13___1</td><td>Yes, I DID have it in the YEAR BEFORE [stem_my]</td></tr>
<tr><td>2</td><td>ps_bladder_c13___2</td><td>Yes, I DID have it AROUND the time of [stem_my]</td></tr>
<tr><td>4</td><td>ps_bladder_c13___4</td><td>Yes, I have it NOW</td></tr>
<tr><td>-88</td><td>ps_bladder_c13____88</td><td>I don't know or prefer not to answer</td></tr>
</table>
Field Annotation:
@NONEOFTHEABOVE="0,-88"|RECOVER|||</td></tr>
<tr><td></td><td>29</td><td>[ps_color_c13]</td><td>Color changes in your skin, such as red, white or purple</td><td>checkbox, Required
<table>
<tr><td>0</td><td>ps_color_c13___0</td><td>No, I have NOT had this symptom</td></tr>
<tr><td>1</td><td>ps_color_c13___1</td><td>Yes, I DID have it in the YEAR BEFORE [stem_my]</td></tr>
<tr><td>2</td><td>ps_color_c13___2</td><td>Yes, I DID have it AROUND the time of [stem_my]</td></tr>
<tr><td>4</td><td>ps_color_c13___4</td><td>Yes, I have it NOW</td></tr>
<tr><td>-88</td><td>ps_color_c13____88</td><td>I don't know or prefer not to answer</td></tr>
</table>
Field Annotation:
@NONEOFTHEABOVE="0,-88"|RECOVER|||</td></tr>
</table>

	30	[ps_rash_c13]	Skin rash	checkbox, Required<br>0 | ps_rash_c13___0 | No, I have NOT had this symptom<br>1 | ps_rash_c13___1 | Yes, I DID have it in the YEAR BEFORE [stem_my]<br>2 | ps_rash_c13___2 | Yes, I DID have it AROUND the time of [stem_my]<br>4 | ps_rash_c13___4 | Yes, I have it NOW<br>-88 | ps_rash_c13____88 | I don't know or prefer not to answer<br><br>Field Annotation: @NONEOFTHEABOVE="0,-88"|RECOVER|||
	31	[ps_drymouth_c13]	Excessively dry mouth	checkbox, Required<br>0 | ps_drymouth_c13___0 | No, I have NOT had this symptom<br>1 | ps_drymouth_c13___1 | Yes, I DID have it in the YEAR BEFORE [stem_my]<br>2 | ps_drymouth_c13___2 | Yes, I DID have it AROUND the time of [stem_my]<br>4 | ps_drymouth_c13___4 | Yes, I have it NOW<br>-88 | ps_drymouth_c13____88 | I don't know or prefer not to answer<br><br>Field Annotation: @NONEOFTHEABOVE="0,-88"|RECOVER|||
	32	[ps_thirst_c13]	Excessive thirst	checkbox, Required<br>0 | ps_thirst_c13___0 | No, I have NOT had this symptom<br>1 | ps_thirst_c13___1 | Yes, I DID have it in the YEAR BEFORE [stem_my]<br>2 | ps_thirst_c13___2 | Yes, I DID have it AROUND the time of [stem_my]<br>4 | ps_thirst_c13___4 | Yes, I have it NOW<br>-88 | ps_thirst_c13____88 | I don't know or prefer not to answer<br><br>Field Annotation: @NONEOFTHEABOVE="0,-88"|RECOVER|||
	33	[ps_vision_c13]	Vision problems (blurry, light sensitivity, difficulty reading or focusing, floaters, flashing lights, "snow")	checkbox, Required<br>0 | ps_vision_c13___0 | No, I have NOT had this symptom<br>1 | ps_vision_c13___1 | Yes, I DID have it in the YEAR BEFORE [stem_my]<br>2 | ps_vision_c13___2 | Yes, I DID have it AROUND the time of [stem_my]<br>4 | ps_vision_c13___4 | Yes, I have it NOW<br>-88 | ps_vision_c13____88 | I don't know or prefer not to answer<br><br>Field Annotation: @NONEOFTHEABOVE="0,-88"|RECOVER|||

	34	[ps_hearing_c13]	Problems with hearing (hearing loss, ringing in ears)	checkbox, Required<br>0 | ps_hearing_c13___0 | No, I have NOT had this symptom<br>1 | ps_hearing_c13___1 | Yes, I DID have it in the YEAR BEFORE [stem_my]<br>2 | ps_hearing_c13___2 | Yes, I DID have it AROUND the time of [stem_my]<br>4 | ps_hearing_c13___4 | Yes, I have it NOW<br>-88 | ps_hearing_c13____88 | I don't know or prefer not to answer<br><br>Field Annotation:<br>@NONEOFTHEABOVE="0,-88"|RECOVER|||
	35	[ps_bald_c13]	Hair loss	checkbox, Required<br>0 | ps_bald_c13___0 | No, I have NOT had this symptom<br>1 | ps_bald_c13___1 | Yes, I DID have it in the YEAR BEFORE [stem_my]<br>2 | ps_bald_c13___2 | Yes, I DID have it AROUND the time of [stem_my]<br>4 | ps_bald_c13___4 | Yes, I have it NOW<br>-88 | ps_bald_c13____88 | I don't know or prefer not to answer<br><br>Field Annotation:<br>@NONEOFTHEABOVE="0,-88"|RECOVER|||
	36	[ps_teeth_c13]	Problems with teeth	checkbox, Required<br>0 | ps_teeth_c13___0 | No, I have NOT had this symptom<br>1 | ps_teeth_c13___1 | Yes, I DID have it in the YEAR BEFORE [stem_my]<br>2 | ps_teeth_c13___2 | Yes, I DID have it AROUND the time of [stem_my]<br>4 | ps_teeth_c13___4 | Yes, I have it NOW<br>-88 | ps_teeth_c13____88 | I don't know or prefer not to answer<br><br>Field Annotation:<br>@NONEOFTHEABOVE="0,-88"|RECOVER|||
	37	[ps_menstrual_c13]	Changes to menstrual cycle	checkbox, Required<br>0 | ps_menstrual_c13___0 | No, I have NOT had this symptom<br>1 | ps_menstrual_c13___1 | Yes, I DID have it in the YEAR BEFORE [stem_my]<br>2 | ps_menstrual_c13___2 | Yes, I DID have it AROUND the time of [stem_my]<br>4 | ps_menstrual_c13___4 | Yes, I have it NOW<br>-88 | ps_menstrual_c13____88 | I don't know or prefer not to answer<br><br>Field Annotation:<br>@NONEOFTHEABOVE="0,-88"|RECOVER|||

	38	[ps_menopause_c13]	Changes to menopause symptoms	checkbox, Required<br>0 | ps_menopause_c13___0 | No, I have NOT had this symptom<br>1 | ps_menopause_c13___1 | Yes, I DID have it in the YEAR BEFORE [stem_my]<br>2 | ps_menopause_c13___2 | Yes, I DID have it AROUND the time of [stem_my]<br>4 | ps_menopause_c13___4 | Yes, I have it NOW<br>-88 | ps_menopause_c13____88 | I don't know or prefer not to answer<br><br>Field Annotation:<br>@NONEOFTHEABOVE="0,-88"|RECOVER|||
	39	[ps_fertility_c13]	Changes in fertility or difficulty getting pregnant	checkbox, Required<br>0 | ps_fertility_c13___0 | No, I have NOT had this symptom<br>1 | ps_fertility_c13___1 | Yes, I DID have it in the YEAR BEFORE [stem_my]<br>2 | ps_fertility_c13___2 | Yes, I DID have it AROUND the time of [stem_my]<br>4 | ps_fertility_c13___4 | Yes, I have it NOW<br>-88 | ps_fertility_c13____88 | I don't know or prefer not to answer<br><br>Field Annotation:<br>@NONEOFTHEABOVE="0,-88"|RECOVER|||
	40	[ps_sex_c13]	Changes in desire for, comfort with or capacity for sex	checkbox, Required<br>0 | ps_sex_c13___0 | No, I have NOT had this symptom<br>1 | ps_sex_c13___1 | Yes, I DID have it in the YEAR BEFORE [stem_my]<br>2 | ps_sex_c13___2 | Yes, I DID have it AROUND the time of [stem_my]<br>4 | ps_sex_c13___4 | Yes, I have it NOW<br>-88 | ps_sex_c13____88 | I don't know or prefer not to answer<br><br>Field Annotation:<br>@NONEOFTHEABOVE="0,-88"|RECOVER|||
	41	[ps_other]	Section Header: *Other symptoms:*<br>Have you experienced any other symptoms that you attribute to [stem_your]?	radio<br>1 | Yes<br>0 | No<br>-88 | I prefer not to answer<br><br>Custom alignment: LV<br>Field Annotation: |RECOVER|||
	42	[ps_otherspec]	Please specify any other symptoms that you attribute to [stem_your]:	notes<br>Custom alignment: LV<br>Field Annotation: |RECOVER|||
	43	[ps_pain_header]	Section Header:<br>This set of questions is about your problem with pain.	descriptive<br>Field Annotation: ||||

<table>
<tr><td></td><td>44</td><td>[ps_pain_select_b]</td><td>In the YEAR BEFORE [stem_your], where were you having pain? Check all that apply.</td><td>checkbox
<table>
<tr><td>1</td><td>ps_pain_select_b___1</td><td>Head pain/headache</td></tr>
<tr><td>2</td><td>ps_pain_select_b___2</td><td>Chest pain (including chest tightness, pressure)</td></tr>
<tr><td>3</td><td>ps_pain_select_b___3</td><td>Abdomen (belly)</td></tr>
<tr><td>4</td><td>ps_pain_select_b___4</td><td>Pelvis or genitals</td></tr>
<tr><td>5</td><td>ps_pain_select_b___5</td><td>Joints</td></tr>
<tr><td>6</td><td>ps_pain_select_b___6</td><td>Muscles</td></tr>
<tr><td>7</td><td>ps_pain_select_b___7</td><td>Back/spine</td></tr>
<tr><td>8</td><td>ps_pain_select_b___8</td><td>Skin</td></tr>
<tr><td>9</td><td>ps_pain_select_b___9</td><td>Feet</td></tr>
<tr><td>10</td><td>ps_pain_select_b___10</td><td>Mouth</td></tr>
<tr><td>11</td><td>ps_pain_select_b___11</td><td>Throat</td></tr>
</table>
Custom alignment: LV
Field Annotation: % NYU %|RECOVER|||</td></tr>
<tr><td></td><td>45</td><td>[ps_pain_select_a]</td><td>AROUND [stem_your], where were you having pain? Check all that apply.</td><td>checkbox
<table>
<tr><td>1</td><td>ps_pain_select_a___1</td><td>Head pain/headache</td></tr>
<tr><td>2</td><td>ps_pain_select_a___2</td><td>Chest pain (including chest tightness, pressure)</td></tr>
<tr><td>3</td><td>ps_pain_select_a___3</td><td>Abdomen (belly)</td></tr>
<tr><td>4</td><td>ps_pain_select_a___4</td><td>Pelvis or genitals</td></tr>
<tr><td>5</td><td>ps_pain_select_a___5</td><td>Joints</td></tr>
<tr><td>6</td><td>ps_pain_select_a___6</td><td>Muscles</td></tr>
<tr><td>7</td><td>ps_pain_select_a___7</td><td>Back/spine</td></tr>
<tr><td>8</td><td>ps_pain_select_a___8</td><td>Skin</td></tr>
<tr><td>9</td><td>ps_pain_select_a___9</td><td>Feet</td></tr>
<tr><td>10</td><td>ps_pain_select_a___10</td><td>Mouth</td></tr>
<tr><td>11</td><td>ps_pain_select_a___11</td><td>Throat</td></tr>
</table>
Custom alignment: LV
Field Annotation: % NYU %|RECOVER|||</td></tr>
<tr><td></td><td>46</td><td>[ps_pain_select_pa]</td><td>BETWEEN 30 DAYS AFTER [stem_your] AND NOW where were you having pain? Check all that apply</td><td>checkbox
<table>
<tr><td>1</td><td>ps_pain_select_pa___1</td><td>Head pain/headache</td></tr>
<tr><td>2</td><td>ps_pain_select_pa___2</td><td>Chest pain (including chest tightness, pressure)</td></tr>
<tr><td>3</td><td>ps_pain_select_pa___3</td><td>Abdomen (belly)</td></tr>
<tr><td>4</td><td>ps_pain_select_pa___4</td><td>Pelvis or genitals</td></tr>
<tr><td>5</td><td>ps_pain_select_pa___5</td><td>Joints</td></tr>
<tr><td>6</td><td>ps_pain_select_pa___6</td><td>Muscles</td></tr>
<tr><td>7</td><td>ps_pain_select_pa___7</td><td>Back/spine</td></tr>
<tr><td>8</td><td>ps_pain_select_pa___8</td><td>Skin</td></tr>
<tr><td>9</td><td>ps_pain_select_pa___9</td><td>Feet</td></tr>
<tr><td>10</td><td>ps_pain_select_pa___10</td><td>Mouth</td></tr>
<tr><td>11</td><td>ps_pain_select_pa___11</td><td>Throat</td></tr>
</table>
Custom alignment: LV
Field Annotation: % NYU %|RECOVER|||</td></tr>
</table>

	47	[ps_pain_select_funl]	In [stem_the], where were you having pain that you no longer have? Check all that apply.	checkbox<br>1 ps_pain_select_funl___1 Head pain/headache<br>2 ps_pain_select_funl___2 Chest pain (including chest tightness, pressure)<br>3 ps_pain_select_funl___3 Abdomen (belly)<br>4 ps_pain_select_funl___4 Pelvis or genitals<br>5 ps_pain_select_funl___5 Joints<br>6 ps_pain_select_funl___6 Muscles<br>7 ps_pain_select_funl___7 Back/spine<br>8 ps_pain_select_funl___8 Skin<br>9 ps_pain_select_funl___9 Feet<br>10 ps_pain_select_funl___10 Mouth<br>11 ps_pain_select_funl___11 Throat<br><br>Custom alignment: LV<br>Field Annotation: % NYU %|RECOVER|||
	48	[ps_pain_select]	Where are you having pain RIGHT NOW? Check all that apply.	checkbox<br>1 ps_pain_select___1 Head pain/headache<br>2 ps_pain_select___2 Chest pain (including chest tightness, pressure)<br>3 ps_pain_select___3 Abdomen (belly)<br>4 ps_pain_select___4 Pelvis or genitals<br>5 ps_pain_select___5 Joints<br>6 ps_pain_select___6 Muscles<br>7 ps_pain_select___7 Back/spine<br>8 ps_pain_select___8 Skin<br>9 ps_pain_select___9 Feet<br>10 ps_pain_select___10 Mouth<br>11 ps_pain_select___11 Throat<br><br>Custom alignment: LV<br>Field Annotation: % NYU %|RECOVER|||
	49	[ps_headache_intro]	Section Header:<br>This set of questions is about your headaches:	descriptive<br>Field Annotation: ||||
	50	[hit6_severe]	When you have headaches, how often is the pain severe?	radio (Matrix)<br>1 Never<br>2 Rarely<br>3 Sometimes<br>4 Very often<br>5 Always<br><br>Field Annotation: % HIT-6 https://www.qualitymetric.com/health-surveys-old/the-headache-impact-test-hit-6/ %|The Headache Impact Test� (HIT-6�)||No|
	51	[hit6_activities]	How often do headaches limit your ability to do usual daily activities including household work, work, school, or social activities?	radio (Matrix)<br>1 Never<br>2 Rarely<br>3 Sometimes<br>4 Very often<br>5 Always<br><br>Field Annotation: % HIT-6 https://www.qualitymetric.com/health-surveys-old/the-headache-impact-test-hit-6/ %|The Headache Impact Test� (HIT-6�)||No|

	52	[hit6_liedown]	When you have a headache, how often do you wish you could lie down?	radio (Matrix)<br>1 Never<br>2 Rarely<br>3 Sometimes<br>4 Very often<br>5 Always<br><br>Field Annotation: % HIT-6 https://www.qualitymetric.com/health-surveys-old/the-headache-impact-test-hit-6/ %|The Headache Impact Test� (HIT-6�)||No|
	53	[hit6_tootired]	In the past 4 weeks, how often have you felt too tired to do work or daily activities because of your headaches?	radio (Matrix)<br>1 Never<br>2 Rarely<br>3 Sometimes<br>4 Very often<br>5 Always<br><br>Field Annotation: % HIT-6 https://www.qualitymetric.com/health-surveys-old/the-headache-impact-test-hit-6/ %|The Headache Impact Test� (HIT-6�)||No|
	54	[hit6_concentrate]	In the past 4 weeks, how often did headaches limit your ability to concentrate on work or daily activities?	radio (Matrix)<br>1 Never<br>2 Rarely<br>3 Sometimes<br>4 Very often<br>5 Always<br><br>Field Annotation: % HIT-6 https://www.qualitymetric.com/health-surveys-old/the-headache-impact-test-hit-6/ %|The Headache Impact Test� (HIT-6�)||No|
	55	[hit6_irritated]	In the past 4 weeks, how often have you felt fed up or irritated because of your headaches	radio (Matrix)<br>1 Never<br>2 Rarely<br>3 Sometimes<br>4 Very often<br>5 Always<br><br>Field Annotation: % HIT-6 https://www.qualitymetric.com/health-surveys-old/the-headache-impact-test-hit-6/ %|The Headache Impact Test� (HIT-6�)||No|
	56	[ps_chestpain_intro]	Section Header:<br>This set of questions is about your chest pain.	descriptive<br>Field Annotation: ||||
	57	[saq_act_intro]	The following is a list of activities that people often do during the week. Although for some people with several medical problems it is difficult to determine what it is that limits them, please go over the activities listed below and indicate how much limitation you have had due to chest pain, chest tightness, or angina over the past 4 weeks.	descriptive<br>Field Annotation: % Seattle Angina Questionnaire https://www.ncbi.nlm.nih.gov/pmc/articles/PMC4282595/ %|Seattle Angina Questionnaire|||

	58	[saq_actwalk]	Walking indoors on level ground	radio (Matrix)<br>1 Extremely limited<br>2 Quite a bit limited<br>3 Moderately limited<br>4 Slightly limited<br>5 Not at all limited<br>6 Limited for other reasons or did not do the activity<br><br>Field Annotation: % Seattle Angina Questionnaire https://www.ncbi.nlm.nih.gov/pmc/articles/PMC4282595/ %|Seattle Angina Questionnaire||No|
	59	[saq_actgarden]	Gardening, vacuuming, or carrying groceries	radio (Matrix)<br>1 Extremely limited<br>2 Quite a bit limited<br>3 Moderately limited<br>4 Slightly limited<br>5 Not at all limited<br>6 Limited for other reasons or did not do the activity<br><br>Field Annotation: % Seattle Angina Questionnaire https://www.ncbi.nlm.nih.gov/pmc/articles/PMC4282595/ %|Seattle Angina Questionnaire||No|
	60	[saq_actlift]	Lifting or moving heavy objects (e.g. furniture, children)	radio (Matrix)<br>1 Extremely limited<br>2 Quite a bit limited<br>3 Moderately limited<br>4 Slightly limited<br>5 Not at all limited<br>6 Limited for other reasons or did not do the activity<br><br>Field Annotation: % Seattle Angina Questionnaire https://www.ncbi.nlm.nih.gov/pmc/articles/PMC4282595/ %|Seattle Angina Questionnaire||No|
	61	[saq_chestpain]	Over the past 4 weeks, on average, how many times have you had chest pain, chest tightness, or angina?	radio<br>1 4 or more times per day<br>2 1-3 times per day<br>3 3 or more times per week but not every day<br>4 1-2 times per week<br>5 Less than once a week<br>6 None over the past 4 weeks<br><br>Custom alignment: LV<br>Field Annotation: % Seattle Angina Questionnaire https://www.ncbi.nlm.nih.gov/pmc/articles/PMC4282595/ %|Seattle Angina Questionnaire||No|
	62	[saq_nitroglycerin]	Over the past 4 weeks, on average, how many times have you had to take nitroglycerin (tablets or spray) for your chest pain, chest tightness, or angina?	radio<br>1 4 or more times per day<br>2 1-3 times per day<br>3 3 or more times per week but not every day<br>4 1-2 times per week<br>5 Less than once a week<br>6 None over the past 4 weeks<br><br>Custom alignment: LV<br>Field Annotation: % Seattle Angina Questionnaire https://www.ncbi.nlm.nih.gov/pmc/articles/PMC4282595/ %|Seattle Angina Questionnaire||No|

	63	[saq_enjoyment]	Over the past 4 weeks, how much has your chest pain, chest tightness, or angina limited your enjoyment of life?	radio<br>1 It has extremely limited my enjoyment of life<br>2 It has limited my enjoyment of life quite a bit<br>3 It has moderately limited my enjoyment of life<br>4 It has slightly limited my enjoyment of life<br>5 It has not limited my enjoyment of life at all<br><br>Custom alignment: LV<br>Field Annotation: % Seattle Angina Questionnaire https://www.ncbi.nlm.nih.gov/pmc/articles/PMC4282595/ %|Seattle Angina Questionnaire||No|
	64	[saq_restoflife]	If you had to spend the rest of your life with your chest pain, chest tightness, or angina the way it is right now, how would you feel about this?	radio<br>1 Not satisfied at all<br>2 Mostly dissatisfied<br>3 Somewhat satisfied<br>4 Mostly satisfied<br>5 Completely satisfied<br><br>Custom alignment: LV<br>Field Annotation: % Seattle Angina Questionnaire https://www.ncbi.nlm.nih.gov/pmc/articles/PMC4282595/ %|Seattle Angina Questionnaire||No|
	65	[ps_sob_header]	Section Header:<br>This set of questions is about your problem with shortness of breath.	descriptive<br>Field Annotation: ||||
	66	[mmrc_dyspnea]	Which of the following best describes your shortness of breath?	radio<br>0 I only get breathless with strenuous exercise.<br>1 I get short of breath when hurrying on the level or walking up a slight hill.<br>2 I walk slower than people of the same age on the level because of breathlessness, or I have to stop for breath when walking on my own pace on the level.<br>3 I stop for breath after walking about 100 meters or after a few minutes on the level.<br>4 I am too breathless to leave the house or I am breathless when dressing or undressing.<br><br>Custom alignment: LV<br>Field Annotation: % mMRC dyspnea scale https://www.mdcalc.com/mmrc-modified-medical-research-council-dyspnea-scale %|mMRC (Modified Medical Research Council) Dyspnea Scale|||No
	67	[ps_nerve_header]	Section Header:<br>This set of questions is about your nerve problems.	descriptive<br>Field Annotation: ||||
	68	[nerve_which_b]	In the YEAR BEFORE [stem_your], which nerve problems did you have? Check all that apply.	checkbox<br>1 nerve_which_b___1 Tremor<br>2 nerve_which_b___2 Abnormal movements<br>3 nerve_which_b___3 Numbness, tingling, burning<br>4 nerve_which_b___4 Inability to move part of body<br>5 nerve_which_b___5 Seizures<br><br>Custom alignment: LV<br>Field Annotation: % NYU %|RECOVER|||
	69	[nerve_which_a]	AROUND [stem_your], which nerve problems did you have? Check all that apply.	checkbox<br>1 nerve_which_a___1 Tremor<br>2 nerve_which_a___2 Abnormal movements<br>3 nerve_which_a___3 Numbness, tingling, burning<br>4 nerve_which_a___4 Inability to move part of body<br>5 nerve_which_a___5 Seizures<br><br>Custom alignment: LV<br>Field Annotation: % NYU %|RECOVER|||

	70	[nerve_which_pa]	BETWEEN 30 DAY AFTER [stem_your] AND NOW, which nerve problems did you have? Check all that apply.	checkbox<br>1 nerve_which_pa___1 Tremor<br>2 nerve_which_pa___2 Abnormal movements<br>3 nerve_which_pa___3 Numbness, tingling, burning<br>4 nerve_which_pa___4 Inability to move part of body<br>5 nerve_which_pa___5 Seizures<br><br>Custom alignment: LV<br>Field Annotation: % NYU %|RECOVER|||
	71	[nerve_which_funl]	In [stem_the], which nerve problems did you have that you no longer have? Check all that apply.	checkbox<br>1 nerve_which_funl___1 Tremor<br>2 nerve_which_funl___2 Abnormal movements<br>3 nerve_which_funl___3 Numbness, tingling, burning<br>4 nerve_which_funl___4 Inability to move part of body<br>5 nerve_which_funl___5 Seizures<br><br>Custom alignment: LV<br>Field Annotation: % NYU %|RECOVER|||
	72	[nerve_which]	Which nerve problems do you have right now? Check all that apply.	checkbox<br>1 nerve_which___1 Tremor<br>2 nerve_which___2 Abnormal movements<br>3 nerve_which___3 Numbness, tingling, burning<br>4 nerve_which___4 Inability to move part of body<br>5 nerve_which___5 Seizures<br><br>Custom alignment: LV<br>Field Annotation: % NYU %|RECOVER|||
	73	[mi_neuro_screen]	Please take a few minutes to answer the following questions about the feeling in your legs and feet. Check yes or no based on how you usually feel. Thank you.	descriptive<br>Field Annotation:<br>#michigan_neuropathy_screening_instrument#%<br>https://www.ncbi.nlm.nih.gov/pmc/articles/PMC3641573/<br>%|Michigan Neuropathy Screening Instrument|||
	74	[mi_neuro_1]	Are your legs and/or feet numb?	radio (Matrix)<br>1 Yes<br>2 No<br><br>Field Annotation:<br>#michigan_neuropathy_screening_instrument#%<br>https://www.ncbi.nlm.nih.gov/pmc/articles/PMC3641573/<br>%|Michigan Neuropathy Screening Instrument||No|
	75	[mi_neuro_2]	Do you ever have any burning pain in your legs and/or feet?	radio (Matrix)<br>1 Yes<br>2 No<br><br>Field Annotation:<br>#michigan_neuropathy_screening_instrument#%<br>https://www.ncbi.nlm.nih.gov/pmc/articles/PMC3641573/<br>%|Michigan Neuropathy Screening Instrument||No|
	76	[mi_neuro_3]	Are your feet too sensitive to touch?	radio (Matrix)<br>1 Yes<br>2 No<br><br>Field Annotation:<br>#michigan_neuropathy_screening_instrument#%<br>https://www.ncbi.nlm.nih.gov/pmc/articles/PMC3641573/<br>%|Michigan Neuropathy Screening Instrument||No|

	77	[mi_neuro_4]	Do you get muscle cramps in your legs and/or feet?	radio (Matrix)<br>1 Yes<br>2 No<br><br>Field Annotation:<br>#michigan_neuropathy_screening_instrument#%<br>https://www.ncbi.nlm.nih.gov/pmc/articles/PMC3641573/<br>%|Michigan Neuropathy Screening Instrument||No|
	78	[mi_neuro_5]	Do you ever have any prickling feelings in your legs or feet?	radio (Matrix)<br>1 Yes<br>2 No<br><br>Field Annotation:<br>#michigan_neuropathy_screening_instrument#%<br>https://www.ncbi.nlm.nih.gov/pmc/articles/PMC3641573/<br>%|Michigan Neuropathy Screening Instrument||No|
	79	[mi_neuro_6]	Does it hurt when the bed covers touch your skin?	radio (Matrix)<br>1 Yes<br>2 No<br><br>Field Annotation:<br>#michigan_neuropathy_screening_instrument#%<br>https://www.ncbi.nlm.nih.gov/pmc/articles/PMC3641573/<br>%|Michigan Neuropathy Screening Instrument||No|
	80	[mi_neuro_7]	When you get into the tub or shower, are you able to tell the hot water from the cold water?	radio (Matrix)<br>1 Yes<br>2 No<br><br>Field Annotation:<br>#michigan_neuropathy_screening_instrument#%<br>https://www.ncbi.nlm.nih.gov/pmc/articles/PMC3641573/<br>%|Michigan Neuropathy Screening Instrument||No|
	81	[mi_neuro_8]	Have you ever had an open sore on your foot?	radio (Matrix)<br>1 Yes<br>2 No<br><br>Field Annotation:<br>#michigan_neuropathy_screening_instrument#%<br>https://www.ncbi.nlm.nih.gov/pmc/articles/PMC3641573/<br>%|Michigan Neuropathy Screening Instrument||No|
	82	[mi_neuro_9]	Has your doctor ever told you that you have diabetic neuropathy?	radio (Matrix)<br>1 Yes<br>2 No<br><br>Field Annotation:<br>#michigan_neuropathy_screening_instrument#%<br>https://www.ncbi.nlm.nih.gov/pmc/articles/PMC3641573/<br>%|Michigan Neuropathy Screening Instrument||No|
	83	[mi_neuro_10]	Do you feel weak all over most of the time?	radio (Matrix)<br>1 Yes<br>2 No<br><br>Field Annotation:<br>#michigan_neuropathy_screening_instrument#%<br>https://www.ncbi.nlm.nih.gov/pmc/articles/PMC3641573/<br>%|Michigan Neuropathy Screening Instrument||No|
	84	[mi_neuro_11]	Are your symptoms worse at night?	radio (Matrix)<br>1 Yes<br>2 No<br><br>Field Annotation:<br>#michigan_neuropathy_screening_instrument#%<br>https://www.ncbi.nlm.nih.gov/pmc/articles/PMC3641573/<br>%|Michigan Neuropathy Screening Instrument||No|

	85	[mi_neuro_12]	Do your legs hurt when you walk?	radio (Matrix)<br>1 Yes<br>2 No<br><br>Field Annotation: #michigan_neuropathy_screening_instrument#% https://www.ncbi.nlm.nih.gov/pmc/articles/PMC3641573/ %|Michigan Neuropathy Screening Instrument||No|
	86	[mi_neuro_13]	Are you able to sense your feet when you walk?	radio (Matrix)<br>1 Yes<br>2 No<br><br>Field Annotation: #michigan_neuropathy_screening_instrument#% https://www.ncbi.nlm.nih.gov/pmc/articles/PMC3641573/ %|Michigan Neuropathy Screening Instrument||No|
	87	[mi_neuro_14]	Is the skin on your feet so dry that it cracks open?	radio (Matrix)<br>1 Yes<br>2 No<br><br>Field Annotation: #michigan_neuropathy_screening_instrument#% https://www.ncbi.nlm.nih.gov/pmc/articles/PMC3641573/ %|Michigan Neuropathy Screening Instrument||No|
	88	[mi_neuro_15]	Have you ever had an amputation?	radio (Matrix)<br>1 Yes<br>2 No<br><br>Field Annotation: #michigan_neuropathy_screening_instrument#% https://www.ncbi.nlm.nih.gov/pmc/articles/PMC3641573/ %|Michigan Neuropathy Screening Instrument||No|
	89	[ps_weaknerve_header]	Section Header:<br>This set of questions is about your problems with weakness in your arms or legs, or with numbness and tingling.	descriptive<br>Field Annotation: ||||
	90	[promis_pfa11]	Are you able to do chores such as vacuuming or yard work?	radio (Matrix)<br>5 Without any difficulty<br>4 With a little difficulty<br>3 With some difficulty<br>2 With much difficulty<br>1 Unable to do<br><br>Field Annotation: # promis physical function sf4a #|Patient-Reported Outcomes Measurement Information System (PROMIS) - Shortform|Physical Function 4a||
	91	[promis_pfa21]	Are you able to go up and down stairs at a normal pace?	radio (Matrix)<br>5 Without any difficulty<br>4 With a little difficulty<br>3 With some difficulty<br>2 With much difficulty<br>1 Unable to do<br><br>Field Annotation: # promis physical function sf4a #|Patient-Reported Outcomes Measurement Information System (PROMIS) - Shortform|Physical Function 4a||

	92	[promis_pfa23]	Are you able to go for a walk of at least 15 minutes?	radio (Matrix)<br>5 Without any difficulty<br>4 With a little difficulty<br>3 With some difficulty<br>2 With much difficulty<br>1 Unable to do<br><br>Field Annotation: # promis physical function sf4a #|Patient-Reported Outcomes Measurement Information System (PROMIS) - Shortform|Physical Function 4a||
	93	[promis_pfa53]	Are you able to run errands and shop?	radio (Matrix)<br>5 Without any difficulty<br>4 With a little difficulty<br>3 With some difficulty<br>2 With much difficulty<br>1 Unable to do<br><br>Field Annotation: # promis physical function sf4a #|Patient-Reported Outcomes Measurement Information System (PROMIS) - Shortform|Physical Function 4a||
	94	[neuroqol_pfa40]	Are you able to turn a key in a lock?	radio (Matrix)<br>5 Without any difficulty<br>4 With a little difficulty<br>3 With some difficulty<br>2 With much difficulty<br>1 Unable to do<br><br>Field Annotation: #neuroqol_sf_v10_uex_fnct_finemotor_adl#% In REDCap: Neuro-QOL SF v1.0 - UEx Fnct (FineMotor, ADL) %|Neuro-QOL|Fine Motor Skills|No|
	95	[neuroqol_pfa50]	Are you able to brush your teeth?	radio (Matrix)<br>5 Without any difficulty<br>4 With a little difficulty<br>3 With some difficulty<br>2 With much difficulty<br>1 Unable to do<br><br>Field Annotation: #neuroqol_sf_v10_uex_fnct_finemotor_adl#% In REDCap: Neuro-QOL SF v1.0 - UEx Fnct (FineMotor, ADL) %|Neuro-QOL|Fine Motor Skills|No|
	96	[neuroqol_nquex44]	Are you able to make a phone call using a touch tone key-pad?	radio (Matrix)<br>5 Without any difficulty<br>4 With a little difficulty<br>3 With some difficulty<br>2 With much difficulty<br>1 Unable to do<br><br>Field Annotation: #neuroqol_sf_v10_uex_fnct_finemotor_adl#% In REDCap: Neuro-QOL SF v1.0 - UEx Fnct (FineMotor, ADL) %|Neuro-QOL|Fine Motor Skills|No|

	97	[neuroqol_pfb21]	Are you able to pick up coins from a table top?	radio (Matrix)<br>5 Without any difficulty<br>4 With a little difficulty<br>3 With some difficulty<br>2 With much difficulty<br>1 Unable to do<br><br>Field Annotation: #neuroqol_sf_v10_uex_fnct_finemotor_adl#% In REDCap: Neuro-QOL SF v1.0 - UEx Fnct (FineMotor, ADL) %|Neuro-QOL|Fine Motor Skills|No|
	98	[neuroqol_pfa43]	Are you able to write with a pen or pencil?	radio (Matrix)<br>5 Without any difficulty<br>4 With a little difficulty<br>3 With some difficulty<br>2 With much difficulty<br>1 Unable to do<br><br>Field Annotation: #neuroqol_sf_v10_uex_fnct_finemotor_adl#% In REDCap: Neuro-QOL SF v1.0 - UEx Fnct (FineMotor, ADL) %|Neuro-QOL|Fine Motor Skills|No|
	99	[neuroqol_pfa35]	Are you able to open and close a zipper?	radio (Matrix)<br>5 Without any difficulty<br>4 With a little difficulty<br>3 With some difficulty<br>2 With much difficulty<br>1 Unable to do<br><br>Field Annotation: #neuroqol_sf_v10_uex_fnct_finemotor_adl#% In REDCap: Neuro-QOL SF v1.0 - UEx Fnct (FineMotor, ADL) %|Neuro-QOL|Fine Motor Skills|No|
	100	[neuroqol_pfa55]	Are you able to wash and dry your body?	radio (Matrix)<br>5 Without any difficulty<br>4 With a little difficulty<br>3 With some difficulty<br>2 With much difficulty<br>1 Unable to do<br><br>Field Annotation: #neuroqol_sf_v10_uex_fnct_finemotor_adl#% In REDCap: Neuro-QOL SF v1.0 - UEx Fnct (FineMotor, ADL) %|Neuro-QOL|Fine Motor Skills|No|
	101	[neuroqol_pfb26]	Are you able to shampoo your hair?	radio (Matrix)<br>5 Without any difficulty<br>4 With a little difficulty<br>3 With some difficulty<br>2 With much difficulty<br>1 Unable to do<br><br>Field Annotation: #neuroqol_sf_v10_uex_fnct_finemotor_adl#% In REDCap: Neuro-QOL SF v1.0 - UEx Fnct (FineMotor, ADL) %|Neuro-QOL|Fine Motor Skills|No|
	102	[ps_think_header]	Section Header:<br>This set of questions is about your problems with thinking or concentrating ("brain fog").	descriptive<br>Field Annotation: ||||
	103	[neuroqol_cog7day_intro]	In the past 7 days:	descriptive<br>Field Annotation: ||||

	104	[nqcog_nqcog64r1]	I had to read something several times to understand it:	radio (Matrix)<br>5 Never<br>4 Rarely (once)<br>3 Sometimes (2-3 times)<br>2 Often (once a day)<br>1 Very often (several times a day)<br><br>Field Annotation: % Neuro-QoL v2.0 Cognition Short Form %|Neuro-QOL v2.0|Cognitive Function|No|
	105	[nqcog_nqcog75r1]	My thinking was slow:	radio (Matrix)<br>5 Never<br>4 Rarely (once)<br>3 Sometimes (2-3 times)<br>2 Often (once a day)<br>1 Very often (several times a day)<br><br>Field Annotation: % Neuro-QoL v2.0 Cognition Short Form %|Neuro-QOL v2.0|Cognitive Function|No|
	106	[nqcog_nqcog77r1]	I had to work really hard to pay attention or I would make a mistake:	radio (Matrix)<br>5 Never<br>4 Rarely (once)<br>3 Sometimes (2-3 times)<br>2 Often (once a day)<br>1 Very often (several times a day)<br><br>Field Annotation: % Neuro-QoL v2.0 Cognition Short Form %|Neuro-QOL v2.0|Cognitive Function|No|
	107	[nqcog_nqcog80r1]	I had trouble concentrating:	radio (Matrix)<br>5 Never<br>4 Rarely (once)<br>3 Sometimes (2-3 times)<br>2 Often (once a day)<br>1 Very often (several times a day)<br><br>Field Annotation: % Neuro-QoL v2.0 Cognition Short Form %|Neuro-QOL v2.0|Cognitive Function|No|
	108	[neuroqol_cogdiff_intro]	How much difficulty do you currently have:	descriptive<br>Field Annotation: ||||
	109	[nqcog_nqcog22r1]	reading and following complex interactions (e.g., directions for a new medication)?	radio (Matrix)<br>5 None<br>4 A little<br>3 Somewhat<br>2 A lot<br>1 Cannot do<br><br>Field Annotation: % Neuro-QoL v2.0 Cognition Short Form %|Neuro-QOL v2.0|Cognitive Function|No|
	110	[nqcog_nqcog24r1]	planning for and keeping appointments that are not part of your weekly routine (e.g. a therapy or doctor appointment, or a social gather with friends and family)?	radio (Matrix)<br>5 None<br>4 A little<br>3 Somewhat<br>2 A lot<br>1 Cannot do<br><br>Field Annotation: % Neuro-QoL v2.0 Cognition Short Form %|Neuro-QOL v2.0|Cognitive Function|No|

	111	[nqcog_nqcog25r1]	managing your time to do most of your daily activities?	radio (Matrix)<br>5 None<br>4 A little<br>3 Somewhat<br>2 A lot<br>1 Cannot do<br><br>Field Annotation: % Neuro-QoL v2.0 Cognition Short Form %|Neuro-QOL v2.0|Cognitive Function|No|
	112	[nqcog_nqcog40r1]	learning new tasks or instructions?	radio (Matrix)<br>5 None<br>4 A little<br>3 Somewhat<br>2 A lot<br>1 Cannot do<br><br>Field Annotation: % Neuro-QoL v2.0 Cognition Short Form %|Neuro-QOL v2.0|Cognitive Function|No|
	113	[ps_sleep_header]	Section Header: *Problems with sleep:*<br>This set of questions is about your problems with sleep.	descriptive<br>Field Annotation: ||||
	114	[snore]	Has anyone ever told you that you have sleep apnea or that you snore?	radio<br>1 Yes<br>0 No<br>-88 Prefer not to answer<br><br>Custom alignment: LV<br>Field Annotation: |RECOVER|||
	115	[promis_sleep1_intro]	In the past 7 days...	descriptive<br>Field Annotation: ||||
	116	[promis_sleep109]	My sleep quality was	radio (Matrix)<br>1 Very poor<br>2 Poor<br>3 Fair<br>4 Good<br>5 Very good<br><br>Field Annotation: % PROMIS Sleep Disturbance 8a https://www.healthmeasures.net/index.php?option=com_instruments&view=measure&id=183&Itemid=99 %|Patient-Reported Outcomes Measurement Information System (PROMIS)|Sleep Disturbance||
	117	[promis_sleep2_intro]	In the past 7 days...	descriptive<br>Field Annotation: ||||
	118	[promis_sleep116]	My sleep was refreshing	radio (Matrix)<br>1 Not at all<br>2 A little bit<br>3 Somewhat<br>4 Quite a bit<br>5 Very much<br><br>Field Annotation: % PROMIS Sleep Disturbance 8a https://www.healthmeasures.net/index.php?option=com_instruments&view=measure&id=183&Itemid=99 %|Patient-Reported Outcomes Measurement Information System (PROMIS)|Sleep Disturbance||

	119	[promis_sleep20]	I had a problem with my sleep	radio (Matrix)<br>1 Not at all<br>2 A little bit<br>3 Somewhat<br>4 Quite a bit<br>5 Very much<br><br>Field Annotation: % PROMIS Sleep Disturbance 8a https://www.healthmeasures.net/index.php?option=com_instruments&view=measure&id=183&Itemid=99 %|Patient-Reported Outcomes Measurement Information System (PROMIS)|Sleep Distrubance||
	120	[promis_sleep44]	I had difficulty falling asleep	radio (Matrix)<br>1 Not at all<br>2 A little bit<br>3 Somewhat<br>4 Quite a bit<br>5 Very much<br><br>Field Annotation: % PROMIS Sleep Disturbance 8a https://www.healthmeasures.net/index.php?option=com_instruments&view=measure&id=183&Itemid=99 %|Patient-Reported Outcomes Measurement Information System (PROMIS)|Sleep Distrubance||
	121	[promis_sleep108]	My sleep was restless	radio (Matrix)<br>1 Not at all<br>2 A little bit<br>3 Somewhat<br>4 Quite a bit<br>5 Very much<br><br>Field Annotation: % PROMIS Sleep Disturbance 8a https://www.healthmeasures.net/index.php?option=com_instruments&view=measure&id=183&Itemid=99 %|Patient-Reported Outcomes Measurement Information System (PROMIS)|Sleep Distrubance||
	122	[promis_sleep72]	I tried hard to get to sleep	radio (Matrix)<br>1 Not at all<br>2 A little bit<br>3 Somewhat<br>4 Quite a bit<br>5 Very much<br><br>Field Annotation: % PROMIS Sleep Disturbance 8a https://www.healthmeasures.net/index.php?option=com_instruments&view=measure&id=183&Itemid=99 %|Patient-Reported Outcomes Measurement Information System (PROMIS)|Sleep Distrubance||
	123	[promis_sleep67]	I worried about not being able to fall asleep	radio (Matrix)<br>1 Not at all<br>2 A little bit<br>3 Somewhat<br>4 Quite a bit<br>5 Very much<br><br>Field Annotation: % PROMIS Sleep Disturbance 8a https://www.healthmeasures.net/index.php?option=com_instruments&view=measure&id=183&Itemid=99 %|Patient-Reported Outcomes Measurement Information System (PROMIS)|Sleep Distrubance||

	124	[promis_sleep115]	I was satisfied with my sleep	radio (Matrix)<br>1 Not at all<br>2 A little bit<br>3 Somewhat<br>4 Quite a bit<br>5 Very much<br><br>Field Annotation: % PROMIS Sleep Disturbance 8a https://www.healthmeasures.net/index.php?option=com_instruments&view=measure&id=183&Itemid=99 %|Patient-Reported Outcomes Measurement Information System (PROMIS)|Sleep Distrubance||
	125	[ps_vision_header]	Section Header:<br>This set of questions is about your problems with vision.	descriptive<br>Field Annotation: ||||
	126	[vfq_2]	At the present time, would you say your eyesight using both eyes (with glasses or contact lenses, if you wear them) is excellent, good, fair, poor, or very poor or are you completely blind?	radio<br>1 Excellent<br>2 Good<br>3 Fair<br>4 Poor<br>5 Very Poor<br>6 Completely Blind<br><br>Custom alignment: LV<br>Field Annotation: #national_eye_institute_visual_functioning_question#% https://www.nei.nih.gov/sites/default/files/2019-06/vfq_sa.pdf %|National Eye Institute Visual Functioning Questionnaire - 25 (VFQ-25)||No|
	127	[vfq_3]	How much of the time do you worry about your eyesight?	radio<br>1 None of the time<br>2 A little of the time<br>3 Some of the time<br>4 Most of the time<br>5 All of the time<br><br>Custom alignment: LV<br>Field Annotation: #national_eye_institute_visual_functioning_question#% https://www.nei.nih.gov/sites/default/files/2019-06/vfq_sa.pdf %|National Eye Institute Visual Functioning Questionnaire - 25 (VFQ-25)||No|
	128	[vfq_4]	How much pain or discomfort have you had in and around your eyes (for example, burning, itching, or aching)? Would you say it is:	radio<br>1 None<br>2 Mild<br>3 Moderate<br>4 Severe<br>5 Very severe<br><br>Custom alignment: LV<br>Field Annotation: #national_eye_institute_visual_functioning_question#% https://www.nei.nih.gov/sites/default/files/2019-06/vfq_sa.pdf %|National Eye Institute Visual Functioning Questionnaire - 25 (VFQ-25)||No|

	129	[vfq_5]	How much difficulty do you have reading ordinary print in newspapers? Would you say you have:	radio<br>1 | No difficulty at all<br>2 | A little difficulty<br>3 | Moderate difficulty<br>4 | Extreme difficulty<br>5 | Stopped doing this because of your eyesight<br>6 | Stopped doing this for other reasons or not interested in doing this<br><br>Custom alignment: LV<br>Field Annotation:<br>#national_eye_institute_visual_functioning_question#% https://www.nei.nih.gov/sites/default/files/2019-06/vfq_sa.pdf %|National Eye Institute Visual Functioning Questionnaire - 25 (VFQ-25)||No|
	130	[vfq_6]	How much difficulty do you have doing work or hobbies that require you to see well up close, such as cooking, sewing, fixing things around the house, or using hand tools? Would you say:	radio<br>1 | No difficulty at all<br>2 | A little difficulty<br>3 | Moderate difficulty<br>4 | Extreme difficulty<br>5 | Stopped doing this because of your eyesight<br>6 | Stopped doing this for other reasons or not interested in doing this<br><br>Custom alignment: LV<br>Field Annotation:<br>#national_eye_institute_visual_functioning_question#% https://www.nei.nih.gov/sites/default/files/2019-06/vfq_sa.pdf %|National Eye Institute Visual Functioning Questionnaire - 25 (VFQ-25)||No|
	131	[vfq_7]	Because of your eyesight, how much difficulty do you have finding something on a crowded shelf?	radio<br>1 | No difficulty at all<br>2 | A little difficulty<br>3 | Moderate difficulty<br>4 | Extreme difficulty<br>5 | Stopped doing this because of your eyesight<br>6 | Stopped doing this for other reasons or not interested in doing this<br><br>Custom alignment: LV<br>Field Annotation:<br>#national_eye_institute_visual_functioning_question#% https://www.nei.nih.gov/sites/default/files/2019-06/vfq_sa.pdf %|National Eye Institute Visual Functioning Questionnaire - 25 (VFQ-25)||No|
	132	[vfq_8]	How much difficulty do you have reading street signs or the names of stores?	radio<br>1 | No difficulty at all<br>2 | A little difficulty<br>3 | Moderate difficulty<br>4 | Extreme difficulty<br>5 | Stopped doing this because of your eyesight<br>6 | Stopped doing this for other reasons or not interested in doing this<br><br>Custom alignment: LV<br>Field Annotation:<br>#national_eye_institute_visual_functioning_question#% https://www.nei.nih.gov/sites/default/files/2019-06/vfq_sa.pdf %|National Eye Institute Visual Functioning Questionnaire - 25 (VFQ-25)||No|

	133	[vfq_9]	Because of your eyesight, how much difficulty do you have going down steps, stairs, or curbs in dim light or at night?	radio<br>1 No difficulty at all<br>2 A little difficulty<br>3 Moderate difficulty<br>4 Extreme difficulty<br>5 Stopped doing this because of your eyesight<br>6 Stopped doing this for other reasons or not interested in doing this<br><br>Custom alignment: LV<br>Field Annotation:<br>#national_eye_institute_visual_functioning_question#% https://www.nei.nih.gov/sites/default/files/2019-06/vfq_sa.pdf %|National Eye Institute Visual Functioning Questionnaire - 25 (VFQ-25)||No|
	134	[vfq_10]	Because of your eyesight, how much difficulty do you have noticing objects off to the side while you are walking along?	radio<br>1 No difficulty at all<br>2 A little difficulty<br>3 Moderate difficulty<br>4 Extreme difficulty<br>5 Stopped doing this because of your eyesight<br>6 Stopped doing this for other reasons or not interested in doing this<br><br>Custom alignment: LV<br>Field Annotation:<br>#national_eye_institute_visual_functioning_question#% https://www.nei.nih.gov/sites/default/files/2019-06/vfq_sa.pdf %|National Eye Institute Visual Functioning Questionnaire - 25 (VFQ-25)||No|
	135	[vfq_11]	Because of your eyesight, how much difficulty do you have seeing how people react to things you say?	radio<br>1 No difficulty at all<br>2 A little difficulty<br>3 Moderate difficulty<br>4 Extreme difficulty<br>5 Stopped doing this because of your eyesight<br>6 Stopped doing this for other reasons or not interested in doing this<br><br>Custom alignment: LV<br>Field Annotation:<br>#national_eye_institute_visual_functioning_question#% https://www.nei.nih.gov/sites/default/files/2019-06/vfq_sa.pdf %|National Eye Institute Visual Functioning Questionnaire - 25 (VFQ-25)||No|
	136	[vfq_12]	Because of your eyesight, how much difficulty do you have picking out and matching your own clothes?	radio<br>1 No difficulty at all<br>2 A little difficulty<br>3 Moderate difficulty<br>4 Extreme difficulty<br>5 Stopped doing this because of your eyesight<br>6 Stopped doing this for other reasons or not interested in doing this<br><br>Custom alignment: LV<br>Field Annotation:<br>#national_eye_institute_visual_functioning_question#% https://www.nei.nih.gov/sites/default/files/2019-06/vfq_sa.pdf %|National Eye Institute Visual Functioning Questionnaire - 25 (VFQ-25)||No|

	137	[vfq_13]	Because of your eyesight, how much difficulty do you have visiting with people in their homes, at parties, or in restaurants?	radio<br>1 No difficulty at all<br>2 A little difficulty<br>3 Moderate difficulty<br>4 Extreme difficulty<br>5 Stopped doing this because of your eyesight<br>6 Stopped doing this for other reasons or not interested in doing this<br><br>Custom alignment: LV<br>Field Annotation: #national_eye_institute_visual_functioning_question#% https://www.nei.nih.gov/sites/default/files/2019-06/vfq_sa.pdf %|National Eye Institute Visual Functioning Questionnaire - 25 (VFQ-25)||No|
	138	[vfq_14]	Because of your eyesight, how much difficulty do you have going out to see movies, plays, or sports events?	radio<br>1 No difficulty at all<br>2 A little difficulty<br>3 Moderate difficulty<br>4 Extreme difficulty<br>5 Stopped doing this because of your eyesight<br>6 Stopped doing this for other reasons or not interested in doing this<br><br>Custom alignment: LV<br>Field Annotation: #national_eye_institute_visual_functioning_question#% https://www.nei.nih.gov/sites/default/files/2019-06/vfq_sa.pdf %|National Eye Institute Visual Functioning Questionnaire - 25 (VFQ-25)||No|
	139	[vfq_15]	Are you currently driving, at least once in a while?	radio<br>1 Yes<br>2 No<br><br>Custom alignment: LV<br>Field Annotation: #national_eye_institute_visual_functioning_question#% https://www.nei.nih.gov/sites/default/files/2019-06/vfq_sa.pdf %|National Eye Institute Visual Functioning Questionnaire - 25 (VFQ-25)||No|
	140	[vfq_15a]	Have you never driven a car or have you given up driving?	radio<br>1 Never drove<br>2 Gave up<br><br>Custom alignment: LV<br>Field Annotation: #national_eye_institute_visual_functioning_question#% https://www.nei.nih.gov/sites/default/files/2019-06/vfq_sa.pdf %|National Eye Institute Visual Functioning Questionnaire - 25 (VFQ-25)||No|
	141	[vfq_15b]	Was that mainly because of your eyesight, mainly for some other reason, or because of both your eyesight and other reasons?	radio<br>1 Mainly eyesight<br>2 Mainly other reasons<br>3 Both eyesight and other reasons<br><br>Custom alignment: LV<br>Field Annotation: #national_eye_institute_visual_functioning_question#% https://www.nei.nih.gov/sites/default/files/2019-06/vfq_sa.pdf %|National Eye Institute Visual Functioning Questionnaire - 25 (VFQ-25)||No|

	142	[vfq_15c]	How much difficulty do you have driving during the daytime in familiar places? Would you say you have:	radio<br>1 No difficulty at all<br>2 A little difficulty<br>3 Moderate difficulty<br>4 Extreme difficulty<br><br>Custom alignment: LV<br>Field Annotation: #national_eye_institute_visual_functioning_question#% https://www.nei.nih.gov/sites/default/files/2019-06/vfq_sa.pdf %|National Eye Institute Visual Functioning Questionnaire - 25 (VFQ-25)||No|
	143	[vfq_16]	How much difficulty do you have driving at night? Would you say you have:	radio<br>1 No difficulty at all<br>2 A little difficulty<br>3 Moderate difficulty<br>4 Extreme difficulty<br>5 Have you stopped doing this because of your eyesight<br>6 Have you stopped doing this for other reasons or are you not interested in doing this<br><br>Custom alignment: LV<br>Field Annotation: #national_eye_institute_visual_functioning_question#% https://www.nei.nih.gov/sites/default/files/2019-06/vfq_sa.pdf %|National Eye Institute Visual Functioning Questionnaire - 25 (VFQ-25)||No|
	144	[vfq_16a]	How much difficulty do you have driving in difficult conditions, such as in bad weather, during rush hour, on the freeway, or in city traffic? Would you say you have:	radio<br>1 No difficulty at all<br>2 A little difficulty<br>3 Moderate difficulty<br>4 Extreme difficulty<br>5 Have you stopped doing this because of your eyesight<br>6 Have you stopped doing this for other reasons or are you not interested in doing this<br><br>Custom alignment: LV<br>Field Annotation: #national_eye_institute_visual_functioning_question#% https://www.nei.nih.gov/sites/default/files/2019-06/vfq_sa.pdf %|National Eye Institute Visual Functioning Questionnaire - 25 (VFQ-25)||No|
	145	[vfq_head1]	The next questions are about how things you do may be affected by your vision. For each one, please indicate whether for you the statement is true for you all, most, some, a little, or none of the time.	descriptive<br>Custom alignment: LV<br>Field Annotation: ||||
	146	[vfq_17]	Do you accomplish less than you would like because of your vision?	radio<br>1 All of the time<br>2 Most of the time<br>3 Some of the time<br>4 A little of the time<br>5 None of the time<br><br>Custom alignment: LV<br>Field Annotation: #national_eye_institute_visual_functioning_question#% https://www.nei.nih.gov/sites/default/files/2019-06/vfq_sa.pdf %|National Eye Institute Visual Functioning Questionnaire - 25 (VFQ-25)||No|

	147	[vfq_18]	Are you limited in how long you can work or do other activities because of your vision?	radio<br>1 All of the time<br>2 Most of the time<br>3 Some of the time<br>4 A little of the time<br>5 None of the time<br><br>Custom alignment: LV<br>Field Annotation: #national_eye_institute_visual_functioning_question#% https://www.nei.nih.gov/sites/default/files/2019-06/vfq_sa.pdf %|National Eye Institute Visual Functioning Questionnaire - 25 (VFQ-25)||No|
	148	[vfq_19]	How much does pain or discomfort in or around your eyes, for example, burning, itching, or aching, keep you from doing what you'd like to be doing? Would you say:	radio<br>1 All of the time<br>2 Most of the time<br>3 Some of the time<br>4 A little of the time<br>5 None of the time<br><br>Custom alignment: LV<br>Field Annotation: #national_eye_institute_visual_functioning_question#% https://www.nei.nih.gov/sites/default/files/2019-06/vfq_sa.pdf %|National Eye Institute Visual Functioning Questionnaire - 25 (VFQ-25)||No|
	149	[vfq_head2]	For each of the following statements, please indicate whether for you the statement is definitely true, mostly true, mostly false, or definitely false for you or you are not sure.	descriptive<br>Custom alignment: LV<br>Field Annotation: ||||
	150	[vfq_20]	I stay home most of the time because of my eyesight	radio<br>1 Definitely true<br>2 Mostly true<br>3 Not sure<br>4 Mostly false<br>5 Definitely false<br><br>Custom alignment: LV<br>Field Annotation: #national_eye_institute_visual_functioning_question#% https://www.nei.nih.gov/sites/default/files/2019-06/vfq_sa.pdf %|National Eye Institute Visual Functioning Questionnaire - 25 (VFQ-25)||No|
	151	[vfq_21]	I feel frustrated a lot of the time because of my eyesight	radio<br>1 Definitely true<br>2 Mostly true<br>3 Not sure<br>4 Mostly false<br>5 Definitely false<br><br>Custom alignment: LV<br>Field Annotation: #national_eye_institute_visual_functioning_question#% https://www.nei.nih.gov/sites/default/files/2019-06/vfq_sa.pdf %|National Eye Institute Visual Functioning Questionnaire - 25 (VFQ-25)||No|

	152	[vfq_22]	I have much less control over what I do, because of my eyesight.	radio<br>1 Definitely true<br>2 Mostly true<br>3 Not sure<br>4 Mostly false<br>5 Definitely false<br><br>Custom alignment: LV<br>Field Annotation: #national_eye_institute_visual_functioning_question#% https://www.nei.nih.gov/sites/default/files/2019-06/vfq_sa.pdf %|National Eye Institute Visual Functioning Questionnaire - 25 (VFQ-25)||No|
	153	[vfq_23]	Because of my eyesight, I have to rely too much on what other people tell me	radio<br>1 Definitely true<br>2 Mostly true<br>3 Not sure<br>4 Mostly false<br>5 Definitely false<br><br>Custom alignment: LV<br>Field Annotation: #national_eye_institute_visual_functioning_question#% https://www.nei.nih.gov/sites/default/files/2019-06/vfq_sa.pdf %|National Eye Institute Visual Functioning Questionnaire - 25 (VFQ-25)||No|
	154	[vfq_24]	I need a lot of help from others because of my eyesight	radio<br>1 Definitely true<br>2 Mostly true<br>3 Not sure<br>4 Mostly false<br>5 Definitely false<br><br>Custom alignment: LV<br>Field Annotation: #national_eye_institute_visual_functioning_question#% https://www.nei.nih.gov/sites/default/files/2019-06/vfq_sa.pdf %|National Eye Institute Visual Functioning Questionnaire - 25 (VFQ-25)||No|
	155	[vfq_25]	I worry about doing things that will embarrass myself or others, because of my eyesight	radio<br>1 Definitely true<br>2 Mostly true<br>3 Not sure<br>4 Mostly false<br>5 Definitely false<br><br>Custom alignment: LV<br>Field Annotation: #national_eye_institute_visual_functioning_question#% https://www.nei.nih.gov/sites/default/files/2019-06/vfq_sa.pdf %|National Eye Institute Visual Functioning Questionnaire - 25 (VFQ-25)||No|
	156	[compass31_faint_header]	Section Header:<br>This set of questions is about feeling faint, dizzy or goofy.	descriptive<br>Field Annotation: % COMPASS-31 %||||
	157	[compass31_faintfreq]	When standing up, how frequently do you get these feelings or symptoms?	radio<br>1 Rarely<br>2 Occasionally<br>3 Frequently<br>4 Almost always<br><br>Custom alignment: LV<br>Field Annotation: % COMPASS-31 %|Compass 31||No|

	158	[compass31_faintsev]	How would you rate the severity of these feelings or symptoms?	radio<br>1 Mild<br>2 Moderate<br>3 Severe<br><br>Custom alignment: LV<br>Field Annotation: % COMPASS-31 %|Compass 31||No|
	159	[compass31_fainttraj]	In the past year, have these feelings or symptoms that you have experienced:	radio<br>1 Gotten much worse<br>2 Gotten somewhat worse<br>3 Stayed about the same<br>4 Gotten somewhat better<br>5 Gotten much better<br>6 Completely gone<br><br>Custom alignment: LV<br>Field Annotation: % COMPASS-31 %|Compass 31||No|
	160	[compass31_color_header]	Section Header:<br>This set of questions is about changes in skin color.	descriptive<br>Field Annotation: % COMPASS-31 %||||
	161	[compass31_colorloc]	What parts of your body are affected by these color changes? (check all that apply)	checkbox<br>1 compass31_colorloc___1 Hands<br>2 compass31_colorloc___2 Feet<br><br>Custom alignment: LV<br>Field Annotation: % COMPASS-31 %|Compass 31||No|
	162	[compass31_colortraj]	Are these changes in your skin color:	radio<br>1 Getting much worse<br>2 Getting somewhat worse<br>3 Staying about the same<br>4 Getting somewhat better<br>5 Getting much better<br>6 Completely gone<br><br>Custom alignment: LV<br>Field Annotation: % COMPASS-31 %|Compass 31||No|
	163	[compass31_sweatyn]	In the past 5 years, what changes, if any, have occurred in your general body sweating?	radio<br>1 I sweat much more than I used to<br>2 I sweat somewhat more than I used to<br>3 I haven't noticed any changes in my sweating<br>4 I sweat somewhat less than I used to<br>5 I sweat much less than I used to<br><br>Custom alignment: LV<br>Field Annotation: % COMPASS-31 %|Compass 31||No|
	164	[compass31_dryeyesyn]	Do your eyes feel excessively dry?	radio<br>1 Yes<br>0 No<br><br>Custom alignment: LV<br>Field Annotation: % COMPASS-31 %|Compass 31||Yes|numbering
	165	[compass31_drymouth_header]	Section Header:<br>This set of questions is about having an excessively dry mouth.	descriptive<br>Field Annotation: % COMPASS-31 %||||

	166	[compass31_drymouthtraj]	For the symptom of dry mouth that you had had for the longest period of time, is this symptom:	radio<br>1 I have not had any of these symptoms<br>2 Getting much worse<br>3 Getting somewhat worse<br>4 Staying about the same<br>5 Getting somewhat better<br>6 Getting much better<br>7 Completely gone<br><br>Custom alignment: LV<br>Field Annotation: % COMPASS-31 %|Compass 31||No|
	167	[compass31_gastro_header]	Section Header:<br>This set of questions is about belly problems.	descriptive<br>Field Annotation: % COMPASS-31 %||||
	168	[compass31_fullrate]	In the past year, have you noticed any changes in how quickly you get full when eating a meal?	radio<br>1 I get full a lot more quickly than I used to<br>2 I get full more quickly than I used to<br>3 I haven't noticed any change<br>4 I get full less quickly than I used to<br>5 I get full a lot less quickly than I used to<br><br>Custom alignment: LV<br>Field Annotation: % COMPASS-31 %|Compass 31||No|
	169	[compass31_bloated]	In the past year, have you felt excessively full or persistently full (bloated feeling) after a meal?	radio<br>1 Never<br>2 Sometimes<br>3 A lot of the time<br><br>Custom alignment: LV<br>Field Annotation: % COMPASS-31 %|Compass 31||No|
	170	[compass31_vomit]	In the past year, have you ever vomited after a meal?	radio<br>1 Never<br>2 Sometimes<br>3 A lot of the time<br><br>Custom alignment: LV<br>Field Annotation: % COMPASS-31 %|Compass 31||No|
	171	[compass31_cramp]	In the past year, have you had a cramping or colicky abdominal pain?	radio<br>1 Never<br>2 Sometimes<br>3 A lot of the time<br><br>Custom alignment: LV<br>Field Annotation: % COMPASS-31 %|Compass 31||No|
	172	[compass31_diarryn]	In the past year, have you had any bouts of diarrhea?	radio<br>1 Yes<br>0 No<br><br>Custom alignment: LV<br>Field Annotation: % COMPASS-31 %|Compass 31||No|
	173	[compass31_diarrfreq]	How frequently does this occur?	radio<br>1 Rarely<br>2 Occasionally<br>3 Frequently<br>4 Constantly<br><br>Custom alignment: LV<br>Field Annotation: % COMPASS-31 %|Compass 31||No|

	174	[compass31_diarrsev]	How severe are these bouts of diarrhea?	radio<br>1 Mild<br>2 Moderate<br>3 Severe<br><br>Custom alignment: LV<br>Field Annotation: % COMPASS-31 %|Compass 31||No|
	175	[compass31_diarrtraj]	Are your bouts of diarrhea getting:	radio<br>1 Much worse<br>2 Somewhat worse<br>3 Staying the same<br>4 Somewhat better<br>5 Much better<br>6 Completely gone<br><br>Custom alignment: LV<br>Field Annotation: % COMPASS-31 %|Compass 31||No|
	176	[compass31_constyn]	In the past year, have you been constipated?	radio<br>1 Yes<br>0 No<br><br>Custom alignment: LV<br>Field Annotation: % COMPASS-31 %|Compass 31||No|
	177	[compass31_constfreq]	How frequently are you constipated?	radio<br>1 Rarely<br>2 Occasionally<br>3 Frequently<br>4 Constantly<br><br>Custom alignment: LV<br>Field Annotation: % COMPASS-31 %|Compass 31||No|
	178	[compass31_constsev]	How severe are these episodes of constipation?	radio<br>1 Mild<br>2 Moderate<br>3 Severe<br><br>Custom alignment: LV<br>Field Annotation: % COMPASS-31 %|Compass 31||No|
	179	[compass31_consttraj]	Is your constipation getting:	radio<br>1 Much worse<br>2 Somewhat worse<br>3 Staying the same<br>4 Somewhat better<br>5 Much better<br>6 Completely gone<br><br>Custom alignment: LV<br>Field Annotation: % COMPASS-31 %|Compass 31||No|
	180	[compass31_bladder_header]	Section Header:<br>This set of questions is about bladder problems.	descriptive<br>Field Annotation: % COMPASS-31 %||||
	181	[compass31_controlbladder]	In the past year, have you ever lost control of your bladder function?	radio<br>1 Never<br>2 Occasionally<br>3 Frequently<br>4 Constantly<br><br>Custom alignment: LV<br>Field Annotation: % COMPASS-31 %|Compass 31||No|

	182	[compass31_urinepass]	In the past year, have you had difficulty passing urine?	radio<br>1 Never<br>2 Occasionally<br>3 Frequently<br>4 Constantly<br><br>Custom alignment: LV<br>Field Annotation: % COMPASS-31 %|Compass 31||No|
	183	[compass31_emptybladder]	In the past year, have you had trouble completely emptying your bladder?	radio<br>1 Never<br>2 Occasionally<br>3 Frequently<br>4 Constantly<br><br>Custom alignment: LV<br>Field Annotation: % COMPASS-31 %|Compass 31||No|
	184	[compass31_vision_header]	Section Header:<br>This set of questions is about vision problems.	descriptive<br>Field Annotation: % COMPASS-31 %||||
	185	[compass31_lightyn]	In the past year, without sunglasses or tinted glasses, has bright light bothered your eyes?	radio<br>1 Never<br>2 Occasionally<br>3 Frequently<br>4 Constantly<br><br>Custom alignment: LV<br>Field Annotation: % COMPASS-31 %|Compass 31||No|
	186	[compass31_lightsev]	How severe is this sensitivity to bright light?	radio<br>1 Mild<br>2 Moderate<br>3 Severe<br><br>Custom alignment: LV<br>Field Annotation: % COMPASS-31 %|Compass 31||No|
	187	[compass31_focusyn]	In the past year, have you had trouble focusing your eyes?	radio<br>1 Never<br>2 Occasionally<br>3 Frequently<br>4 Constantly<br><br>Custom alignment: LV<br>Field Annotation: % COMPASS-31 %|Compass 31||No|
	188	[compass31_vistraj]	Is the most troublesome symptom with your eyes (ie, sensitivity to bright light or trouble focusing) getting:	radio<br>1 I have not had any of these symptoms<br>2 Much worse<br>3 Somewhat worse<br>4 Staying about the same<br>5 Somewhat better<br>6 Much better<br>7 Completely gone<br><br>Custom alignment: LV<br>Field Annotation: % COMPASS-31 %|Compass 31||No|
	189	[compass31_focussev]	How severe is this focusing problem?	radio<br>1 Mild<br>2 Moderate<br>3 Severe<br><br>Custom alignment: LV<br>Field Annotation: % COMPASS-31 %|Compass 31||No|

	190	[pasc_symptoms_complete]	Section Header: *Form Status*<br>Complete?	dropdown<br>0 Incomplete<br>1 Unverified<br>2 Complete
Instrument: **Pcl5** (pcl5)				
	191	[pcl5_intro]	Section Header:<br>Below is a list of problems that people sometimes have in response to a very stressful experience. Please read each problem carefully and then select the option that indicates how much you have been bothered by that problem in the past month	descriptive<br>Field Annotation: ||||
	192	[pcl5_intro2]	In the past month, how much were you bothered by:	descriptive<br>Field Annotation: ||||
	193	[pcl5_01]	Repeated, disturbing, and unwanted memories of the stressful experience?	radio (Matrix)<br>0 Not at all<br>1 A little bit<br>2 Moderately<br>3 Quite a bit<br>4 Extremely<br>-88 I prefer not to answer<br><br>Field Annotation: % PCL-5 https://www.ptsd.va.gov/professional/assessment/adult-sr/ptsd-checklist.asp %|PTSD Checklist for DSM-5 (PCL-5)||Yes|-88, I prefer not to answer
	194	[pcl5_02]	Repeated, disturbing dreams of the stressful experience?	radio (Matrix)<br>0 Not at all<br>1 A little bit<br>2 Moderately<br>3 Quite a bit<br>4 Extremely<br>-88 I prefer not to answer<br><br>Field Annotation: % PCL-5 https://www.ptsd.va.gov/professional/assessment/adult-sr/ptsd-checklist.asp %|PTSD Checklist for DSM-5 (PCL-5)||Yes|-88, I prefer not to answer
	195	[pcl5_03]	Suddenly feeling or acting as if the stressful experience were actually happening again (as if you were actually back there reliving it)?	radio (Matrix)<br>0 Not at all<br>1 A little bit<br>2 Moderately<br>3 Quite a bit<br>4 Extremely<br>-88 I prefer not to answer<br><br>Field Annotation: % PCL-5 https://www.ptsd.va.gov/professional/assessment/adult-sr/ptsd-checklist.asp %|PTSD Checklist for DSM-5 (PCL-5)||Yes|-88, I prefer not to answer

	196	[pcl5_04]	Feeling very upset when something reminded you of the stressful experience?	radio (Matrix)<br>0 Not at all<br>1 A little bit<br>2 Moderately<br>3 Quite a bit<br>4 Extremely<br>-88 I prefer not to answer<br><br>Field Annotation: % PCL-5 https://www.ptsd.va.gov/professional/assessment/adult-sr/ptsd-checklist.asp %|PTSD Checklist for DSM-5 (PCL-5)||Yes|-88, I prefer not to answer
	197	[pcl5_05]	Having strong physical reactions when something reminded you of the stressful experience (for example, heart pounding, trouble breathing, sweating)?	radio (Matrix)<br>0 Not at all<br>1 A little bit<br>2 Moderately<br>3 Quite a bit<br>4 Extremely<br>-88 I prefer not to answer<br><br>Field Annotation: % PCL-5 https://www.ptsd.va.gov/professional/assessment/adult-sr/ptsd-checklist.asp %|PTSD Checklist for DSM-5 (PCL-5)||Yes|-88, I prefer not to answer
	198	[pcl5_06]	Avoiding memories, thoughts, or feelings related to the stressful experience?	radio (Matrix)<br>0 Not at all<br>1 A little bit<br>2 Moderately<br>3 Quite a bit<br>4 Extremely<br>-88 I prefer not to answer<br><br>Field Annotation: % PCL-5 https://www.ptsd.va.gov/professional/assessment/adult-sr/ptsd-checklist.asp %|PTSD Checklist for DSM-5 (PCL-5)||Yes|-88, I prefer not to answer
	199	[pcl5_07]	Avoiding external reminders of the stressful experience (for example, people, places, conversations, activities, objects, or situations)?	radio (Matrix)<br>0 Not at all<br>1 A little bit<br>2 Moderately<br>3 Quite a bit<br>4 Extremely<br>-88 I prefer not to answer<br><br>Field Annotation: % PCL-5 https://www.ptsd.va.gov/professional/assessment/adult-sr/ptsd-checklist.asp %|PTSD Checklist for DSM-5 (PCL-5)||Yes|-88, I prefer not to answer
	200	[pcl5_08]	Trouble remembering important parts of the stressful experience?	radio (Matrix)<br>0 Not at all<br>1 A little bit<br>2 Moderately<br>3 Quite a bit<br>4 Extremely<br>-88 I prefer not to answer<br><br>Field Annotation: % PCL-5 https://www.ptsd.va.gov/professional/assessment/adult-sr/ptsd-checklist.asp %|PTSD Checklist for DSM-5 (PCL-5)||Yes|-88, I prefer not to answer

	201	[pcl5_09]	Having strong negative beliefs about yourself, other people, or the world (for example, having thoughts such as: I am bad, there is something seriously wrong with me, no one can be trusted, the world is completely dangerous)?	radio (Matrix)<br>0 Not at all<br>1 A little bit<br>2 Moderately<br>3 Quite a bit<br>4 Extremely<br>-88 I prefer not to answer<br><br>Field Annotation: % PCL-5 https://www.ptsd.va.gov/professional/assessment/adult-sr/ptsd-checklist.asp %|PTSD Checklist for DSM-5 (PCL-5)||Yes|-88, I prefer not to answer
	202	[pcl5_10]	Blaming yourself or someone else for the stressful experience or what happened after it?	radio (Matrix)<br>0 Not at all<br>1 A little bit<br>2 Moderately<br>3 Quite a bit<br>4 Extremely<br>-88 I prefer not to answer<br><br>Field Annotation: % PCL-5 https://www.ptsd.va.gov/professional/assessment/adult-sr/ptsd-checklist.asp %|PTSD Checklist for DSM-5 (PCL-5)||Yes|-88, I prefer not to answer
	203	[pcl5_11]	Having strong negative feelings such as fear, horror, anger, guilt, or shame?	radio (Matrix)<br>0 Not at all<br>1 A little bit<br>2 Moderately<br>3 Quite a bit<br>4 Extremely<br>-88 I prefer not to answer<br><br>Field Annotation: % PCL-5 https://www.ptsd.va.gov/professional/assessment/adult-sr/ptsd-checklist.asp %|PTSD Checklist for DSM-5 (PCL-5)||Yes|-88, I prefer not to answer
	204	[pcl5_12]	Loss of interest in activities that you used to enjoy?	radio (Matrix)<br>0 Not at all<br>1 A little bit<br>2 Moderately<br>3 Quite a bit<br>4 Extremely<br>-88 I prefer not to answer<br><br>Field Annotation: % PCL-5 https://www.ptsd.va.gov/professional/assessment/adult-sr/ptsd-checklist.asp %|PTSD Checklist for DSM-5 (PCL-5)||Yes|-88, I prefer not to answer
	205	[pcl5_13]	Feeling distant or cut off from other people?	radio (Matrix)<br>0 Not at all<br>1 A little bit<br>2 Moderately<br>3 Quite a bit<br>4 Extremely<br>-88 I prefer not to answer<br><br>Field Annotation: % PCL-5 https://www.ptsd.va.gov/professional/assessment/adult-sr/ptsd-checklist.asp %|PTSD Checklist for DSM-5 (PCL-5)||Yes|-88, I prefer not to answer

	206	[pcl5_14]	Trouble experiencing positive feelings (for example, being unable to feel happiness or have loving feelings for people close to you)?	radio (Matrix)<br>0 Not at all<br>1 A little bit<br>2 Moderately<br>3 Quite a bit<br>4 Extremely<br>-88 I prefer not to answer<br><br>Field Annotation: % PCL-5 https://www.ptsd.va.gov/professional/assessment/adult-sr/ptsd-checklist.asp %|PTSD Checklist for DSM-5 (PCL-5)||Yes|-88, I prefer not to answer
	207	[pcl5_15]	Irritable behavior, angry outbursts, or acting aggressively?	radio (Matrix)<br>0 Not at all<br>1 A little bit<br>2 Moderately<br>3 Quite a bit<br>4 Extremely<br>-88 I prefer not to answer<br><br>Field Annotation: % PCL-5 https://www.ptsd.va.gov/professional/assessment/adult-sr/ptsd-checklist.asp %|PTSD Checklist for DSM-5 (PCL-5)||Yes|-88, I prefer not to answer
	208	[pcl5_16]	Taking too many risks or doing things that could cause you harm?	radio (Matrix)<br>0 Not at all<br>1 A little bit<br>2 Moderately<br>3 Quite a bit<br>4 Extremely<br>-88 I prefer not to answer<br><br>Field Annotation: % PCL-5 https://www.ptsd.va.gov/professional/assessment/adult-sr/ptsd-checklist.asp %|PTSD Checklist for DSM-5 (PCL-5)||Yes|-88, I prefer not to answer
	209	[pcl5_17]	Being "superalert" or watchful or on guard?	radio (Matrix)<br>0 Not at all<br>1 A little bit<br>2 Moderately<br>3 Quite a bit<br>4 Extremely<br>-88 I prefer not to answer<br><br>Field Annotation: % PCL-5 https://www.ptsd.va.gov/professional/assessment/adult-sr/ptsd-checklist.asp %|PTSD Checklist for DSM-5 (PCL-5)||Yes|-88, I prefer not to answer
	210	[pcl5_18]	Feeling jumpy or easily startled?	radio (Matrix)<br>0 Not at all<br>1 A little bit<br>2 Moderately<br>3 Quite a bit<br>4 Extremely<br>-88 I prefer not to answer<br><br>Field Annotation: % PCL-5 https://www.ptsd.va.gov/professional/assessment/adult-sr/ptsd-checklist.asp %|PTSD Checklist for DSM-5 (PCL-5)||Yes|-88, I prefer not to answer

	211	[pcl5_19]	Having difficulty concentrating?	radio (Matrix)<br>0 Not at all<br>1 A little bit<br>2 Moderately<br>3 Quite a bit<br>4 Extremely<br>-88 I prefer not to answer<br><br>Field Annotation: % PCL-5 https://www.ptsd.va.gov/professional/assessment/adult-sr/ptsd-checklist.asp %|PTSD Checklist for DSM-5 (PCL-5)||Yes|-88, I prefer not to answer
	212	[pcl5_20]	Trouble falling or staying asleep?	radio (Matrix)<br>0 Not at all<br>1 A little bit<br>2 Moderately<br>3 Quite a bit<br>4 Extremely<br>-88 I prefer not to answer<br><br>Field Annotation: % PCL-5 https://www.ptsd.va.gov/professional/assessment/adult-sr/ptsd-checklist.asp %|PTSD Checklist for DSM-5 (PCL-5)||Yes|-88, I prefer not to answer
	213	[pcl5_complete]	Section Header: *Form Status*<br>Complete?	dropdown<br>0 Incomplete<br>1 Unverified<br>2 Complete
Instrument: **Home Polysomnography With Ess And Isi** (home_polysomnography_with_ess_and_isi)				
	214	[epworth_intro]	Section Header:<br>How likely are you to doze off or fall asleep in the following situations, in contrast to feeling just tired?This refers to your usual way of life in recent times.Even if you haven't done some of these things recently, try to work out how they would have affected you.	descriptive<br>Field Annotation: % Epworth Sleepiness Scale https://epworthsleepinessscale.com/about-the-ess/ %|The Epworth Sleepiness Scale (ESS)|||
	215	[epworth_sit_read]	Sitting and reading	radio (Matrix)<br>1 No chance of dozing<br>2 Slight chance of dozing<br>3 Moderate chance of dozing<br>4 High chance of dozing<br><br>Field Annotation: % Epworth Sleepiness Scale https://epworthsleepinessscale.com/about-the-ess/ %|The Epworth Sleepiness Scale (ESS)|||
	216	[epworth_tv]	Watching TV	radio (Matrix)<br>1 No chance of dozing<br>2 Slight chance of dozing<br>3 Moderate chance of dozing<br>4 High chance of dozing<br><br>Field Annotation: % Epworth Sleepiness Scale https://epworthsleepinessscale.com/about-the-ess/ %|The Epworth Sleepiness Scale (ESS)|||

	217	[epworth_sit_public]	Sitting, inactive in a public place (e.g. a theatre or a meeting)	radio (Matrix)<br>1 No chance of dozing<br>2 Slight chance of dozing<br>3 Moderate chance of dozing<br>4 High chance of dozing<br><br>Field Annotation: % Epworth Sleepiness Scale https://epworthsleepinessscale.com/about-the-ess/ %|The Epworth Sleepiness Scale (ESS)|||
	218	[epworth_passenger]	As a passenger in a car for an hour without a break	radio (Matrix)<br>1 No chance of dozing<br>2 Slight chance of dozing<br>3 Moderate chance of dozing<br>4 High chance of dozing<br><br>Field Annotation: % Epworth Sleepiness Scale https://epworthsleepinessscale.com/about-the-ess/ %|The Epworth Sleepiness Scale (ESS)|||
	219	[epworth_lying_down]	Lying down to rest in the afternoon when circumstances permit	radio (Matrix)<br>1 No chance of dozing<br>2 Slight chance of dozing<br>3 Moderate chance of dozing<br>4 High chance of dozing<br><br>Field Annotation: % Epworth Sleepiness Scale https://epworthsleepinessscale.com/about-the-ess/ %|The Epworth Sleepiness Scale (ESS)|||
	220	[epworth_sit_talk]	Sitting and talking to someone	radio (Matrix)<br>1 No chance of dozing<br>2 Slight chance of dozing<br>3 Moderate chance of dozing<br>4 High chance of dozing<br><br>Field Annotation: % Epworth Sleepiness Scale https://epworthsleepinessscale.com/about-the-ess/ %|The Epworth Sleepiness Scale (ESS)|||
	221	[epworth_sit_lunch]	Sitting quietly after a lunch without alcohol	radio (Matrix)<br>1 No chance of dozing<br>2 Slight chance of dozing<br>3 Moderate chance of dozing<br>4 High chance of dozing<br><br>Field Annotation: % Epworth Sleepiness Scale https://epworthsleepinessscale.com/about-the-ess/ %|The Epworth Sleepiness Scale (ESS)|||
	222	[epworth_traffic]	In a car, while stopped for a few minutes in the traffic	radio (Matrix)<br>1 No chance of dozing<br>2 Slight chance of dozing<br>3 Moderate chance of dozing<br>4 High chance of dozing<br><br>Field Annotation: % Epworth Sleepiness Scale https://epworthsleepinessscale.com/about-the-ess/ %|The Epworth Sleepiness Scale (ESS)|||

	223	[isi_fall]	Section Header: *For each question, please indicate the severity of your insomnia problem, if any*<br>Difficulty falling asleep	radio (Matrix)<br>1 None<br>2 Mild<br>3 Moderate<br>4 Severe<br>5 Very Severe<br><br>Field Annotation: % Insomnia Severity Index https://www.ons.org/sites/default/files/InsomniaSeverityInde %|Insomnia Severity Index||Yes|numbering
	224	[isi_stay]	Difficulty staying asleep	radio (Matrix)<br>1 None<br>2 Mild<br>3 Moderate<br>4 Severe<br>5 Very Severe<br><br>Field Annotation: % Insomnia Severity Index https://www.ons.org/sites/default/files/InsomniaSeverityInde %|Insomnia Severity Index||Yes|numbering
	225	[isi_wake]	Problems waking up too early	radio (Matrix)<br>1 None<br>2 Mild<br>3 Moderate<br>4 Severe<br>5 Very Severe<br><br>Field Annotation: % Insomnia Severity Index https://www.ons.org/sites/default/files/InsomniaSeverityInde %|Insomnia Severity Index||Yes|numbering
	226	[isi_sat]	How satisfied/dissatisfied are you with your current sleep pattern?	radio<br>0 Very satisfied<br>1 Satisfied<br>2 Moderately satisfied<br>3 Dissatisfied<br>4 Very dissatisfied<br><br>Custom alignment: LV<br>Field Annotation: % Insomnia Severity Index https://www.ons.org/sites/default/files/InsomniaSeverityInde %|Insomnia Severity Index||No|
	227	[isi_notice]	How noticeable to others do you think your sleep problem is in terms of impairing the quality of your life?	radio<br>0 Not at all noticeable<br>1 A little noticeable<br>2 Somewhat noticeable<br>3 Much noticeable<br>4 Very much noticeable<br><br>Custom alignment: LV<br>Field Annotation: % Insomnia Severity Index https://www.ons.org/sites/default/files/InsomniaSeverityInde %|Insomnia Severity Index||No|

	228	[isi_worried]	How worried/distressed are you about your current sleep problem?	radio<br>0 Not at all worried<br>1 A little worried<br>2 Somewhat worried<br>3 Much worried<br>4 Very much worried<br><br>Custom alignment: LV<br>Field Annotation: % Insomnia Severity Index https://www.ons.org/sites/default/files/InsomniaSeverityInde<br>%|Insomnia Severity Index||No|
	229	[isi_interfere]	To what extent do you consider your sleep problem to interfere with your daily functioning (e.g. daytime fatigue, mood, ability to function at work/daily chores, concentration, memory, mood, etc.) currently?	radio<br>0 Not at all interfering<br>1 A little interfering<br>2 Somewhat interfering<br>3 Much interfering<br>4 Very much interfering<br><br>Custom alignment: LV<br>Field Annotation: % Insomnia Severity Index https://www.ons.org/sites/default/files/InsomniaSeverityInde<br>%|Insomnia Severity Index||No|
	230	[home_polysomnography_with_ess_and_isi_complete]	Section Header: *Form Status*<br>Complete?	dropdown<br>0 Incomplete<br>1 Unverified<br>2 Complete
