# Supplementary material for: A community-engaged approach to developing common data elements: a case study from the RADx-UP Long COVID common data elements Task Force
Source: JAMIA Open. 2025 Jun 4;8(3):ooaf046. doi: 10.1093/jamiaopen/ooaf046 (PMC12136053; doi:10.1093/jamiaopen/ooaf046)
Supplement: ooaf046_Supplementary_Data [file ooaf046_supplementary_data.zip › Supplementary CDEs 2_RADxUP_1.7_Legacy_Tier1_Tier2_forms.pdf]

Consent

Record ID	__________________________________

Consent

Is consent required for this study?	◯ Yes, consent is required for this study<br>◯ No, Consent is not required/is waived for this study
Date of Consent	__________________________________<br>(MM/DD/YYYY)
I agree to let the Duke Clinical Research Institute collect the following identifiable information: name, address, contact information, and date of birth.	◯ Yes ◯ No<br>(This is to enable linkage of deidentified data.)
I agree to let the Duke Clinical Research Institute collect my zip code.	◯ Yes ◯ No
I agree to be contacted for future research.	◯ Yes ◯ No

Location

County

____

Zip Code

____

Sociodemographics

Date of Sociodemographic Data Collection

____
(MM/DD/YYYY)

Demographics

What is your race?

Mark one or more boxes.

☐ American Indian or Alaska Native
☐ Black or African American
☐ Asian
☐ Native Hawaiian or Other Pacific Islander
☐ White
☐ Some other race
☐ Prefer not to answer
(Check all that apply)

☐ Asian Indian
☐ Bangladeshi
☐ Bhutanese
☐ Burmese
☐ Cambodian
☐ Chinese, except Taiwanese
☐ Filipino
☐ Hmong
☐ Indonesian
☐ Japanese
☐ Korean
☐ Laotian
☐ Malaysian
☐ Mongolian
☐ Nepalese
☐ Okinawan
☐ Pakistani
☐ Sri Lankan
☐ Taiwanese
☐ Thai
☐ Vietnamese
☐ Other Asian
☐ Prefer not to answer
(Check all that apply)

☐ Native Hawaiian
☐ Pacific Islander
☐ Samoan
☐ Tongan
☐ Maori
☐ Fijian
☐ Chamorro
☐ Chuukese
☐ Kosraen
☐ Marshallese
☐ Palauan
☐ Pohnpeian
☐ Yapese
☐ Other Pacific Islander
☐ Prefer not to answer
(Check all that apply)

Specify other origin.	__________________________________
Are you of Hispanic, Latino, or Spanish origin?	◯ No, not of Hispanic, Latino, or Spanish origin<br>◯ Yes, of Hispanic, Latino, or Spanish origin<br>◯ Prefer not to answer
Please specify your origin	☐ Mexican, Mexican Am., Chicano<br>☐ Puerto Rican<br>☐ Cuban<br>☐ Another Hispanic, Latino, or Spanish origin<br>☐ Salvadoran<br>☐ Dominican<br>☐ Colombian<br>☐ Prefer not to answer
Please specify other Hispanic, Latino, or Spanish origin. For example, Salvadoran, Dominican, Colombian, Guatemalan, Spaniard, Ecuadorian, etc.	__________________________________
Age<br><br>For babies less than 1 year old, do not write the age in months. Write 0 as the age.	__________________________________<br>(Years)
What was your sex assigned at birth on your birth certificate?	◯ Female<br>◯ Male<br>◯ Intersex<br>◯ None of these describe me<br>◯ Prefer not to answer
What terms best express how you describe your gender identity?	◯ Woman<br>◯ Man<br>◯ Transgender man/Female-to-male (FTM)<br>◯ Transgender woman/Male-to-female (MTF)<br>◯ Gender non-binary/Genderqueer/Gender nonconforming<br>◯ Agender<br>◯ Bigender<br>◯ Two-spirit<br>◯ None of these describe me<br>◯ Prefer not to answer
Are you currently pregnant?	◯ Pregnant<br>◯ Not Pregnant<br>◯ Don't know<br>◯ Prefer not to answer
Which of the following best represents how you think of yourself at this time?	◯ Gay<br>◯ Lesbian<br>◯ Straight; that is, not gay or lesbian, etc.<br>◯ Bisexual<br>◯ None of these describe me<br>◯ Prefer not to answer

What is the highest level of education you have achieved outside or in the United States? Grades roughly equivalent to years of school.

- ◯ Have never gone to school
- ◯ 5th grade or less
- ◯ 6th to 8th grade
- ◯ 9th to 12th grade, no diploma
- ◯ High school graduate or GED completed
- ◯ Some college level/ Technical / Vocational degree
- ◯ Bachelor's degree
- ◯ Other advanced degree (Master's, Doctoral degree)
- ◯ Prefer not to answer
- ◯ Don't know

Housing Employment And Insurance

Housing

Date of Housing, Employment and Insurance Collection

____
(MM/DD/YYYY)

What best describes the people at your home:

- ◯ Just me
- ◯ Living with spouse, no kids
- ◯ Family including kids
- ◯ Family with 3 generations (parents, children, grandchildren)
- ◯ Family with 4 generations
- ◯ Living with roommates
- ◯ None of these
- ◯ Prefer not to answer

Are you currently living in transitional housing, staying in a shelter, or experiencing homelessness?

- ◯ Yes
- ◯ No
- ◯ Prefer not to answer
- ◯ Don't know

Do you live in any of these?

- ◯ A group care setting
- ◯ Nursing home
- ◯ Residential care facility for people with intellectual and developmental disabilities
- ◯ A psychiatric treatment facility
- ◯ A group home
- ◯ A board and care home
- ◯ Prison or jail
- ◯ A halfway house
- ◯ Foster care
- ◯ Homeless or in no consistent shelter
- ◯ Somewhere else
- ◯ Prefer not to answer

Where do you stay/live?

____

Employment

Have you, or has anyone in your household, experienced a loss of employment income since the start of the COVID-19 pandemic (March 2020)?

- ◯ Yes
- ◯ No
- ◯ Prefer not to answer

We would like to know about what you do -- are you working now, looking for work, retired, keeping house, a student, or something else?

- ◯ Working now
- ◯ Only temporarily laid off, sick leave or maternity leave
- ◯ Looking for work, unemployed
- ◯ Retired
- ◯ Disabled, permanently or temporarily
- ◯ Keeping house
- ◯ Student
- ◯ Other (Specify)
- ◯ Prefer not to answer
- ◯ Don't know

Current employment status, Other - specify	__________________________
Are you considered an essential worker? An essential worker is someone who was required to go to work even when stay at home orders were in place	◯ Yes<br>◯ No<br>◯ Prefer not to answer<br>◯ Don't know
Would any of these describe where you work? If you work multiple jobs, select the closest match to your main job.	◯ Nursing care facilities<br>◯ Visiting nurse or home health aide service<br>◯ Building cleaning services<br>◯ Public transportation<br>◯ Corrections facility<br>◯ EMT or paramedic services<br>◯ Meat packing farm facility<br>◯ Agriculture and food production facility<br>◯ Grocery store<br>◯ Construction<br>◯ No<br>◯ Prefer not to answer
What is the primary kind of health insurance or health care plan that you have now?	◯ I do NOT have health insurance<br>◯ Private (purchased directly or through Employment)<br>◯ Public (Medicare, Medicaid, Tricare)<br>◯ Don't know<br>◯ Prefer not to answer<br>(Exclude plans that pay for only one type of Service (such as, nursing home care, accidents, family planning, or dental care) and plans that only provide extra cash when hospitalized.<br>)
Did you lose health coverage because of the COVID-19 pandemic?	◯ Yes<br>◯ No<br>◯ Prefer not to answer<br>◯ Don't know
The COVID-19 pandemic may cause challenges for some people, whether they get COVID-19 or not. In the past 6 months have you or your family experienced any of the below challenges?	
Getting the health care I need (including for mental health)	◯ No, not a challenge<br>◯ Yes, a minor challenge<br>◯ Yes, this is a major challenge<br>◯ Prefer not to answer
Having a place to stay/live	◯ No, not a challenge<br>◯ Yes, a minor challenge<br>◯ Yes, this is a major challenge<br>◯ Prefer not to answer
Getting enough food to eat	◯ No, not a challenge<br>◯ Yes, a minor challenge<br>◯ Yes, this is a major challenge<br>◯ Prefer not to answer
Having clean water to drink	◯ No, not a challenge<br>◯ Yes, a minor challenge<br>◯ Yes, this is a major challenge<br>◯ Prefer not to answer

Getting the medicine I need	◯ No, not a challenge<br>◯ Yes, a minor challenge<br>◯ Yes, this is a major challenge<br>◯ Prefer not to answer
Getting to where I need to go	◯ No, not a challenge<br>◯ Yes, a minor challenge<br>◯ Yes, this is a major challenge<br>◯ Prefer not to answer

Spoken Language

What languages do you read, understand, or speak at home?

- ☐ English
- ☐ Spanish
- ☐ Albanian
- ☐ Apache
- ☐ Arabic
- ☐ Bengali/Bangla
- ☐ Bhutanese
- ☐ Burmese
- ☐ Cantonese
- ☐ Cape Verdean Creole
- ☐ Creole
- ☐ Chamoru
- ☐ Chuukese
- ☐ Dakota
- ☐ Fijian
- ☐ French
- ☐ Hawaiian
- ☐ Hmong
- ☐ Ilokano
- ☐ Karen
- ☐ Khmer/Cambodian
- ☐ Kinyarwanda
- ☐ Korean
- ☐ Kosraean
- ☐ Lakota
- ☐ Lingala
- ☐ Mam
- ☐ Mandarin
- ☐ Marshallese
- ☐ Mixteco
- ☐ Nakota
- ☐ Navajo
- ☐ Nepali
- ☐ Portuguese
- ☐ Pohnpeian
- ☐ Russian
- ☐ Sign Language
- ☐ Somali
- ☐ Samoan
- ☐ Swahili
- ☐ Tagalog
- ☐ Thai
- ☐ Tongan
- ☐ Triqui
- ☐ Vietnamese
- ☐ Zapoteco
- ☐ Other
- ☐ Prefer not to answer

Specify other languages read, understood or spoken at home. ______________________________

What is your preferred language at home?

- ◯ English
- ◯ Spanish
- ◯ Albanian
- ◯ Apache
- ◯ Arabic
- ◯ Bengali/Bangla
- ◯ Bhutanese
- ◯ Burmese
- ◯ Cantonese
- ◯ Cape Verdean Creole
- ◯ Creole
- ◯ Chamoru
- ◯ Chuukese
- ◯ Dakota
- ◯ Fijian
- ◯ French
- ◯ Hawaiian
- ◯ Hmong
- ◯ Ilokano
- ◯ Karen
- ◯ Khmer/Cambodian
- ◯ Kinyarwanda
- ◯ Korean
- ◯ Kosraean
- ◯ Lakota
- ◯ Lingala
- ◯ Mam
- ◯ Mandarin
- ◯ Marshallese
- ◯ Mixteco
- ◯ Nakota
- ◯ Navajo
- ◯ Nepali
- ◯ Portuguese
- ◯ Pohnpeian
- ◯ Russian
- ◯ Sign Language
- ◯ Somali
- ◯ Samoan
- ◯ Swahili
- ◯ Tagalog
- ◯ Thai
- ◯ Tongan
- ◯ Triqui
- ◯ Vietnamese
- ◯ Zapoteco
- ◯ Other
- ◯ Prefer not to answer

Specify other preferred language.

____

Family Income

In 2019, what was your total household income before taxes?

- ◯ Less than $15,000
- ◯ $15,000 - $19,999
- ◯ $20,000 - $24,999
- ◯ $25,000 - $34,999
- ◯ $35,000 - $49,999
- ◯ $50,000 - $74,999
- ◯ $75,000 - $99,999
- ◯ $100,000 and above
- ◯ Prefer not to answer

Work Ppe And Distancing

Date of Work PPE and Distancing Collection	______________________ (MM/DD/YYYY)
In your workplace, do you have access to necessary facilities to wash?	◯ Yes, all of the time<br>◯ Yes, most of the time<br>◯ Some of the time<br>◯ Rarely<br>◯ Not at all<br>◯ Prefer not to answer
Does your work require you to be in close contact (i.e. within 6 ft) with others?	◯ Yes, all of the time<br>◯ Yes, most of the time<br>◯ Some of the time<br>◯ Rarely<br>◯ Not at all<br>◯ Prefer not to answer
In your workplace, do you have access to necessary personal protective equipment (PPE)?	◯ Yes, all of the time<br>◯ Yes, most of the time<br>◯ Some of the time<br>◯ Rarely<br>◯ Not at all<br>◯ Not applicable<br>◯ Prefer not to answer

Medical History

Medical History

Date of Medical History Collection

____
(MM/DD/YYYY)

Conditions

Do you have any of the following conditions? (Select all that apply)

Immunocompromised condition	◯ Yes ◯ No ◯ Prefer not to answer
Autoimmune disease	◯ Yes ◯ No ◯ Prefer not to answer
Hypertension (HTN, high blood pressure)	◯ Yes ◯ No ◯ Prefer not to answer
Diabetes	◯ Yes ◯ No ◯ Prefer not to answer
Chronic kidney disease (CKD)	◯ Yes ◯ No ◯ Prefer not to answer
Cancer diagnosis and/or treatment within the past 12 months	◯ Yes ◯ No ◯ Prefer not to answer
Cardiovascular disease (CVD or heart disease)	◯ Yes ◯ No ◯ Prefer not to answer
Asthma	◯ Yes ◯ No ◯ Prefer not to answer
Chronic obstructive pulmonary disease (COPD)	◯ Yes ◯ No ◯ Prefer not to answer
Other chronic lung disease	◯ Yes ◯ No ◯ Prefer not to answer
Sickle Cell Anemia	◯ Yes ◯ No ◯ Prefer not to answer
Depression	◯ Yes ◯ No ◯ Prefer not to answer
Alcohol or substance use disorder	◯ Yes ◯ No ◯ Prefer not to answer
Intravenous drug use	◯ Yes ◯ No ◯ Prefer not to answer
Other mental health disorder	◯ Yes ◯ No ◯ Prefer not to answer
Other chronic condition	◯ Yes ◯ No ◯ Prefer not to answer

Health Status

Date of Health Status Collection

____
(MM/DD/YYYY)

Height

How tall are you without shoes?

Please choose the units you would like to use for height

- ◯ Feet and inches
- ◯ Meters and centimeters
- ◯ Don't know
- ◯ Prefer not to answer

Feet

____

Inches

____

Meters

____

Centimeters

____

Weight

Please choose the units you would like to use for weight

- ◯ Kilograms
- ◯ Pounds
- ◯ Prefer not to answer

How much do you weigh without clothes or shoes?

If you are currently pregnant, how much did you weigh before your pregnancy?

____

How much do you weigh without clothes or shoes?

If you are currently pregnant, how much did you weigh before your pregnancy?

____

Self-reported Health

Would you say your health in general is excellent, very good, good, fair, or poor?

- ◯ Excellent
- ◯ Very good
- ◯ Good
- ◯ Fair
- ◯ Poor
- ◯ Prefer not to answer
- ◯ Don't know

Do you have a disability that interferes with your ability to carry out daily activities? Examples of daily activities include walking, climbing stairs, shopping, balancing a checkbook, bathing or dressing.

- ◯ Yes
- ◯ No
- ◯ Prefer not to answer

Vaccine Acceptance

Date of Vaccine Acceptance Collection

____
(MM/DD/YYYY)

Vaccination

Have you ever received a flu vaccination?
- ◯ Yes
- ◯ No
- ◯ Don't know
- ◯ Prefer not to answer

Have you received a flu vaccine in the last 12 months?
- ◯ Yes
- ◯ No
- ◯ Don't know
- ◯ Prefer not to answer

Have you received a COVID-19 vaccine?
- ◯ Yes
- ◯ No
- ◯ Prefer not to answer
- ◯ Don't know

Reasons for Getting/Not Getting a COVID 19 Vaccine

Why would/did you get a COVID-19 vaccine?
- ☐ I want(ed) to keep my family safe
- ☐ I want(ed) to keep my community safe
- ☐ I want(ed) to keep myself safe
- ☐ I have(had) a chronic health problem, like asthma or diabetes
- ☐ My doctor told me to get a COVID-19 vaccine
- ☐ I don't(didn't) want to get really sick from COVID-19
- ☐ I want(ed) to feel safe around other people
- ☐ I believe(d) life won't go back to normal until most people get a COVID-19 vaccine
- ☐ Required by my school or workplace
- ☐ Required for travel
- ☐ Other
- ☐ Not Applicable
- ☐ Prefer not to answer

(Check all that apply)

Why would you/did you NOT get a COVID-19 vaccine?

- ☐ I am/was allergic to vaccines
- ☐ I don't/didn't like needles
- ☐ I am/was not concerned about getting really sick from COVID-19
- ☐ I am/was concerned about side effects from the vaccine
- ☐ I don't/didn't think vaccines work very well
- ☐ I don't/didn't trust that the vaccine will be safe
- ☐ I don't/didn't believe the COVID-19 pandemic is as bad as some people say it is
- ☐ I don't/didn't want to pay for it
- ☐ I don't/didn't know enough about how well a COVID-19 vaccine works
- ☐ Other
- ☐ Not Applicable
- ☐ Prefer not to answer

(Check all that apply)

Testing

Date of Testing Collection	______________________ (MM/DD/YYYY)
If you were to test positive for COVID-19, would you be able to isolate without losing your job?	◯ Yes<br>◯ No<br>◯ Don't know<br>◯ Prefer not to answer
If you would be exposed to someone with COVID-19, would you be able to quarantine without losing your job?	◯ Yes<br>◯ No<br>◯ Don't know<br>◯ Prefer not to answer

Tested previously for COVID-19

Have you ever been tested for COVID-19?	◯ Yes<br>◯ No<br>◯ Don't know<br>◯ Prefer not to answer

Tested positive for COVID-19

Have you ever tested positive for COVID-19?	◯ Yes<br>◯ No<br>◯ Don't know<br>◯ Prefer not to answer
What month did you first test positive for COVID-19?	◯ January<br>◯ February<br>◯ March<br>◯ April<br>◯ May<br>◯ June<br>◯ July<br>◯ August<br>◯ September<br>◯ October<br>◯ November<br>◯ December<br>◯ Prefer not to answer
What year did you first test positive for COVID-19?	◯ 2019<br>◯ 2020<br>◯ 2021<br>◯ 2022<br>◯ Prefer not to answer

What month did you have your most recent COVID-19 test?	◯ January<br>◯ February<br>◯ March<br>◯ April<br>◯ May<br>◯ June<br>◯ July<br>◯ August<br>◯ September<br>◯ October<br>◯ November<br>◯ December<br>◯ Prefer not to answer
What year did you have your most recent COVID-19 test?	◯ 2019<br>◯ 2020<br>◯ 2021<br>◯ 2022<br>◯ Prefer not to answer
What was the result of your most recent COVID-19 test?	◯ Negative<br>◯ Positive<br>◯ Never obtained results<br>◯ Indeterminate<br>◯ Don't know<br>◯ Prefer not to answer
How were you tested for your most recent test?	◯ Nasal Swab<br>◯ Throat Swab<br>◯ Blood Sample<br>◯ Saliva<br>◯ Prefer not to answer

Accessibility to testing

I know where I can get COVID-19 testing in my community.	◯ Strongly disagree<br>◯ Disagree<br>◯ Neither disagree or agree<br>◯ Agree<br>◯ Strongly agree<br>◯ Prefer not to answer
It is easy to get tested for COVID-19.	◯ Strongly disagree<br>◯ Disagree<br>◯ Neither disagree or agree<br>◯ Agree<br>◯ Strongly agree<br>◯ Prefer not to answer

Covid Test

This is for projects that are doing acute testing. To collect as part of the testing procedure by the study team. For many projects some of these fields may be prefilled, such as location, method target, test name, specimen type, specimen collector. Testing results will need to be filled in after collection

Date of COVID Test Information Collection	______________________ (MM/DD/YYYY)
Participant Testing Disease Status	☐ Asymptomatic<br>☐ Pre-symptomatic illness<br>☐ Mild/Moderate outpatient illness<br>☐ Acute illness<br>☐ Severe/Critical inpatient illness<br>☐ Exposed<br>☐ Convalescent illness
Quality and Regulatory	○ CLIA/CP certified<br>○ CLIA Waiver<br>○ FDA authorized (EUA)<br>○ FDA cleared<br>○ LDT<br>○ Other (specify)
Other approval	______________________
Test Collection Setting	○ Clinic<br>○ Drive-through<br>○ Home<br>○ Mobile unit<br>○ Lab<br>○ Mail-in<br>○ Community location (e.g., church, school, community center, etc.)<br>○ Other, Specify
Other setting	______________________
Test Performed Location	○ Clinic<br>○ Drive-through<br>○ Home<br>○ Mobile unit<br>○ Lab<br>○ Mail-in<br>○ Community location (e.g., church, school, community center, etc.)<br>○ Other, Specify
Other performed location	______________________

Study Setting	◯ Community health center<br>◯ Nursing home or long-term care facility<br>◯ Prison or correctional facility<br>◯ Public housing<br>◯ Rural<br>◯ Urban<br>◯ School<br>◯ In-home<br>◯ Other, Specify
Other study setting	______________________
Test Method Target	◯ Antibody<br>◯ Antigen<br>◯ Nucleic acid/PCR<br>◯ Nucleic acid/Isothermal<br>◯ Molecular/host response<br>◯ Biochemical marker (eg, pH)<br>◯ Other, Specify
Other method target	______________________
Test manufacturer (or LDT) and test name	______________________
Specimen Type	◯ Anterior nasal swab<br>◯ Mid-turbinate nasal swab<br>◯ Nasopharyngeal swab<br>◯ Oropharyngeal swab<br>◯ Nasal lavage<br>◯ Saliva<br>◯ Sputum<br>◯ Whole blood<br>◯ Other, Specify
Other specimen type	______________________
Specimen Collector	◯ Self-collect<br>◯ Health Care Provider collected<br>◯ Other, Specify
Other specimen collector	______________________
Date and time specimen collected	______________________
Date and time result received	______________________
Date and time result sent to participant	______________________
Raw test result (if not a Positive/Negative/Failed report)	______________________

Test result	◯ Positive<br>◯ Negative<br>◯ Failed<br>◯ Lost<br>◯ Other
Other test result	__________________________

Symptoms

This is for projects that are doing acute testing. To collect as part of the testing procedure by the study team.

Date of Symptom Collection

____
(MM/DD/YYYY)

Current Symptoms

Have you had any of these symptoms during the past week?

	Yes	No	Don't know	Prefer not to answer
Fever or chills	○	○	○	○
Cough	○	○	○	○
Shortness of breath or difficulty breathing	○	○	○	○
Lack of energy or general tired feeling	○	○	○	○
Muscle or body aches	○	○	○	○
Headache	○	○	○	○
New loss of taste or smell	○	○	○	○
Sore throat, congestion or runny nose	○	○	○	○
Feeling sick to your stomach or vomiting, diarrhea	○	○	○	○
Abdominal Pain	○	○	○	○
Skin Rash	○	○	○	○
Other	○	○	○	○

Alcohol And Tobacco

Date of Alcohol/Tobacco Use Collection

____
(MM/DD/YYYY)

Alcohol and Tobacco/Nicotine Use

In your entire life, have you had at least 1 drink of any kind of alcohol, not counting small tastes or sips?

- ◯ Yes
- ◯ No
- ◯ Prefer not to answer

How often do you have a drink containing alcohol?

- ◯ Never
- ◯ Monthly or less
- ◯ 2-4 times a month
- ◯ 2-3 times a week
- ◯ 4 or more times a week
- ◯ Don't know or refuse to answer

Do you now smoke cigarettes?

- ◯ Every Day
- ◯ Some Days
- ◯ Rarely
- ◯ Not at all
- ◯ Prefer not to answer
- ◯ Don't know

If you smoke every day, on average, how many cigarettes per day do you smoke?

____

Do you now use electronic cigarettes every day, some days, rarely, or not at all?

- ◯ Every Day
- ◯ Some Days
- ◯ Rarely
- ◯ Not at all
- ◯ Prefer not to answer
- ◯ Don't know

Identity

About you

Date of Identity Collection

(MM/DD/YYYY)

First Name

Last Name

Street Address

Street Address 2

City

State or Territory	◯ Alabama<br>◯ Alaska<br>◯ Arizona<br>◯ Arkansas<br>◯ California<br>◯ Colorado<br>◯ Connecticut<br>◯ Delaware<br>◯ District of Columbia(DC)<br>◯ Florida<br>◯ Georgia<br>◯ Hawaii<br>◯ Idaho<br>◯ Illinois<br>◯ Indiana<br>◯ Iowa<br>◯ Kansas<br>◯ Kentucky<br>◯ Louisiana<br>◯ Maine<br>◯ Maryland<br>◯ Massachusetts<br>◯ Michigan<br>◯ Minnesota<br>◯ Mississippi<br>◯ Missouri<br>◯ Montana<br>◯ Nebraska<br>◯ Nevada<br>◯ New Hampshire<br>◯ New Jersey<br>◯ New Mexico<br>◯ New York<br>◯ North Carolina<br>◯ North Dakota<br>◯ Ohio<br>◯ Oklahoma<br>◯ Oregon<br>◯ Pennsylvania<br>◯ Rhode Island<br>◯ South Carolina<br>◯ South Dakota<br>◯ Tennessee<br>◯ Texas<br>◯ Utah<br>◯ Vermont<br>◯ Virginia<br>◯ Washington<br>◯ West Virginia<br>◯ Wisconsin<br>◯ Wyoming<br>◯ American Somoa<br>◯ GUAM<br>◯ Northern Mariana Islands<br>◯ Puerto Rico<br>◯ US Virgin Islands
Mobile Phone	________________________________
Home Phone	________________________________

Other Phone	__________________________________
Personal Email	__________________________________
Other Email	__________________________________
Preferred Method of Contact	◯ Mobile phone<br>◯ Home phone<br>◯ Other phone<br>◯ Personal email<br>◯ Other email<br>◯ Prefer not to answer
Date of Birth	__________________________________<br>(MM/DD/YYYY)

Tier2 Sociodemographics

Are any of these a closer description of how you think of yourself?	◯ Queer<br>◯ Polysexual, omnisexual, sapiosexual or pansexual<br>◯ Asexual or Asexual Spectrum<br>◯ Two-spirit<br>◯ Have not figured out or are in the process of figuring out your sexuality<br>◯ Mostly straight, but sometimes attracted to people of your own sex<br>◯ Do not think of yourself as having sexuality<br>◯ Do not use labels to identity yourself<br>◯ Don't know the answer<br>◯ No, I have a different description and would like to specify<br>◯ Prefer not to answer
Specify your description of how you think of yourself.	______________________________

Tier2 Medical History

Missed medical procedure

Since the start of the COVID-19 pandemic (March 2020), have you needed to postpone any medical care?	◯ Yes ◯ No ◯ Prefer not to answer

Tier2 Disability

Date of Disability Collection	______________________ (MM/DD/YYYY)
Are you deaf, or do you have serious difficulty hearing?	◯ Yes ◯ No ◯ Prefer not to answer
Are you blind, or do you have serious difficulty seeing, even when wearing glasses?	◯ Yes ◯ No ◯ Prefer not to answer
Because of a physical, mental, or emotional condition, do you have serious difficulty concentrating, remembering, or making decisions?<br><br>(5 years of age or older)	◯ Yes ◯ No ◯ Prefer not to answer
Do you have serious difficulty walking or climbing stairs?<br><br>(5 years of age or older)	◯ Yes ◯ No ◯ Prefer not to answer
Do you have difficulty dressing or bathing?<br><br>(5 years of age or older)	◯ Yes ◯ No ◯ Prefer not to answer
Because of a physical, mental, or emotional condition, do you have difficulty doing errands alone such as visiting a doctor's office or shopping?<br><br>(15 years of age or older)	◯ Yes ◯ No ◯ Prefer not to answer

Tier2 Vaccine Acceptance

In deciding whether to get the COVID-19 vaccine, how important are/were these statements to you ?
1 = Not Important, 3 = Neutral, 5 = Very Important

	1- Not important	2	3- Neutral	4	5- Very important	Prefer not to answer
The vaccine is safe	◯	◯	◯	◯	◯	◯
The vaccine prevents COVID-19	◯	◯	◯	◯	◯	◯
The vaccine is free or low cost	◯	◯	◯	◯	◯	◯
The vaccine is not painful	◯	◯	◯	◯	◯	◯
Convenience in where and when I can get it	◯	◯	◯	◯	◯	◯

Who was the manufacturer of the most recent vaccine you received?

◯ Pfizer
◯ Moderna
◯ Johnson & Johnson
◯ Other
◯ Don't know
◯ Prefer not to answer

Who was the manufacturer of your most recent vaccine?

____

How many doses have you received?

____

On what date did you receive your first shot of the vaccine?

____
(MM/DD/YYYY)

On what date did you receive your most recent shot of the vaccine?

____
(MM/DD/YYYY)

Prior to your vaccination, were you given information or brochures about the vaccine and/or COVID-19?

◯ Yes
◯ No
◯ Don't know
◯ Prefer not to answer
(This would include materials or discussions with your doctor, your library or church, a government health agency, university/college, or community health organization.)

After your vaccination, were you given information or brochures about the vaccine and/or COVID-19?

◯ Yes
◯ No
◯ Don't know
◯ Prefer not to answer
(This would include materials or discussions with your doctor, your library or church, a government health agency, university/college, or community health organization.)

How often do you need someone to help you to understand information or guidance from your doctor, nurse or pharmacist?	◯ Often<br>◯ Sometimes<br>◯ Rarely<br>◯ Don't know<br>◯ Prefer not to answer
To the best of your knowledge, have you previously been exposed to someone who has had a positive COVID-19 test?	◯ Yes<br>◯ No<br>◯ Don't know<br>◯ Prefer not to answer
On a scale of 1-5, How informed are you that you know which vaccine option is right for you? (1 is least informed, 5 is most informed)	◯ 1- Least informed<br>◯ 2<br>◯ 3<br>◯ 4<br>◯ 5 - Most informed<br>◯ Don't know<br>◯ Prefer not to answer
Is the vaccine required by your school or job?	◯ Yes<br>◯ No<br>◯ Don't know<br>◯ Prefer not to answer
If you are given a recommendation for a vaccine booster, will you get a booster?	◯ Yes<br>◯ No<br>◯ Don't know<br>◯ Prefer not to answer

Tier2 Testing

Perceived accuracy of testing

How confident are you that a negative test result means that you do not have COVID-19?

- ◯ Not at all confident
- ◯ Somewhat confident
- ◯ Confident
- ◯ Very confident
- ◯ Prefer not to answer

How confident are you that a positive test result means that you do have COVID-19?

- ◯ Not at all confident
- ◯ Somewhat confident
- ◯ Confident
- ◯ Very confident
- ◯ Prefer not to answer

Perceived benefits of testing

How much do the following encourage you to get tested?

	Not at all	Slightly	Somewhat	Moderately	Very much	Prefer not to answer
Reduce worry that I might have COVID-19.	◯	◯	◯	◯	◯	◯
Believe that I was exposed to someone who has COVID-19.	◯	◯	◯	◯	◯	◯
To know if I am safe not to give COVID-19 to friends and family.	◯	◯	◯	◯	◯	◯
To know if I am safe not to give COVID-19 to anyone I am around.	◯	◯	◯	◯	◯	◯
To let my employer know that I am safe to work.	◯	◯	◯	◯	◯	◯
To get treated early (if I am positive).	◯	◯	◯	◯	◯	◯

Perceived risks of testing

How much do the following discourage you to get tested?

	Not at all	Slightly	Somewhat	Moderately	Very much	Prefer not to answer
May experience discomfort from being tested.	◯	◯	◯	◯	◯	◯
Even if I don't have it when tested, I can still get COVID-19 later.	◯	◯	◯	◯	◯	◯

I don't have COVID-19 symptoms so I don't need to be tested.	◯	◯	◯	◯	◯	◯
If I'm positive, officials will need to contact the people I've been in contact with.	◯	◯	◯	◯	◯	◯
I don't want to know if I have it.	◯	◯	◯	◯	◯	◯
Not much they can do for me if I have it.	◯	◯	◯	◯	◯	◯
Difficult to get needed healthcare if I have it.	◯	◯	◯	◯	◯	◯

Intention to be tested

I plan to get tested as often as needed.

- ◯ Strongly Disagree
- ◯ Disagree
- ◯ Neither disagree or agree
- ◯ Agree
- ◯ Strongly agree
- ◯ Prefer not to answer

Interpretation of negative or positive results

If I get a negative test result, it means

[check all that apply]:

- ☐ I don't have to worry about getting COVID-19
- ☐ I don't have COVID-19 now
- ☐ I can be around others without giving the virus to them
- ☐ I can be around others without getting the virus from them
- ☐ Prefer not to answer

(Check all that apply)

If I get a positive result, it means:

[check all that apply]

- ☐ I will need to be admitted to the hospital
- ☐ I will need to isolate myself from others
- ☐ I will need to take off work
- ☐ Prefer not to answer

(Check all that apply)

Has any one close to you:

	Yes	No	Don't know	Prefer not to answer
Become sick from COVID-19?	◯	◯	◯	◯
Been hospitalized from COVID-19?	◯	◯	◯	◯
Died from COVID-19?	◯	◯	◯	◯

Tier 2 Covid

Have you had COVID?	◯ Yes<br>◯ No<br>◯ Don't know<br>◯ Prefer not to answer
What do you think your personal level of risk is for getting sick from COVID-19?	◯ Low Risk<br>◯ Medium Risk<br>◯ High Risk<br>◯ Don't know<br>◯ Prefer not to answer
Have you been tested for COVID-19 in the last 30 days?	◯ Yes<br>◯ No<br>◯ Don't know<br>◯ Prefer not to answer
Why were you tested? (Select the primary reason for your latest test.)	◯ Required for work<br>◯ I had symptoms<br>◯ I had contact with someone who tested positive or was sick<br>◯ I had no symptoms, but wanted to know if I was infected<br>◯ Other<br>◯ Don't know<br>◯ Prefer not to answer
Other reason why you were tested.	__________________________________
Have you encountered any of the following barriers or problems with testing? (Select all that apply.)	☐ Need to take time off work to get tested<br>☐ Out of pocket costs for test<br>☐ Out of pocket costs for transportation, childcare, or time off work to get tested<br>☐ I do not know where to go to be tested<br>☐ Pain or discomfort from the test<br>☐ Saliva<br>☐ Concern about others handling my personal data<br>☐ Other<br>☐ None of the above<br>☐ Don't know<br>☐ Prefer not to answer
Other barriers or problems encountered.	__________________________________
Does your employer offer paid time off if you test positive?	◯ Yes<br>◯ No<br>◯ Don't know<br>◯ Prefer not to answer

If you needed to isolate due to a positive test or illness, what challenges do you face?	☐ There are other people in my household<br>☐ There are children in my household<br>☐ There are older adults in my household<br>☐ I don't have a good place where I could isolate<br>☐ No one to help me if I am sick<br>☐ Lost income or wages<br>☐ People might blame me or treat me badly<br>☐ Other<br>☐ None of the above<br>☐ Don't know<br>☐ Prefer not to answer
Other challenges to isolation.	__________________________________
For your previous covid testing, how long did you wait between scheduling the test and when the test was performed?	○ Same day as scheduled<br>○ Within a few days<br>○ Within a week/same week<br>○ Within the same month<br>○ More than a month<br>○ Prefer not to answer
How are test results communicated?	☐ Phone call<br>☐ Email<br>☐ Text<br>☐ In person<br>☐ Prefer not to answer

Tier2 Medications

Date of Medication Collection

____
(MM/DD/YYYY)

Medications

The US Food and Drug Administration (FDA) maintains a searchable database of brand name drugs, generic drugs and therapeutic biological products that can assist with classification and action of medications.

Do you currently take prescription medications?

- ◯ Yes
- ◯ No
- ◯ Prefer not to answer or do not remember

Prescription Medication 1

____

Prescription Medication 2

____

Prescription Medication 3

____

Prescription Medication 4

____

Prescription Medication 5

____

Prescription Medication 6

____

Prescription Medication 7

____

Prescription Medication 8

____

Prescription Medication 9

____

Prescription Medication 10

____

Prescription Medication 11

____

Prescription Medication 12

____

Prescription Medication 13

____

Prescription Medication 14

____

Prescription Medication 15

____

Prescription Medication 15

Prescribed medications unable to transcribe:

Tier2 Alcohol And Tobacco

How many drinks containing alcohol do you have on a typical day when you are drinking?	◯ 1 or 2<br>◯ 3 or 4<br>◯ 5 or 6<br>◯ 7, 8 or 9<br>◯ 10 or more<br>◯ Prefer not to answer
How many years have you smoked?	______________________
How many years have you vaped?	______________________

Tier2 Drug Use

Date of Drug Use Collection	______________________ (MM/DD/YYYY)
Have you used marijuana in the past 12 months?	◯ Yes<br>◯ No<br>◯ Prefer not to answer
If you have used marijuana in the past 12 months, have often have you smoked it?	◯ Daily or almost daily<br>◯ About once or twice per week<br>◯ About once per month<br>◯ Rarely (less than once per month)<br>◯ Never<br>◯ Prefer not to answer
If you have used marijuana in the past 12 months, have often have you vaped it?	◯ Daily or almost daily<br>◯ About once or twice per week<br>◯ About once per month<br>◯ Rarely (less than once per month)<br>◯ Never<br>◯ Prefer not to answer
In the past 12 months, have often have you used prescription drugs just for the feeling, more than prescribed, or that were not prescribed for you?	◯ Daily or almost daily<br>◯ About once or twice per week<br>◯ About once per month<br>◯ Rarely (less than once per month)<br>◯ Never<br>◯ Prefer not to answer
In the past 12 months, have you used any of the following drugs: cocaine or crack, heroin, crystal meth (methamphetamine), hallucinogens (like LSD, psilocybin, PCP, ketamine), ecstasy?	◯ Yes<br>◯ No<br>◯ Prefer not to answer

How often have you used each of the following drugs?

Cocaine or crack	◯ Daily or almost daily<br>◯ About once or twice per week<br>◯ About once per month<br>◯ Rarely (less than once per month)<br>◯ Never<br>◯ Prefer not to answer
Heroin	◯ Daily or almost daily<br>◯ About once or twice per week<br>◯ About once per month<br>◯ Rarely (less than once per month)<br>◯ Never<br>◯ Prefer not to answer
Crystal meth (methamphetamine)	◯ Daily or almost daily<br>◯ About once or twice per week<br>◯ About once per month<br>◯ Rarely (less than once per month)<br>◯ Never<br>◯ Prefer not to answer

Hallucinogens (like LSD, psilocybin, PCP, ketamine)	◯ Daily or almost daily<br>◯ About once or twice per week<br>◯ About once per month<br>◯ Rarely (less than once per month)<br>◯ Never<br>◯ Prefer not to answer
Ecstasy	◯ Daily or almost daily<br>◯ About once or twice per week<br>◯ About once per month<br>◯ Rarely (less than once per month)<br>◯ Never

Tier2 Food Insecurity

Food Insecurity:

I'm going to read you two statements that people have made about their food situation.

Please tell me whether the statement was OFTEN, SOMETIMES, or NEVER true for (you/you and the other members of your household) in the last 12 months.

The first statement is,<br><br>"The food that (I/we) bought just didn't last, and (I/we) didn't have money to get more."<br><br>Was that often, sometimes, or never true for (you/your household) in the last 12 months?	◯ Often true<br>◯ Sometimes true<br>◯ Never true<br>◯ Don't know<br>◯ Prefer not to answer
The second statement is,<br><br>"(I/we) couldn't afford to eat balanced meals."<br><br>Was that often, sometimes, or never true for (you/your household) in the last 12 months?	◯ Often true<br>◯ Sometimes true<br>◯ Never true<br>◯ Don't know<br>◯ Prefer not to answer
In the last 12 months, since (date 12 months ago) did (you/you or other adults in your household) ever cut the size of your meals or skip meals because there wasn't enough money for food?	◯ Yes<br>◯ No<br>◯ Don't know<br>◯ Prefer not to answer
How often did this happen - almost every month, some months but not every month, or in only 1 or 2 months?	◯ Almost every month<br>◯ Some months but not every month<br>◯ Only 1 or 2 months<br>◯ Don't know<br>◯ Prefer not to answer
In the last 12 months, did you ever eat less than you felt you should because there wasn't enough money to buy food?	◯ Yes<br>◯ No<br>◯ Don't know<br>◯ Prefer not to answer
In the last 12 months, were you ever hungry but didn't eat because you couldn't afford enough food?	◯ Yes<br>◯ No<br>◯ Don't know<br>◯ Prefer not to answer
The fresh fruits and vegetables in my neighborhood are of high quality	◯ Completely agree<br>◯ Somewhat agree<br>◯ Neutral/no opinion<br>◯ Somewhat disagree<br>◯ Strongly disagree<br>◯ Don't know<br>◯ Prefer not to answer

Tier2 Housing

In the past two months, have you been staying in the same place?	◯ Yes<br>◯ No<br>◯ Prefer not to answer<br>◯ Don't know
Are you worried or concerned that in the next two months you may NOT have a place to stay?	◯ Yes<br>◯ No<br>◯ Prefer not to answer<br>◯ Don't know

Tier2 Trust

How much do you trust each of these sources to provide correct information about COVID 19? (Select one response for each row.)

	Not at all	A little	Somewhat	A great deal	Don't know	Prefer not to answer
Your doctor or health care provider	○	○	○	○	○	○
Your faith leader	○	○	○	○	○	○
Your close friends and members of your family	○	○	○	○	○	○
People you go to work or class with or other people you know	○	○	○	○	○	○
News on the radio, TV, online, or in newspapers	○	○	○	○	○	○
Your contacts on social media	○	○	○	○	○	○
The U.S. government	○	○	○	○	○	○
The U.S. Coronavirus Task Force	○	○	○	○	○	○

Tier2 Mrn

Medical Record Number

____

Medical Record Number Organziation

____

Tier2 Other

In what language is this survey being provided?	◯ English<br>◯ Spanish<br>◯ Chinese<br>◯ Other
In what language is this survey being provided, specify	__________________________
This variable is used to separate aims being submitted to the CDCC in the same file. All records from the same aim should have a common label.	__________________________
Which set of CDEs does this record represent? Some projects collected data prior to implementation of NIH approved CDE exceptions.	◯ CDEs collected prior to completion of NIH exception process<br>◯ CDEs collected with NIH approved exceptions implemented
If you have processed sequence data you have submitted to GenBank, enter your GenBank accession number here.	__________________________<br>(Your processed PCR sequence data can be uploaded to GenBank at https://submit.ncbi.nlm.nih.gov/)
Zip Code (3 digit only)	__________________________
What is the source of these data?	◯ Participant Direct Report<br>◯ Secondary Medical Record Dataset<br>◯ Mixed

Tier2 Fam

Who is answering this survey?	◯ Minor self-report only<br>◯ Parent/guardian proxy of a participant who is a minor<br>◯ Minor self-report AND parent/guardian proxy<br>◯ Parent/guardian self-report
What is the unique identifier for the family to which this individual belongs?	__________________________________

Tier2 Ped

Date of Pediatric Data Collection

____
(MM/DD/YYYY)

Baseline Child Health

Did your child have any of the following premature or neonatal conditions?

- ☐ Fetal malnutrition
- ☐ Extreme immaturity
- ☐ Cerebral hemorrhage at birth
- ☐ Spinal cord injury at birth
- ☐ Birth asphyxia
- ☐ Respiratory diseases
- ☐ Hypoxic-ischemic encephalopathy
- ☐ Other
- ☐ Prefer not to answer

(0-2) Do you know your child's head circumference?

- ◯ Yes
- ◯ No
- ◯ Child at least 2 years old
- ◯ Prefer not to answer

How many centimeters?

____

(0-1) Is your child being breastfed or fed pumped milk?

- ◯ Yes
- ◯ No
- ◯ Child is not in first year of life
- ◯ Prefer not to answer

COVID-19 Vaccination History

If your child had long COVID/post-acute sequelae of COVID-19 (PASC) symptoms at the time of vaccination, did those symptoms change?

- ◯ Yes
- ◯ No
- ◯ Child is not in first year of life
- ◯ Prefer not to answer

Are the patient's immunizations up to date for their age at the time of COVID-19diagnosis/assessment?

- ◯ Yes
- ◯ No
- ◯ Don't know
- ◯ Prefer not to answer

If immunizations are not up to date, what is/are the reason(s) for not being up to date? (Check all that apply.)

- ☐ Clinic was closed because of COVID-19
- ☐ Child had symptoms of COVID-19, so you cancelled appointment
- ☐ You cancelled appointments to avoid being around others/in a healthcare setting
- ☐ Other reasons related to COVID-19
- ☐ Other reasons not related to COVID-19
- ☐ Prefer not to answer

Has your child received any MMR vaccinations?

- ◯ Yes
- ◯ No
- ◯ Don't know
- ◯ Prefer not to answer

Has your child received the current seasonal influenza vaccine?	◯ Yes<br>◯ No<br>◯ Don't know<br>◯ Prefer not to answer
Has your child received palivizumab for prevention of respiratory syncytial virus (RSV)?	◯ Yes<br>◯ No<br>◯ Don't know<br>◯ Prefer not to answer
Has your child received the BCG vaccination?	◯ Yes<br>◯ No<br>◯ Don't know<br>◯ Prefer not to answer
Date of most recent vaccination (excluding vaccination for COVID-19)	____________________<br>(MM/DD/YYYY)
Respiratory support prior to onset of COVID-19	◯ Yes<br>◯ No<br>◯ Don't know<br>◯ Prefer not to answer

Vital Signs

Body temperature (degrees Celsius)	____________________
Heart rate (beats/min)	____________________
Systolic blood pressure (mmHg)	____________________
Diastolic blood pressure (mmHg)	____________________
Respiratory rate (breaths/min)	____________________
Oxygen saturation (%)	____________________
Supplemental oxygen	◯ Yes<br>◯ No<br>◯ Don't know<br>◯ Prefer not to answer
Date and time of vital signs	____________________

Symptoms/Physical Findings

Which of the following acute COVID/MIS-C symptoms were experienced at any time point during current illness?

	Yes	No	Don't know	Prefer not to answer
Abdominal pain	○	○	○	○
Bleeding	○	○	○	○
Chest pain	○	○	○	○
Cough	○	○	○	○
Cyanosis (bluish lips/face)	○	○	○	○
Diarrhea	○	○	○	○
Fatigue	○	○	○	○
Fever	○	○	○	○
Headache	○	○	○	○
Muscle or body aches	○	○	○	○
Nasal congestion or runny nose	○	○	○	○
Nausea/vomiting	○	○	○	○
Neck pain	○	○	○	○
New loss of taste or smell	○	○	○	○
Palpitations	○	○	○	○
Shortness of breath or difficulty breathing	○	○	○	○
Skin rash	○	○	○	○
Sore throat	○	○	○	○
Subjective fever/chills/rigors/night sweats	○	○	○	○
Swollen glands	○	○	○	○
Conjunctivitis	○	○	○	○
Oral mucosal change	○	○	○	○
Changes in hands and feet	○	○	○	○
Other symptoms	○	○	○	○
None of the above/asymptomatic	○	○	○	○

Which of the following acute symptoms were confirmed by physical exam?

	Yes	No	Prefer not to answer
Abdominal pain	○	○	○
Bleeding	○	○	○
Chest pain	○	○	○
Cough	○	○	○
Cyanosis (bluish lips/face)	○	○	○
Diarrhea	○	○	○

Fatigue	○	○	○
Fever	○	○	○
Headache	○	○	○
Muscle or body aches	○	○	○
Nasal congestion or runny nose	○	○	○
Nausea/vomiting	○	○	○
Neck pain	○	○	○
New loss of taste or smell	○	○	○
Palpitations	○	○	○
Shortness of breath or difficulty breathing	○	○	○
Skin rash	○	○	○
Sore throat	○	○	○
Subjective fever/chills/rigors/night sweats	○	○	○
Swollen glands	○	○	○
Conjunctivitis	○	○	○
Oral mucosal change	○	○	○
Changes in hands and feet	○	○	○
Other symptoms	○	○	○
None of the above/asymptomatic	○	○	○

If swollen glands, Cervical lymphadenopathy (at least 1.5 cm in diameter?)

- ○ Yes
- ○ No
- ○ Don't know
- ○ Prefer not to answer

Were swollen, red, or cracked lips; strawberry tongue; and/or erythema of the oral/pharyngeal mucosa present?

- ○ Yes
- ○ No
- ○ Don't know
- ○ Prefer not to answer

What other symptoms (comma-separated)? ______________________________

Which of the following long COVID/PASC symptoms were experienced at any time point during current illness?

	Yes	No	Don't know	Prefer not to answer
Allodynia	○	○	○	○
Altered level of consciousness/confusion	○	○	○	○
Anorexia (decrease in appetite)	○	○	○	○
Anxiety	○	○	○	○

Cannot move and/or feel one side of body or face	○	○	○	○
Depressed mood	○	○	○	○
Dizziness/lightheadedness/black outs	○	○	○	○
Exertional fatigue	○	○	○	○
Forgetfulness	○	○	○	○
Irritability	○	○	○	○
Orthostasis (dizziness/lightheadedness/black outs on sitting up or standing)	○	○	○	○
Joint pain	○	○	○	○
(15+) Hallucinations (seeing or hearing things others do not see or hear)	○	○	○	○
Hypersomnia	○	○	○	○
Insomnia	○	○	○	○
Malaise	○	○	○	○
Muscle weakness	○	○	○	○
Paresthesia (numbness or tingling somewhere in the body)	○	○	○	○
Persistent cough	○	○	○	○
Problems with balance	○	○	○	○
Problems with gait/falls	○	○	○	○
Toe rashes (red/purple sores or blisters on the feet, including the toes)	○	○	○	○
Trouble concentrating or difficulty thinking ("brain fog")	○	○	○	○
Weight loss	○	○	○	○
Failure of expected weight gain	○	○	○	○
Failure of expected linear growth	○	○	○	○
Other symptions	○	○	○	○

Which of the following long symptoms were confirmed by physical exam?

	Yes	No	Prefer not to answer
Allodynia	○	○	○
Altered level of consciousness/confusion	○	○	○
Anorexia (decrease in appetite)	○	○	○
Anxiety	○	○	○

Cannot move and/or feel one side of body or face	○	○	○
Depressed mood	○	○	○
Dizziness/lightheadedness/black outs	○	○	○
Exertional fatigue	○	○	○
Forgetfulness	○	○	○
Irritability	○	○	○
Orthostasis (dizziness/lightheadedness/black outs on sitting up or standing)	○	○	○
Joint pain	○	○	○
(15+) Hallucinations (seeing or hearing things others do not see or hear)	○	○	○
Hypersomnia	○	○	○
Insomnia	○	○	○
Malaise	○	○	○
Muscle weakness	○	○	○
Paresthesia (numbness or tingling somewhere in the body)	○	○	○
Persistent cough	○	○	○
Problems with balance	○	○	○
Problems with gait/falls	○	○	○
Toe rashes (red/purple sores or blisters on the feet, including the toes)	○	○	○
Trouble concentrating or difficulty thinking ("brain fog")	○	○	○
Weight loss	○	○	○
Failure of expected weight gain	○	○	○
Failure of expected linear growth	○	○	○
Other symptions	○	○	○

What other symptoms (comma-separated)?

____

If Orthostatsis, confirmed by changes in heart rate/blood pressure?

○ Yes
○ No
○ Don't know
○ Prefer not to answer

If persistent cough, productive?

○ Yes
○ No
○ Don't know
○ Prefer not to answer

In addition to the above, which of the following were experienced by the infant during illness?

	Yes	No	Don't know	Prefer not to answer
Dehydration	◯	◯	◯	◯
Full or bulging fontanelle	◯	◯	◯	◯
Fussiness	◯	◯	◯	◯
Increased work of breathing/shallow breathing	◯	◯	◯	◯
Lethargy	◯	◯	◯	◯
Poor feeding	◯	◯	◯	◯

Which infant symptoms were confirmed by physical exam?

	Yes	No	Prefer not to answer
Dehydration	◯	◯	◯
Full or bulging fontanelle	◯	◯	◯
Fussiness	◯	◯	◯
Increased work of breathing/shallow breathing	◯	◯	◯
Lethargy	◯	◯	◯
Poor feeding	◯	◯	◯

Date Symptoms Presented (including intermittent symptoms) ______________________________

Are the symptoms ongoing (including intermittent symptoms)?

- ◯ Yes
- ◯ No
- ◯ N/A (no symptoms)
- ◯ Don't know
- ◯ Prefer not to answer

Date Symptoms Resolved ______________________________

Did the patient develop any of the following complications/conditions since the diagnosis of COVID (organized by organ system)?

	Yes	No	Don't know	Prefer not to answer
Fibromyalgia/amplified pain syndrome	◯	◯	◯	◯
Post viral fatigue syndrome	◯	◯	◯	◯
Seizure	◯	◯	◯	◯
Stroke: intracerebral hemorrhage	◯	◯	◯	◯
Stroke: ischemic cerebrovascular accident	◯	◯	◯	◯
Diabetic ketoacidosis (DKA)	◯	◯	◯	◯
New onset diabetes	◯	◯	◯	◯

Pancreatitis	◯	◯	◯	◯
Acute respiratory distress syndrome	◯	◯	◯	◯
Bronchiolitis	◯	◯	◯	◯
Deterioration of prior pulmonary diseases	◯	◯	◯	◯
Lung fibrosis	◯	◯	◯	◯
Pneumonia	◯	◯	◯	◯
Pulmonary embolism	◯	◯	◯	◯
Cardiac arrhythmias	◯	◯	◯	◯
Cardiac failure	◯	◯	◯	◯
Cardiomyopathy	◯	◯	◯	◯
Coronary artery abnormalities	◯	◯	◯	◯
Myocarditis/pericarditis/pericardial effusion	◯	◯	◯	◯
Myositis	◯	◯	◯	◯
Shock	◯	◯	◯	◯
Arthritis	◯	◯	◯	◯
Physical disability/muscular weakness	◯	◯	◯	◯
Acute kidney injury	◯	◯	◯	◯
Acute liver dysfunction	◯	◯	◯	◯
End stage renal disease (ESRD)	◯	◯	◯	◯
Bleeding events	◯	◯	◯	◯
Deep vein thrombosis	◯	◯	◯	◯
Appendicitis	◯	◯	◯	◯
Gastroesophageal reflux disease	◯	◯	◯	◯
Gastrointestinal hemorrhage	◯	◯	◯	◯
Gastrointestinal perforation	◯	◯	◯	◯
Peritonitis	◯	◯	◯	◯
Bacteremia	◯	◯	◯	◯
Pulminary aspergillosis	◯	◯	◯	◯
Toxic shock syndrome	◯	◯	◯	◯
Other (specify)	◯	◯	◯	◯

Date of Onset/Diagnosis ______________________

Where is it located?

◯ Intracranial
◯ Extracranial
◯ Both
◯ Don't know
◯ Prefer not to answer

Date of Resolution ______________________

Any lab tests performed?

- ◯ Yes
- ◯ No
- ◯ Don't know
- ◯ Prefer not to answer

If lab tests were performed, which?

	Yes	No	Don't know	Prefer not to answer
Absolute eosinophil count	◯	◯	◯	◯
Absolute monocyte count	◯	◯	◯	◯
Absolute basophil count	◯	◯	◯	◯
Hemoglobin	◯	◯	◯	◯
Total bilirubin	◯	◯	◯	◯
Prothrombin time (PT)	◯	◯	◯	◯
International normalized ratio (INR)	◯	◯	◯	◯
Activated partial thromboplastin time (aPTT)	◯	◯	◯	◯
IL-6	◯	◯	◯	◯
Complement	◯	◯	◯	◯
Hemoglobin A1C	◯	◯	◯	◯
pH	◯	◯	◯	◯
pCO2	◯	◯	◯	◯
pa02	◯	◯	◯	◯
Calcium	◯	◯	◯	◯
Cerebrospinal fluid (CSF) WBC	◯	◯	◯	◯
CSF red blood cell count (RBC)	◯	◯	◯	◯
CSF Proten	◯	◯	◯	◯
CSF Glucose	◯	◯	◯	◯
Other	◯	◯	◯	◯

Specify

____

Date and Time of Lab Sample Collection

____

Any labs repeated during admission that were more abnormal than initial values?

	Yes	No	Don't know	Prefer not to answer
Absolute eosinophil count	◯	◯	◯	◯
Absolute monocyte count	◯	◯	◯	◯
Absolute basophil count	◯	◯	◯	◯
Hemoglobin	◯	◯	◯	◯

Total bilirubin	◯	◯	◯	◯
Prothrombin time (PT)	◯	◯	◯	◯
International normalized ratio (INR)	◯	◯	◯	◯
Activated partial thromboplastin time (aPTT)	◯	◯	◯	◯
IL-6	◯	◯	◯	◯
Complement	◯	◯	◯	◯
Hemoglobin A1C	◯	◯	◯	◯
pH	◯	◯	◯	◯
pCO2	◯	◯	◯	◯
pa02	◯	◯	◯	◯
Calcium	◯	◯	◯	◯
Cerebrospinal fluid (CSF) WBC	◯	◯	◯	◯
CSF red blood cell count (RBC)	◯	◯	◯	◯
CSF Protein	◯	◯	◯	◯
CSF Glucose	◯	◯	◯	◯
Other	◯	◯	◯	◯

Specify

____

Any other viral testing positive?

◯ Yes
◯ No
◯ Don't know
◯ Prefer not to answer

List other viral tests that were positive.

____

Positive blood culture organism?

____

Positive Culture Date

____

Cardiopulmonary Diagnostic Assessments

Did the patient have any cardiovascular diagnostic assessment performed (beyond physical exam)?

◯ Yes, abnormalities detected
◯ Yes, normal
◯ No, not performed
◯ Don't know
◯ Prefer not to answer

Cardiovascular Diagnostic Assessment Date

____
(MM/DD/YYYY)

What type of abnormalities were detected?

- ◯ Abnormal function
- ◯ Pericardial effusion
- ◯ Coronary artery abnormalities
- ◯ Other
- ◯ Prefer not to answer

What type of assessment was performed

	Yes, abnormalities detected	Yes, normal	No, not performed	Don't know	Prefer not to answer
ECG	◯	◯	◯	◯	◯
ECHO	◯	◯	◯	◯	◯
Cardiac MRI	◯	◯	◯	◯	◯
Other tests	◯	◯	◯	◯	◯

Specify

____

Did the patient have any pulmonary diagnostic testing (beyond physical exam and radiographic testing)?

- ◯ Yes, abnormalities detected
- ◯ Yes, normal
- ◯ No, not performed
- ◯ Don't know
- ◯ Prefer not to answer

Pulmonary Diagnostic Assessment Date

____
(MM/DD/YYYY)

What type of pulmonary diagnostic testing was performed?

	Yes, abnormalities detected	Yes, normal	No, not performed	Don't know	Prefer not to answer
(6+) 6-Minute Walk Test	◯	◯	◯	◯	◯
(6+) Pulmonary Function Test	◯	◯	◯	◯	◯
Co-oxiometry	◯	◯	◯	◯	◯
(6+) 2-Minute Walk Test	◯	◯	◯	◯	◯
Other tests (specify test) performed	◯	◯	◯	◯	◯

Specify

____

Did the patient have a chest x-ray performed?

- ◯ Yes, abnormalities detected
- ◯ Yes, normal
- ◯ No, not performed
- ◯ Don't know
- ◯ Prefer not to answer

If imaging was performed, what type?

	Yes, abnormalities detected	Yes, normal	No, not performed	Don't know	Prefer not to answer
CT Brain	○	○	○	○	○
CT Chest	○	○	○	○	○
CT Abdomen	○	○	○	○	○
Lung Ultrasound	○	○	○	○	○
Vascular Ultrasound	○	○	○	○	○
Abdominal Ultrasound	○	○	○	○	○
Neonatal Ultrasound Brain	○	○	○	○	○
MRI Brain	○	○	○	○	○
MRI Spine	○	○	○	○	○
MRI Abdomen	○	○	○	○	○
Other	○	○	○	○	○

Specify

____

Date of CT Brain

____
(MM/DD/YYYY)

Date of CT Chest

____
(MM/DD/YYYY)

Date of CT Abdomen

____
(MM/DD/YYYY)

Date of Lung Ultrasound

____
(MM/DD/YYYY)

Date of Vascular Ultrasound

____
(MM/DD/YYYY)

Date of Abdomen Ultrasound

____
(MM/DD/YYYY)

Date of Neonatal Ultrasound

____
(MM/DD/YYYY)

Date of MRI Brain

____
(MM/DD/YYYY)

Date of MRI Spine

____
(MM/DD/YYYY)

Date of MRI Abdomen

____
(MM/DD/YYYY)

Date of Other Radiographic Imaging

____
(MM/DD/YYYY)

Treatment

What medications did the patient take or receive to treat Acute COVID-19/MIS-C/Long COVID (PASC)?

	Yes	No	Not reported	Don't know	Prefer not to answer
Anti-coagulant	○	○	○	○	○
Heparin	○	○	○	○	○
Enoxaparin	○	○	○	○	○
Warfarin	○	○	○	○	○
Direct oral anticoagulant (DOAC)	○	○	○	○	○
Antiplatelets/aspirin therapy	○	○	○	○	○
Systemic antibiotics	○	○	○	○	○
Immune modulators/immunosuppressants	○	○	○	○	○
Anakinra	○	○	○	○	○
Tocilizumab	○	○	○	○	○
Convalescent plasma	○	○	○	○	○
SARS-CoV-2 monoclonal antibodies	○	○	○	○	○
Intravenous immunoglobulins (IVIG)	○	○	○	○	○
Interferon	○	○	○	○	○
Tumor necrosis factor (TNF) inhibitors (i.e. infliximab, etanercept, adalimumab)	○	○	○	○	○
NSAID-Ibuprofen	○	○	○	○	○
Anti-viral/anti-COVID	○	○	○	○	○
Diabetic medications	○	○	○	○	○
Systemic steroids	○	○	○	○	○
Other medications	○	○	○	○	○

Specify antibiotic

____

If anti-viral/antibiotic, what type?

	Yes	No	Not reported	Don't know	Prefer not to answer
Remdesivir	◯	◯	◯	◯	◯
Ribavirin	◯	◯	◯	◯	◯

Insulin?

- ◯ Yes
- ◯ No
- ◯ Not reported
- ◯ Don't know
- ◯ Prefer not to answer

If inhaled medications, what type?

	Yes	No	Not reported	Don't know	Prefer not to answer
Inhaled steroids	◯	◯	◯	◯	◯
Albuterol	◯	◯	◯	◯	◯
Ipratropium	◯	◯	◯	◯	◯

If systemic steroids, what type?

	Yes	No	Not reported	Don't know	Prefer not to answer
Dexamethasone	◯	◯	◯	◯	◯
Methylprednisolone	◯	◯	◯	◯	◯
Prednisone/prednisolone	◯	◯	◯	◯	◯
Hydrocortisone	◯	◯	◯	◯	◯
Fludrocortison	◯	◯	◯	◯	◯

Specify

____

Outcomes

Did the patient die?

- ◯ Yes
- ◯ No
- ◯ Not reported
- ◯ Don't know
- ◯ Prefer not to answer

Death due to COVID/MIS-C?

- ◯ Yes
- ◯ No
- ◯ N/A or not reported
- ◯ Don't know
- ◯ Prefer not to answer

Date of Death

____
(MM/DD/YYYY)

If hospitalized for suspected or diagnosed COVID-19 and survived, to where was the participant discharged?

- ◯ Home with in-home nursing care
- ◯ Home with self-care
- ◯ Rehabilitation facility/nursing facility
- ◯ Other
- ◯ N/A or not reported
- ◯ Prefer not to answer

Specify ________________________________

Did the patient receive the following for treatment of COVID in the hospital?

	Yes	No	Not reported	Don't know	Prefer not to answer
Invasive mechanical ventilation (e.g., endotracheal intubation, mechanical ventilation via tracheostomy)	◯	◯	◯	◯	◯
New tracheostomy	◯	◯	◯	◯	◯
Noninvasive mechanism ventilation (e.g. CPAP, BiPAP, NIPPV)	◯	◯	◯	◯	◯
Extracorporeal membrane oxygenation (ECMO)	◯	◯	◯	◯	◯
Vasoactive medications	◯	◯	◯	◯	◯
Arterial catheter placement	◯	◯	◯	◯	◯
Cardiopulmonary resuscitation with/without return of spontaneous circulation	◯	◯	◯	◯	◯
Central venous catheter placement	◯	◯	◯	◯	◯
Low flow oxygen therapy (e.g. nasal cannula, simple mask, face tent)	◯	◯	◯	◯	◯
High flow oxygen therapy	◯	◯	◯	◯	◯
Invasive management of thrombosis (e.g., surgical thrombectomy, endovascular thrombectomy, catheter-directed thrombolysis)	◯	◯	◯	◯	◯
Renal replacement therapy (RRT)	◯	◯	◯	◯	◯
Pacemaker placement	◯	◯	◯	◯	◯
Left ventricular assist device (LVAD)	◯	◯	◯	◯	◯
Other, specify	◯	◯	◯	◯	◯

Specify ________________________________

Is the treatment ongoing?	◯ Yes<br>◯ No<br>◯ N/A (no support)<br>◯ Don't know<br>◯ Prefer not to answer
Date intensive treatment started	______________________<br>(MM/DD/YYYY)
Was there a "Do Not Resuscitate" order or any other limitation of support?	◯ Yes<br>◯ No<br>◯ Don't know<br>◯ Prefer not to answer
Date intensive treatment stopped	______________________<br>(MM/DD/YYYY)
Did your child get breakfast and/or lunch from the school in the 2019-2020 school year before the COVID-19 pandemic?	◯ No<br>◯ Yes<br>◯ Don't know<br>◯ Not applicable/child did not go to school in person<br>◯ Prefer not to answer
Did your child's school continue to provide breakfast and/or lunch during the COVID-19 pandemic (since March 2020)?	◯ No<br>◯ Yes, less frequently<br>◯ Yes, same frequency<br>◯ Yes, more frequently<br>◯ Prefer not to answer
(13+) During your life, how often have you felt that you were treated badly or unfairly because of your race or ethnicity?	◯ Never<br>◯ Rarely<br>◯ Sometimes<br>◯ Most of the time<br>◯ Always<br>◯ Prefer not to answer
(13+) Since the start of the pandemic (since March 2020), have you felt that you were treated badly or unfairly because of your race or ethnicity?	◯ Less<br>◯ Same amount<br>◯ More<br>◯ Not applicable (N/A)<br>◯ Prefer not to answer

(15+) Please answer the following questions on your beliefs about how the coronavirus is affecting people of your race/ethnicity.

	Strongly disagree	Somewhat disagree	Neutral	Somewhat agree	Strongly Agree	Prefer not to answer
(13+) I believe the country has become more dangerous for people in my racial/ethnic group because of fear of the coronavirus.	◯	◯	◯	◯	◯	◯

People of my race/ethnicity are more likely to lose their job because of the coronavirus.	◯	◯	◯	◯	◯	◯
I worry about people thinking I have the coronavirus simply because of my race/ethnicity.	◯	◯	◯	◯	◯	◯
Most social and mass media reports about the coronavirus create bias against people of my racial/ethnic group.	◯	◯	◯	◯	◯	◯
People of my race/ethnicity are more likely to get the coronavirus.	◯	◯	◯	◯	◯	◯
People of my race/ethnicity will not receive coronavirus healthcare as good as the care received by other groups.	◯	◯	◯	◯	◯	◯
Due to the coronavirus, I have been cyberbullied because of my race/ethnicity.	◯	◯	◯	◯	◯	◯
Since the coronavirus, I have seen a lot more cyberbullying of people of my race/ethnicity.	◯	◯	◯	◯	◯	◯
Negative social media posts against people of my race/ethnicity have increased because of the coronavirus.	◯	◯	◯	◯	◯	◯

Is [your/your child's] current school a...

- ◯ A public school, including charter school or magnet school
- ◯ A private school, including private religious schools
- ◯ Bureau of Indian Affairs (BIA) or tribal school
- ◯ Early Childhood Center (school/center includes preschool and/or early elementary grades)
- ◯ Special Education school - primarily serves children with disabilities
- ◯ College, community college, or university
- ◯ Homeschool, including co-ops
- ◯ Full-time cyber school virtual school
- ◯ Prefer not to answer

Does your child have an Individualized Education Plan (IEP) or 504 plan?

- ◯ No
- ◯ Yes
- ◯ Don't know
- ◯ Prefer not to answer

Was your child receiving any specialized services or resources on an Individualized Education Plan (IEP) or 504 plan in the 2019-2020 school year before the pandemic?

- ◯ No
- ◯ Yes
- ◯ Don't know
- ◯ Prefer not to answer

During the pandemic (since March 2020) were your child's services less, the same, or more frequent compared to before the pandemic?	◯ Less<br>◯ Same<br>◯ More<br>◯ N/A<br>◯ Prefer not to answer
From March 2020-May/June 2020, what was the primary form of schooling for your child?	◯ Attend school in person ONLY<br>◯ Attend school remotely ONLY<br>◯ Attend school via a hybrid model that included in person schooling and remote distance learning<br>◯ Not attend school because school was cancelled<br>◯ Not attend school because child dropped out<br>◯ Not attend school for other reason<br>◯ NA<br>◯ Prefer not to answer
Other	______________________________
From Aug/Sept 2020-May/June 2021, what was the primary form of schooling for your child?	◯ Attend school in person ONLY<br>◯ Attend school remotely ONLY<br>◯ Attend school via a hybrid model that included in person schooling and remote distance learning<br>◯ Not attend school because school was cancelled<br>◯ Not attend school because child dropped out<br>◯ Not attend school for other reason<br>◯ NA<br>◯ Prefer not to answer
Other	______________________________
From Aug/Sept 2021-present, what was the primary form of schooling for your child?	◯ Attend school in person ONLY<br>◯ Attend school remotely ONLY<br>◯ Attend school via a hybrid model that included in person schooling and remote distance learning<br>◯ Not attend school because school was cancelled<br>◯ Not attend school because child dropped out<br>◯ Not attend school for other reason<br>◯ NA<br>◯ Prefer not to answer
Other	______________________________
Since start of the current school year (or past school year if on summer break), about how many days did this child miss school (including missing remote learning)?	◯ No missed school days<br>◯ 1-3 days<br>◯ 4-6 days<br>◯ 7-10 days<br>◯ 11-15 days<br>◯ 15 or more days<br>◯ This child was not enrolled in school<br>◯ Don't know<br>◯ Prefer not to answer

About how many days did this child miss school (including missing remote learning) because of illness of you or a family member from COVID-19?

- ◯ No missed school days
- ◯ 1-3 days
- ◯ 4-6 days
- ◯ 7-10 days
- ◯ 11-15 days
- ◯ 15 or more days
- ◯ This child was not enrolled in school
- ◯ Don't know
- ◯ Prefer not to answer

Using a scale of 1-5 where 1 is not at all true, and 5 is completely true, how true would you say each of the following statements is for your child's school/college, regarding how your experiences were/are during the COVID-19 pandemic (since March 2020)

	Not at all true (1)	(2)	(3)	(4)	Completely true (5)	Prefer not to answer
Remote learning is as effective as live/traditional classroom lectures for my child.	◯	◯	◯	◯	◯	◯
My child can meet his/her educational goals with remote learning.	◯	◯	◯	◯	◯	◯
My child has sufficient social interaction with peers during remote learning.	◯	◯	◯	◯	◯	◯
It seems my child experiences a lack of interest during remote learning.	◯	◯	◯	◯	◯	◯
It seems my child experiences frustration during remote learning.	◯	◯	◯	◯	◯	◯

Teacher Relationships (12-18)

	Strongly disagree	Disagree	Neither Agree or Disagree	Agree	Strongly Agree	Prefer not to answer
Teachers understand my problems	◯	◯	◯	◯	◯	◯
Teachers and staff seem to take a real interest in my future	◯	◯	◯	◯	◯	◯
Teachers are available when I need to talk with them	◯	◯	◯	◯	◯	◯
It is easy to talk with teachers	◯	◯	◯	◯	◯	◯
Students get along well with teachers	◯	◯	◯	◯	◯	◯

At my school, there is a teacher or some other adult who notices when I'm not there	◯	◯	◯	◯	◯	◯
Teachers at my school help us children with our problems	◯	◯	◯	◯	◯	◯
My teachers care about me	◯	◯	◯	◯	◯	◯
My teacher makes me feel good about myself	◯	◯	◯	◯	◯	◯

School Connectedness (12-18)

	Strongly Agree	Agree	Neither Agree nor Disagree	Agree	Strongly Agree	Prefer not to answer
My schoolwork is exciting	◯	◯	◯	◯	◯	◯
Students can make suggestions on courses that are offered	◯	◯	◯	◯	◯	◯
Students are publicly recognized for their outstanding performances in speech, drama, art, music, etc.	◯	◯	◯	◯	◯	◯
If this school had an extra period during the day, I would take an additional academic class	◯	◯	◯	◯	◯	◯
This school makes students enthusiastic about learning	◯	◯	◯	◯	◯	◯
Students are frequently rewarded or praised by faculty and staff for following school rules	◯	◯	◯	◯	◯	◯

Academic Support (12-18)

	Strongly Agree	Agree	Neither Agree nor Disagree	Agree	Strongly Agree	Prefer not to answer
I usually understand my homework assignments	◯	◯	◯	◯	◯	◯
Teachers make it clear what work needs to be done to get the grade I want	◯	◯	◯	◯	◯	◯
I believe that teachers expect all students to learn	◯	◯	◯	◯	◯	◯
I feel that I can do well in this school	◯	◯	◯	◯	◯	◯
My teachers believe that I can do well in my school work	◯	◯	◯	◯	◯	◯

I try hard to succeed in my classes	○	○	○	○	○	○

	Strongly Agree	Agree	Neither Agree nor Disagree	Agree	Strongly Agree	Prefer not to answer
Classroom rules are applied equally	○	○	○	○	○	○
Problems in this school are solved by students and staff	○	○	○	○	○	○
Students get in trouble if they do not follow school rules	○	○	○	○	○	○
The rules of the school are fair	○	○	○	○	○	○
School rules are enforced consistently and fairly	○	○	○	○	○	○
My teachers make it clear to me when I have misbehaved in class	○	○	○	○	○	○
Discipline is fair	○	○	○	○	○	○

(Only answer if in-person schooling - 12-18) School Physical Environment

	Strongly Agree	Agree	Neither Agree nor Disagree	Agree	Strongly Agree	Prefer not to answer
The school grounds are kept clean	○	○	○	○	○	○
My school is neat and clean	○	○	○	○	○	○
My school buildings are generally pleasant and well maintained	○	○	○	○	○	○
My school is usually clean and tidy	○	○	○	○	○	○

School Social Environment

	Strongly Agree	Agree	Neither Agree nor Disagree	Agree	Strongly Agree	Prefer not to answer
I am happy with kinds of students who go to my school	○	○	○	○	○	○
I am happy, in general, with the other students who go to my school	○	○	○	○	○	○

Perceived Exclusion/Privilege

	Strongly Agree	Agree	Neither Agree nor Disagree	Agree	Strongly Agree	Prefer not to answer
At my school, the same person always gets to help the teacher	◯	◯	◯	◯	◯	◯
At my school, the same kids get chosen every time to take part in after-school or special activities	◯	◯	◯	◯	◯	◯
The same kids always get to use things, like a computer, a ball or a piano, when we play	◯	◯	◯	◯	◯	◯

Academic Satisfaction

	Strongly Agree	Agree	Neither Agree nor Disagree	Agree	Strongly Agree	Prefer not to answer
I am happy about the number of tests I have	◯	◯	◯	◯	◯	◯
I am happy about the amount of homework I have	◯	◯	◯	◯	◯	◯

In the months before the pandemic (2019-February 2020) how would you describe [you/your child's] grades in school?

◯ Below Average (D's or F's)
◯ Average (C's)
◯ Good (B's)
◯ Very Good (A's and B's)
◯ Excellent (A's)
◯ My child was not graded
◯ Refused
◯ Don't know
◯ Prefer not to answer

During the current school year, how would you describe [you/your child's] grades in school?

◯ Below Average (D's or F's)
◯ Average (C's)
◯ Good (B's)
◯ Very Good (A's and B's)
◯ Excellent (A's)
◯ My child was not graded
◯ Refused
◯ Don't know
◯ Prefer not to answer

How has the COVID-19 outbreak affected your regular childcare/supervision of school aged children (K-12)?

☐ I had difficulty arranging for childcare/supervision
☐ I had to pay more for childcare/supervision
☐ My co-parent or I no longer needed childcare
☐ My co-parent or I had to change our work schedule or quit our job to care for our children
☐ My regular childcare/supervision was not affected by the COVID-19 outbreak
☐ I do not have a school-age child who needed regular supervision
☐ Prefer not to answer

How often is a computer/laptop or other digital device (e.g., tablet) available to your child for educational purposes to support remote distance learning?	◯ Always available<br>◯ Usually available<br>◯ Sometimes available<br>◯ Rarely available<br>◯ Never available<br>◯ N/A<br>◯ Prefer not to answer
Is/are there computer(s) or other digital devices?...	☐ Provided by the child's school or school district to use outside of school<br>☐ Provided by someone in the household or family, or it is the child's<br>☐ Provided by another source<br>☐ N/A<br>☐ Prefer not to answer
How often is the Internet reliable so that your child is able to work remotely for education purposes to support remote virtual learning?	◯ Always available<br>◯ Usually available<br>◯ Sometimes available<br>◯ Rarely available<br>◯ Never available<br>◯ N/A<br>◯ Prefer not to answer
Are internet services...	☐ Paid for by the children's school or school district<br>☐ Paid for by someone in the household or family<br>☐ Paid for by another source<br>☐ N/A<br>☐ Prefer not to answer
Compared to before the COVID-19 outbreak (before March 2020), [do you/does your child] feel	◯ Much less socially connected<br>◯ Less socially connected<br>◯ Slightly less socially connected<br>◯ Slightly more socially connected<br>◯ More socially connected<br>◯ Much more socially connected<br>◯ Prefer not to answer

(8-17) In the past month, please describe how often...

	Never	Rarely	Sometimes	Usually	Always	Prefer not to answer
I have someone who understands my problems	◯	◯	◯	◯	◯	◯
I have someone who will listen to me when I need to talk	◯	◯	◯	◯	◯	◯
I have someone to talk with when I have a bad day	◯	◯	◯	◯	◯	◯
There is someone around to help me if I need it	◯	◯	◯	◯	◯	◯

I can get helpful advice from others when dealing with a problem	○	○	○	○	○	○
I get useful advice about important things in my life	○	○	○	○	○	○
I have someone to talk with about school problems	○	○	○	○	○	○

(8-17) Compared to before the COVID-19 pandemic (before March 2020)...

	Less	The same	More	Prefer not to answer
I have someone who understands my problems	○	○	○	○
I have someone who will listen to me when I need to talk	○	○	○	○
I have someone to talk with when I have a bad day	○	○	○	○
There is someone around to help me if I need it	○	○	○	○
I can get helpful advice from others when dealing with a problem	○	○	○	○
I get useful advice about important things in my life	○	○	○	○
I have someone to talk with about school problems	○	○	○	○

(1-5) In the past 7 days

	Never	Rarely	Sometimes	Usually	Always	Prefer not to answer
My child shared with other kids	○	○	○	○	○	○
My child played well with other children	○	○	○	○	○	○
My child laughed and smiled with other children	○	○	○	○	○	○
My child showed interest in other children	○	○	○	○	○	○

(1-5) Compared to before the COVID-19 pandemic (before March 2020)...

	Less	The same	More	Prefer not to answer
My child shares with other kids	○	○	○	○
My child plays well with other children	○	○	○	○
My child laughs and smiles with other children	○	○	○	○

My child shows interest in other children	○	○	○	○

(5-17) In the past 7 days...

	Never	Rarely	Sometimes	Usually	Always	Prefer not to answer
My child felt accepted by other kids his/her age	○	○	○	○	○	○
My child was able to count on his/her friends	○	○	○	○	○	○
My child was good at making friends	○	○	○	○	○	○
My child and his/her friends helped each other out	○	○	○	○	○	○
Other kids wanted to be my child's friend	○	○	○	○	○	○
Other kids wanted to be with my child	○	○	○	○	○	○
Other kids wanted to talk to my child	○	○	○	○	○	○

(5-17) Compared to before the COVID-19 pandemic (before March 2020)...

	Less	The same	More	Prefer not to answer
My child feels accepted by other kids their age	○	○	○	○
My child is good at making friends	○	○	○	○
My child and his/her friends help each other out	○	○	○	○
Other kids want to be my child's friend	○	○	○	○
Other kids want to be with my child	○	○	○	○
Other kids wants to talk to my child	○	○	○	○

(8-17) In the past 7 days...

	Never	Rarely	Sometimes	Usually	Always	Prefer not to answer
I felt accepted by other kids my age	○	○	○	○	○	○
I was able to count on friends	○	○	○	○	○	○
I was able to talk about everything with my friends	○	○	○	○	○	○
I was good at making friends	○	○	○	○	○	○

My friends and I helped each other out	○	○	○	○	○	○
Other kids wanted to be my friend	○	○	○	○	○	○
Other kids wanted to be with me	○	○	○	○	○	○
Other kids wanted to talk to me	○	○	○	○	○	○

(8-17) Compared to before the COVID-19 pandemic (before March 2020)...

	Less	The same	More	Prefer not to answer
I feel accepted by other kids my age	○	○	○	○
I am able to count on my friends	○	○	○	○
I am able to talk about everything with my friends	○	○	○	○
I am good at making friends	○	○	○	○
My friends and I help each other out	○	○	○	○
Other kids want to be my friend	○	○	○	○
Other kids want to be with me	○	○	○	○
Other kids want to talk to me	○	○	○	○

How [have/were] YOU and your parent(s) (been) getting along during the COVID-19 outbreak (since March 2020)?

- ○ Very well - no problems or tension
- ○ Well - occasional tension, some tension, but manageable
- ○ Okay - some tension and sometimes things get out of hand (a few heated arguments)
- ○ Not very well - tense, lots of arguing, unsettled feeling, definite problems
- ○ Terribly
- ○ Prefer not to answer

Is this a change from how you were getting along during the COVID-19 outbreak (since March 2020)?

- ○ Yes
- ○ No
- ○ Prefer not to answer

(13+) During the COVID-19 outbreak (since March 2020), did things ever get to the point where an adult you were living with got physically violent with a child (for example, shoved, hit, kicked, or shook [her/him/them])?

- ○ Yes
- ○ No
- ○ Prefer not to answer

(13+) During the COVID-19 outbreak (since March 2020), was an adult in your household ever physically violent with you (for example, shoved, hit, kicked, or shook you)?

- ○ Yes
- ○ No
- ○ Prefer not to answer

(13+) During the COVID-19 outbreak (since March 2020), did things ever get to the point where an adult you were living with got physically violent with someone else (for example, shoved, hit, kicked, or shook someone else)?

- ○ Yes
- ○ No
- ○ Prefer not to answer

Social Media/Screen Time

(13+) ON MOST WEEKDAYS, about how much time did [you/your child] spend in front of a TV, computer, cellphone, or other electronic device watching programs, playing games, accessing the Internet or using social media? Do not include time spent doing schoolwork.

- ◯ Less than 1 hour
- ◯ 1 hour
- ◯ 2 hours
- ◯ 3 hours
- ◯ 4 or more hours
- ◯ Prefer not to answer

Compared to before the COVID-19 outbreak (before March 2020), how much are you now of the following?

	Less	Same amount	More	Prefer not to answer
Spending time watching TV/videos (such as YouTube), playing video/computer games, or using social media for educational purposes, including schoolwork	◯	◯	◯	◯
Spending time watching TV/videos (such as YouTube), playing video/computer games, or using social media for non-educational purposes	◯	◯	◯	◯

(13+) Since becoming aware of the COVID-19 outbreak, how often have you felt happy and satisfied with your life?

- ◯ Not at all
- ◯ Rarely
- ◯ Sometimes
- ◯ Often
- ◯ Very often
- ◯ Prefer not to answer

	Not at all	A little bit	Somewhat	Quite a bit	Very much	Prefer not to answer
(8-17) I felt attentive	◯	◯	◯	◯	◯	◯
(8-12) I felt delighted/(3-12) My child was delighted	◯	◯	◯	◯	◯	◯
(8-12) I felt calm	◯	◯	◯	◯	◯	◯
(13-17) I felt interested/(3-12) My child was interested	◯	◯	◯	◯	◯	◯
(8-12) I felt confident/(3-12) My child was confident	◯	◯	◯	◯	◯	◯
(8-12) I felt energetic	◯	◯	◯	◯	◯	◯
(8-12) I felt able to concentrate	◯	◯	◯	◯	◯	◯
(13-17) I felt cheerful/(3-12) My child was cheerful	◯	◯	◯	◯	◯	◯
(13-17) I felt joyful/(3-12) My child was joyful	◯	◯	◯	◯	◯	◯

(13-17) I felt at ease/(3-12) My child was at ease	○	○	○	○	○	○
(13-17) I felt peaceful	○	○	○	○	○	○
(13-17) I felt good-natured	○	○	○	○	○	○
(13-17) I felt content	○	○	○	○	○	○
(3-12) My child was inspired	○	○	○	○	○	○
(3-12) My child was happy	○	○	○	○	○	○
(3-12) My child was alert	○	○	○	○	○	○
(8-12) I felt enthusiastic/(3-12) My child was enthusiastic	○	○	○	○	○	○

(13+) What have you done to cope with your stress related to the COVID-19 outbreak? (Mark all that apply)/(Child 8+) Which of the following strategies [have been/were] helpful to YOUR CHILD while staying at home because of the COVID-19 outbreak? (Mark all that apply)

- ☐ Arts and crafts projects
- ☐ Cooking/baking
- ☐ Drinking alcohol (13+)
- ☐ Engaging in more family activities (e.g., games, sports)
- ☐ Exercising/walking
- ☐ Increasing time reading books, or doing activities like puzzles and crosswords
- ☐ Meditation and/or mindfulness practices
- ☐ Spiritual/religious practices
- ☐ Talking to healthcare providers more frequently, including mental healthcare providers (e.g., therapists, psychologists, counselors)
- ☐ Texting, calling or video-calling family members or friends
- ☐ Using tobacco (e.g., smoking), using marijuana (e.g., smoking, edibles), vaping (13+)
- ☐ Volunteer work
- ☐ (I have not/My child has) done any of these things to cope with the COVID-19 outbreak
- ☐ Other
- ☐ Prefer not to answer

Specify ______________________________

Is your life lonelier because of the COVID-19 pandemic?

- ○ Yes
- ○ No
- ○ Prefer not to answer

(9+) During the COVID-19 pandemic (since March 2020)

	Not at all	Slightly	Moderately	Very	Extremely	Prefer not to answer
How worried have you been about coronavirus (COVID-19)?	○	○	○	○	○	○
How worried have others around you been about coronavirus (COVID-19)?	○	○	○	○	○	○

How worried have you been about changes to schooling (e.g., missing school in-person)?	○	○	○	○	○	○
How much do you think your life has changed due to coronavirus (COVID-19)?	○	○	○	○	○	○
How hopeful have you been that the coronavirus/COVID- 19 crisis in your area will end soon?	○	○	○	○	○	○

In the past week:

	Not at all	Slightly	Moderately	Very	Extremely	Prefer not to answer
How worried have you been about coronavirus (COVID-19)?	○	○	○	○	○	○
How worried have others around you been about coronavirus (COVID-19)?	○	○	○	○	○	○
How worried have you been about changes to schooling (e.g., missing school in-person)?	○	○	○	○	○	○
How much do you think your life has changed due to coronavirus (COVID-19)?	○	○	○	○	○	○
How hopeful have you been that the coronavirus/COVID-19 crisis in your area will end soon?	○	○	○	○	○	○

(13+) During the COVID-19 pandemic (since March 2020), how often did you:

	Not at all	Rarely	Sometimes	Often	Very often	Prefer not to answer
Have difficulty sleeping	○	○	○	○	○	○
Startle easily	○	○	○	○	○	○
Have angry outbursts	○	○	○	○	○	○
Feel a sense of time slowing down	○	○	○	○	○	○
Feel in a daze	○	○	○	○	○	○
Try to avoid thoughts and feelings about COVID-19	○	○	○	○	○	○
Have distressing dreams about COVID-19	○	○	○	○	○	○
Feel distressed when you saw something that reminded you of COVID-19	○	○	○	○	○	○

During the past week, on how many days did this child exercise, play a sport, or participate in physical activity (including physical education classes) for at least 60 minutes?	◯ 0 days<br>◯ 1-3 days<br>◯ 4-6 days<br>◯ Every day<br>◯ Prefer not to answer
How has this changed compared to before the COVID-19 outbreak (before March 2020)?	◯ Fewer days<br>◯ Same number of days<br>◯ More days<br>◯ Don't know<br>◯ Prefer not to answer
(Child 13+) How has your quality of sleep changed compared to before the COVID-19 outbreak (before March 2020)?	◯ It's gotten a lot worse<br>◯ It's gotten a little worse<br>◯ Stayed the same<br>◯ It's gotten a little better<br>◯ It's gotten a lot better<br>◯ Prefer not to answer
In the last week, how many hours of sleep did you get on most nights?	◯ More than 11 hours<br>◯ 9-11 hours<br>◯ 8-9 hours<br>◯ 7-8 hours<br>◯ 5-7 hours<br>◯ Less than 5 hours<br>◯ Don't know<br>◯ Prefer not to answer
How has this changed compared to before the COVID-19 outbreak (before March 2020)?	◯ Less time<br>◯ Same time<br>◯ More time<br>◯ Don't know<br>◯ Prefer not to answer
In the last week, how many hours of sleep did your child get on most nights?	◯ More than 11 hours<br>◯ 9-11 hours<br>◯ 8-9 hours<br>◯ 7-8 hours<br>◯ 5-7 hours<br>◯ Less than 5 hours<br>◯ Don't know<br>◯ Prefer not to answer
How has this changed compared to before the COVID-19 outbreak (before March 2020)?	◯ Less time<br>◯ Same time<br>◯ More time<br>◯ Don't know<br>◯ Prefer not to answer

	Yes	No	Don't know	Prefer not to answer
Tourette Syndrome	◯	◯	◯	◯
Depression	◯	◯	◯	◯
Anxiety problems	◯	◯	◯	◯

Autism or Autism Spectrum Disorder (ASD), Asperger's Disorder, Pervasive Developmental Disorder (PDD)	◯	◯	◯	◯
Attention Deficit Disorder or Attention Deficit Hyperactive Disorder (ADD/ADHD)	◯	◯	◯	◯
Chronic fatigue	◯	◯	◯	◯
Post-traumatic stress disorder (PTSD)	◯	◯	◯	◯
Suicidal thoughts or behaviors	◯	◯	◯	◯
Mania or bipolar disorder	◯	◯	◯	◯
Behavioral disorder or conduct problems	◯	◯	◯	◯
Developmental delay	◯	◯	◯	◯
Intellectual disability	◯	◯	◯	◯
Speech or other language disorder	◯	◯	◯	◯
Learning disability	◯	◯	◯	◯

Specify which conditions the child currently has. ______________________________

(8+) In general, how would you rate your physical health?/In general, how is your child's physical health?

◯ Excellent
◯ Very good
◯ Good
◯ Fair
◯ Poor
◯ Prefer not to answer

(8+) In general, how would you rate your mental health, including how you feel, think, and behave?/In general, how is your child's mental or emotional health?

◯ Excellent
◯ Very good
◯ Good
◯ Fair
◯ Poor
◯ Prefer not to answer

During the past TWO (2) WEEKS, how much (or how often) has your child.../ (11-17) During the past TWO (2) WEEKS, how much (or how often) have you...

	None- Not at all	Slight - Rare- less than a day or two	Mild- several days	Moderate- More than half the days	Severe- Nearly every day	Prefer not to answer

Complained of stomach aches, headaches, or other aches and pains? /(11-17) Been bothered by stomach aches, headaches, or other aches and pains?	○	○	○	○	○	○
Said he/she was worried about his/her health or about getting sick? /(11-17) Worried about your health or about getting sick?	○	○	○	○	○	○
Had problems sleeping-that is, trouble falling asleep, staying asleep, or waking up too early? /(11-17) Been bothered by not being able to fall asleep or stay asleep, or by waking up too early?	○	○	○	○	○	○
Had problems paying attention when he/she was in class or doing his/her homework or reading a book or playing a game /(11-17) Been bothered by not being able to pay attention when you were in class or doing homework or reading a book or playing a game?	○	○	○	○	○	○
Had less fun doing things than he/she used to? /(11-17) Had less fun doing things than you used to?	○	○	○	○	○	○
Seemed sad or depressed for several hours? /(11-17) Felt sad or depressed for several hours?	○	○	○	○	○	○
Seemed more irritated or easily annoyed than usual? /(11-17) Felt more irritated or easily annoyed than usual?	○	○	○	○	○	○
Seemed angry or lost his/her temper? /(11-17) Felt angry or lost your temper?	○	○	○	○	○	○
Started lots more projects than usual or did more risky things than usual? /(11-17) Started lots more projects than usual or done more risky things than usual?	○	○	○	○	○	○

Slept less than usual for him/her, but still had lots of energy? /(11-17) Slept less than usual but still had a lot of energy?	○	○	○	○	○	○
Said he/she felt nervous, anxious, or scared? /(11-17) Felt nervous, anxious, or scared?	○	○	○	○	○	○
Not been able to stop worrying? /(11-17) Not been able to stop worrying?	○	○	○	○	○	○
Said he/she couldn't do things he/she wanted to or should have done, because they made him/her feel nervous? /(11-17) Not been able to do things you wanted to or should have done, because they made you feel nervous?	○	○	○	○	○	○
Said that he/she heard voices-when there was no one there-speaking about him/her or telling him/her what to do or saying bad things to him/her? /(11-17) Heard voices-when there was no one there-speaking about you or telling you what to do or saying bad things to you?	○	○	○	○	○	○
Said that he/she had a vision when he/she was completely awake-that is, saw something or someone that no one else could see? /(11-17) Had visions when you were completely awake-that is, seen something or someone that no one else could see?	○	○	○	○	○	○
Said that he/she had thoughts that kept coming into his/her mind that he/she would do something bad or that something bad would happen to him/her or to someone else? /(11-17) Had thoughts that kept coming into your mind that you would do something bad or that something bad would happen to you or to someone else?	○	○	○	○	○	○

Said he/she felt the need to check on certain things over and over again, like whether a door was locked or whether the stove was turned off? /(11-17) Felt the need to check on certain things over and over again, like whether a door was locked or whether the stove was turned off?	○	○	○	○	○	○
Seemed to worry a lot about things he/she touched being dirty or having germs or being poisoned? /(11-17) Worried a lot about things you touched being dirty or having germs or being poisoned?	○	○	○	○	○	○
Said that he/she had to do things in a certain way, like counting or saying special things out loud, in order to keep something bad from happening? /(11-17) Felt you had to do things in a certain way, like counting or saying special things, to keep something bad from happening?	○	○	○	○	○	○

In the past TWO (2) WEEKS, has your child ... (11-17) In the past TWO (2) WEEKS, have you...

	Yes	Don't know	Prefer not to answer
Had an alcoholic beverage (beer, wine, liquor, etc.)? /(11-17) Had an alcoholic beverage (beer, wine, liquor, etc.)?	○	○	○
Smoked marijuana, a cigarette, a cigar, a pipe, e-cigarettes, vaped, or used snuff or chewing tobacco? /(11-17) Smoked marijuana, a cigarette, a cigar, or pipe, e-cigarettes, vaped, or used snuff or chewing tobacco?	○	○	○

Used drugs like cocaine or crack, club drugs (like ecstasy), hallucinogens (like LSD), heroin, inhalants or solvents (like glue), or methamphetamine (like speed)? /(11-17) Used drugs like cocaine or crack, club drugs (like Ecstasy), hallucinogens (like LSD), heroin, inhalants or solvents (like glue), or methamphetamine (like speed)?	○	○	○
Used any medicine without a doctor's prescription (e.g., painkillers [like Vicodin], stimulants [like Ritalin or Adderall], sedatives or tranquilizers [like sleeping pills or Valium], or steroids)? /(11-17) Used any medicine without a doctor's prescription to get high or change the way you feel (e.g., painkillers [like Vicodin], stimulants [like Ritalin or Adderall], sedatives or tranquilizers [like sleeping pills or Valium], or steroids)?	○	○	○
In the past TWO (2) WEEKS, has he/she talked about wanting to kill himself/herself or about wanting to commit suicide? /(11-17) In the last 2 weeks, have you thought about killing yourself or committing suicide?	○	○	○
Has he/she EVER tried to kill himself/herself? /(11-17) Have you EVER tried to kill yourself?	○	○	○

PROMIS Anxiety-In the past 7 days

	Never	Almost Never	Sometimes	Often	Almost Always	Prefer not to answer
My child felt nervous /(8-17) I felt nervous	○	○	○	○	○	○
My child felt scared /(8-17) I felt nervous	○	○	○	○	○	○

My child felt worried /(8-17) I felt worried	◯	◯	◯	◯	◯	◯
My child felt like something awful might happen /(8-17) I felt like something awful might happen	◯	◯	◯	◯	◯	◯
My child worried when he/she was at home /(8-17) I worried when I was at home	◯	◯	◯	◯	◯	◯
My child got scared really easy /(8-17) I got scared really easy	◯	◯	◯	◯	◯	◯
My child worried what could happen to him/her /(8-17) I worried what could happen to me	◯	◯	◯	◯	◯	◯
My child worried when he/she went to bed at night /(8-17) I worried when I went to bed at night	◯	◯	◯	◯	◯	◯

PROMIS Depressive Symptoms-In the past 7 days

	Never	Almost Never	Sometimes	Often	Almost Always	Prefer not to answer
My child could not stop feeling sad /(8-17) I could not stop feeling sad	◯	◯	◯	◯	◯	◯
(8-17) I felt alone	◯	◯	◯	◯	◯	◯
My child felt everything in his/her life went wrong /(8-17) I felt everything in my life went wrong	◯	◯	◯	◯	◯	◯
My child felt like he/she couldn't do anything right /(8-17) I felt like I couldn't do anything right	◯	◯	◯	◯	◯	◯
My child felt lonely /(8-17) I felt lonely	◯	◯	◯	◯	◯	◯
My child felt sad /(8-17) I felt sad	◯	◯	◯	◯	◯	◯
(8-17) I felt unhappy	◯	◯	◯	◯	◯	◯

	Never	Almost Never	Sometimes	Often	Almost Always	Prefer not to answer
It was hard for my child to have fun /(8-17) It was hard for me to have fun	◯	◯	◯	◯	◯	◯

PROMIS Fatigue-In the past 7 days

	Never	Almost Never	Sometimes	Often	Almost Always	Prefer not to answer
Being tired made it hard for my child to play or go out with friends as much as he/she would like /(8-17) Being tired made it hard for me to play or go out with my friends as much as I'd like	○	○	○	○	○	○
My child felt weak /(8-17) I felt weak	○	○	○	○	○	○
My child got tired easily /(8-17) I got tired easily	○	○	○	○	○	○
Being tired made it hard for my child to keep up with schoolwork /(8-17) Being tired made it hard for me to keep up with my schoolwork	○	○	○	○	○	○
My child had trouble finishing things because he/she was too tired /(8-17) I had trouble finishing things because I was too tired	○	○	○	○	○	○
My child had trouble starting things because he/she was too tired /(8-17) I had trouble starting things because I was too tired	○	○	○	○	○	○
My child was so tired it was hard for him/her to pay attention /(8-17) I was so tired it was hard for me to pay attention	○	○	○	○	○	○
My child was too tired to do sports or exercise /(8-17) I was too tired to do sports or exercise	○	○	○	○	○	○
My child was too tired to do things outside /(8-17) I was too tired to do things outside	○	○	○	○	○	○
My child was too tired to enjoy the things he/she likes to do /(8-17) I was too tired to enjoy the things I like to do	○	○	○	○	○	○

RCADS Anxiety and Depression Scale

	Never	Sometimes	Often	Always	Prefer not to answer
My child worries about things /(8-18) I worry about things	◯	◯	◯	◯	◯
My child feels sad or empty /(8-18) I feel sad or empty	◯	◯	◯	◯	◯
When my child has a problem, he/she gets a funny feeling in his/her stomach /(8-18) When I have a problem, I get a funny feeling in my stomach	◯	◯	◯	◯	◯
My child worries when he/she thinks she has done poorly at something /(8-18) I worry when I think I have done poorly at something	◯	◯	◯	◯	◯
My child feels afraid of being alone at home /(8-18) I would feel afraid of being on my own at home	◯	◯	◯	◯	◯
Nothing is much fun for my child anymore /(8-18) Nothing is much fun anymore	◯	◯	◯	◯	◯
My child feels scared when taking a test /(8-18) I feel scared when I have to take a test	◯	◯	◯	◯	◯
My child worries when he/she thinks someone is angry with him/her /(8-18) I feel worried when I think someone is angry with me	◯	◯	◯	◯	◯
My child worries about being away from me /(8-18) I worry about being away from my parents	◯	◯	◯	◯	◯
My child is bothered by bad or silly thoughts or pictures in his/her mind /(8-18) I get bothered by bad or silly thoughts or pictures in my mind	◯	◯	◯	◯	◯
My child has trouble sleeping /(8-18) I have trouble sleeping	◯	◯	◯	◯	◯

My child worries about doing badly at schoolwork /(8-18) I worry that I will do badly at my schoolwork	○	○	○	○	○
My child worries that something awful happen to someone in the family /(8-18) I worry that something awful will happen to someone in my family	○	○	○	○	○
My child suddenly feels as if he/she can't breathe when there is no reason for this /(8-18) I suddenly feel as if I can't breathe when there is no reason for this	○	○	○	○	○
My child has problems with his/her appetite /(8-18) I have problems with my appetite	○	○	○	○	○
My child has to keep checking that she has done things right (like the switch off, or the door is locked) /(8-18) I have to keep checking that I have done things right (like the switch is off, or the door is locked)	○	○	○	○	○
My child feels scared to sleep on his/her own /(8-18) I feel scared if I have to sleep on my own	○	○	○	○	○
My child has trouble going to school the mornings because of feeling nervous or afraid /(8-18) I have trouble going to school in the mornings because I feel nervous or afraid	○	○	○	○	○
My child has no energy for things /(8-18) I have no energy for things	○	○	○	○	○
My child worries about looking foolish /(8-18) I worry I might look foolish	○	○	○	○	○
My child is tired a lot /(8-18) I am tired a lot	○	○	○	○	○

My child worries that bad things will happen to him/her /(8-18) I worry that bad things will happen to me	◯	◯	◯	◯	◯
My child can't seem to get bad or silly thoughts out of his/her head /(8-18) I can't seem to get bad or silly thoughts out of my head	◯	◯	◯	◯	◯
(8-18) When I have a problem, my heart beats really fast	◯	◯	◯	◯	◯
(8-18) I cannot think clearly	◯	◯	◯	◯	◯
(8-18) I suddenly start to tremble or shake when there is no reason for this	◯	◯	◯	◯	◯
(8-18) I worry that something bad will happen to me	◯	◯	◯	◯	◯
(8-18) When I have a problem, I feel shaky	◯	◯	◯	◯	◯
(8-18) I feel worthless	◯	◯	◯	◯	◯
(8-18) I worry about making mistakes	◯	◯	◯	◯	◯
(8-18) I have to think of special thoughts (like numbers or words) to stop bad things from happening	◯	◯	◯	◯	◯
(8-18) I worry what other people think of me	◯	◯	◯	◯	◯
(8-18) I am afraid of being in crowded places (like shopping centers, the movies, buses, busy playgrounds)	◯	◯	◯	◯	◯
(8-18) All of a sudden, I feel really scared for no reason at all	◯	◯	◯	◯	◯
(8-18) I worry about what is going to happen	◯	◯	◯	◯	◯
(8-18) I suddenly become dizzy or faint when there is no reason for this	◯	◯	◯	◯	◯
(8-18) I think about death	◯	◯	◯	◯	◯
(8-18) I feel afraid if I have to talk in front of my class	◯	◯	◯	◯	◯

(8-18) My heart suddenly starts to beat too quickly for no reason	○	○	○	○	○
(8-18) I feel like I don't want to move	○	○	○	○	○
(8-18) I worry that I will suddenly get a scared feeling when there is nothing to be afraid of	○	○	○	○	○
(8-18) I have to do some things over and over again (like washing my hands, cleaning or putting things in a certain order)	○	○	○	○	○
(8-18) I feel afraid that I will make a fool of myself in front of people	○	○	○	○	○
(8-18) I have to do some things in just the right way to stop bad things from happening	○	○	○	○	○
(8-18) I worry when I go to bed at night	○	○	○	○	○
(8-18) I would feel scared if I had to stay away from home overnight	○	○	○	○	○
(8-18) I feel restless	○	○	○	○	○

PROMIS Pain Interference (in the past 7 days)

	Never	Almost Never	Sometimes	Often	Almost Always	Prefer not to answer
My child had trouble sleeping when he/she had pain /(8-17) I had trouble sleeping when I had pain	○	○	○	○	○	○
My child felt angry when he/she had pain /(8-17) I felt angry when I had pain	○	○	○	○	○	○
My child had trouble doing schoolwork when he/she had pain /(8-17) I had trouble doing schoolwork when I had pain	○	○	○	○	○	○
It was hard for my child to pay attention when he/she had pain /(8-17) It was hard for me to pay attention when I had pain	○	○	○	○	○	○

It was hard for my child to run when he/she had pain /(8-17) It was hard for me to run when I had pain	○	○	○	○	○	○
It was hard for my child to walk when he/she had pain /(8-17) It was hard for me to walk one block when I had pain	○	○	○	○	○	○
It was hard for my child to have fun when he/she had pain /(8-17) It was hard to have fun when I had pain	○	○	○	○	○	○
It was hard for my child to stay standing when he/she had pain /(8-17) It was hard to stay standing when I had pain	○	○	○	○	○	○

PROMIS Cognitive Function (in the past four weeks)

	None of the time	A little of the time	Some of the time	Most of the time	All of the time	Prefer not to answer
Your child has to use written lists more often than other people his/her age so he/she will not forget things /(8-17) I have to use written lists more often than other people my age so I will not forget things	○	○	○	○	○	○
It is hard for your child to pay attention to one thing for more than 5-10 minutes /(8-17) It is hard for me to pay attention to one thing for more than 5-10 minutes	○	○	○	○	○	○
Your child has trouble keeping track of what he/she is doing if he/she gets interrupted /(8-17) I have trouble keeping track of what I am doing if I get interrupted	○	○	○	○	○	○
Your child has to read things several times to understand them /(8-17) I have to read things several times to understand them	○	○	○	○	○	○

Your child forgets things easily /(8-17) I forget things easily	◯	◯	◯	◯	◯	◯
Your child has to work really hard to pay attention or he/she makes mistakes /(8-17) I have to work really hard to pay attention or I make mistakes	◯	◯	◯	◯	◯	◯
Your child has trouble remembering to do things like school projects or chores /(8-17) I have trouble remembering to do things like school projects or chores	◯	◯	◯	◯	◯	◯

Below is a list of comments made by people after stressful life events. Please tick each item showing how frequently these comments were true for you during the past seven days. If they did not occur during that time, please tick the 'not at all' box.

	Not at all	Rarely	Sometimes	Often	Prefer not to answer
Do you think about it even when you don't mean to?	◯	◯	◯	◯	◯

Do you try to remove it from your memory?
- ◯ Not at all
- ◯ Rarely
- ◯ Sometimes
- ◯ Often
- ◯ Prefer not to answer

Do you have waves of strong feelings about it?
- ◯ Not at all
- ◯ Rarely
- ◯ Sometimes
- ◯ Often
- ◯ Prefer not to answer

Do you stay away from reminders of it (e.g. places or situations?)
- ◯ Not at all
- ◯ Rarely
- ◯ Sometimes
- ◯ Often
- ◯ Prefer not to answer

Do you try not to talk about it?
- ◯ Not at all
- ◯ Rarely
- ◯ Sometimes
- ◯ Often
- ◯ Prefer not to answer

Do pictures about it pop in your mind?
- ◯ Not at all
- ◯ Rarely
- ◯ Sometimes
- ◯ Often
- ◯ Prefer not to answer

Do other things keep making you think about it?	◯ Not at all<br>◯ Rarely<br>◯ Sometimes<br>◯ Often<br>◯ Prefer not to answer
Do you try not to think about it?	◯ Not at all<br>◯ Rarely<br>◯ Sometimes<br>◯ Often<br>◯ Prefer not to answer
If yes, what change occurred?	◯ Loss of this child's health insurance<br>◯ Fewer benefits / less coverage from the insurance<br>◯ Gaining of insurance, for example as part of emergency coverage of Medicaid expansion<br>◯ Prefer not to answer
Since the start of the COVID-19 pandemic (since March 2020), was there any time when this child needed health care, but it was not received? By health care, we mean medical care as well as other kinds of care like dental care, vision care, and mental health services	◯ No<br>◯ Yes<br>◯ Prefer not to answer
If yes, which types of care were not received?	☐ Medical Care<br>☐ Dental Care<br>☐ Vision Care<br>☐ Hearing Care<br>☐ Mental Health<br>☐ Other (Specify)<br>☐ Prefer not to answer
Specify other type of care not received	__________________________________
Please rate how much the coronavirus pandemic has changed your family's life with respect to Medical health care access	◯ No change<br>◯ Appointments moved to telehealth<br>◯ Delays or cancellations in appointments and/or delays in getting prescriptions or regular vaccinations (e.g., MMR); changes have minimal impact on health<br>◯ Unable to access needed care resulting in severe risk and/or significant impact<br>◯ Prefer not to answer
Please rate how much the coronavirus pandemic has changed your family's life with respect to Mental health treatment access	◯ No change<br>◯ Appointments moved to telehealth<br>◯ Delays or cancellations in appointments and/or delays in getting prescriptions; changes have minimal impact on health<br>◯ Unable to access needed care resulting in severe risk and/or significant impact<br>◯ Prefer not to answer

Since the start of the COVID-19 pandemic (since March 2020), has this child received any treatment or counseling from a mental health professional? Mental health professionals include psychiatrists, psychologists, psychiatric nurses, and clinical social workers.	◯ No<br>◯ Yes<br>◯ Prefer not to answer
Since the start of the COVID-19 pandemic (since March 2020), has this child taken any medication because of difficulties with their emotions, concentration, or behavior?	◯ No<br>◯ Yes<br>◯ Prefer not to answer
Has this child EVER received special services to meet their developmental needs such as speech, occupational, or behavioral therapy?	◯ No<br>◯ Yes<br>◯ Prefer not to answer
Is/was this child receiving these special services during the pandemic (since March 2020)?	◯ No<br>◯ Yes<br>◯ Prefer not to answer
Was this child receiving these special services BEFORE the pandemic (before March 2020)?	◯ No<br>◯ Yes<br>◯ Prefer not to answer
If < 2 years of age, what was the participant's gestational age at birth (in weeks)?	__________________________________

Disability Status

(0-12+) Does this child have deafness or problems with hearing? / (15+) Are you deaf, or do you have serious difficulty hearing?	◯ No<br>◯ Yes<br>◯ Prefer not to answer
(0-12+) Does this child have blindness or problems with seeing even when wearing glasses? / (15+) Are you blind, or do you have serious difficulty seeing, even when wearing glasses?	◯ No<br>◯ Yes<br>◯ Prefer not to answer
(6-12+) Does this child have serious difficulty concentrating, remembering, or making decisions because of a physical, mental, or emotional condition? / (5+) Because of a physical, mental, or emotional condition, do you have serious difficulty concentrating, remembering, or making decisions?	◯ No<br>◯ Yes<br>◯ Prefer not to answer
(6-12+) Does this child have serious difficulty walking or climbing stairs? / (5+) Do you have serious difficulty walking or climbing stairs?	◯ No<br>◯ Yes<br>◯ Prefer not to answer
(6-12+) Does this child have difficulty dressing or bathing? / (5+) Do you have difficulty dressing or bathing?	◯ No<br>◯ Yes<br>◯ Prefer not to answer

(12+) Does this child have difficulty doing errands alone, such as visiting a doctor's office or shopping, because of a physical mental, or emotional condition? / (15+) Because of a physical, mental, or emotional condition, do you have difficulty doing errands alone such as visiting a doctor's office or shopping?	◯ No<br>◯ Yes<br>◯ Prefer not to answer

Special Healthcare needs

Does your child currently need or use medicine prescribed by a doctor (other than vitamins)?	◯ No<br>◯ Yes<br>◯ Prefer not to answer
Is this because of ANY medical, behavioral or other health condition?	◯ No<br>◯ Yes<br>◯ Prefer not to answer
Is this a condition that has lasted or is expected to last for at least 12 months?	◯ No<br>◯ Yes<br>◯ Prefer not to answer
Does your child need or use more medical care, mental health or educational services than is usual for most children of the same age?	◯ No<br>◯ Yes<br>◯ Prefer not to answer
Is this because of ANY medical, behavioral or other health condition?	◯ No<br>◯ Yes<br>◯ Prefer not to answer
Is this a condition that has lasted or is expected to last for at least 12 months?	◯ No<br>◯ Yes<br>◯ Prefer not to answer
Is your child limited or prevented in any way in his or her ability to do the things most children of the same age can do?	◯ No<br>◯ Yes<br>◯ Prefer not to answer
Is this because of ANY medical, behavioral or other health condition?	◯ No<br>◯ Yes<br>◯ Prefer not to answer
Is this a condition that has lasted or is expected to last for at least 12 months?	◯ No<br>◯ Yes<br>◯ Prefer not to answer
Does your child need or get special therapy, such as physical, occupational or speech therapy?	◯ No<br>◯ Yes<br>◯ Prefer not to answer
Is this because of ANY medical, behavioral or other health condition?	◯ No<br>◯ Yes<br>◯ Prefer not to answer
Is this a condition that has lasted or is expected to last for at least 12 months?	◯ No<br>◯ Yes<br>◯ Prefer not to answer

Does your child have any kind of emotional, developmental or behavioral problem for which he or she needs or gets treatment or counseling?	◯ No<br>◯ Yes<br>◯ Prefer not to answer
Has this problem lasted or is it expected to last for at least 12 months?	◯ No<br>◯ Yes<br>◯ Prefer not to answer

Functional Disability Inventory: In the past two weeks, would you have had any physical trouble or difficulty doing these activities?

Walking to the bathroom	◯ No trouble<br>◯ A little trouble<br>◯ Some trouble<br>◯ A lot of trouble<br>◯ Impossible<br>◯ Prefer not to answer
Walking up stairs	◯ No trouble<br>◯ A little trouble<br>◯ Some trouble<br>◯ A lot of trouble<br>◯ Impossible<br>◯ Prefer not to answer
Doing something with a friend (For example, playing a game)	◯ No trouble<br>◯ A little trouble<br>◯ Some trouble<br>◯ A lot of trouble<br>◯ Impossible<br>◯ Prefer not to answer
Doing chores at home	◯ No trouble<br>◯ A little trouble<br>◯ Some trouble<br>◯ A lot of trouble<br>◯ Impossible<br>◯ Prefer not to answer
Eating regular meals	◯ No trouble<br>◯ A little trouble<br>◯ Some trouble<br>◯ A lot of trouble<br>◯ Impossible<br>◯ Prefer not to answer
Being up all day without a nap or rest	◯ No trouble<br>◯ A little trouble<br>◯ Some trouble<br>◯ A lot of trouble<br>◯ Impossible<br>◯ Prefer not to answer
Riding the school bus or traveling in the car	◯ No trouble<br>◯ A little trouble<br>◯ Some trouble<br>◯ A lot of trouble<br>◯ Impossible<br>◯ Prefer not to answer

Being at school all day	◯ No trouble<br>◯ A little trouble<br>◯ Some trouble<br>◯ A lot of trouble<br>◯ Impossible<br>◯ Prefer not to answer
Doing the activities in gym class (or playing sports)	◯ No trouble<br>◯ A little trouble<br>◯ Some trouble<br>◯ A lot of trouble<br>◯ Impossible<br>◯ Prefer not to answer
Reading or doing homework	◯ No trouble<br>◯ A little trouble<br>◯ Some trouble<br>◯ A lot of trouble<br>◯ Impossible<br>◯ Prefer not to answer
Watching TV	◯ No trouble<br>◯ A little trouble<br>◯ Some trouble<br>◯ A lot of trouble<br>◯ Impossible<br>◯ Prefer not to answer
Walking the length of a football field	◯ No trouble<br>◯ A little trouble<br>◯ Some trouble<br>◯ A lot of trouble<br>◯ Impossible<br>◯ Prefer not to answer
Running the length of a football field	◯ No trouble<br>◯ A little trouble<br>◯ Some trouble<br>◯ A lot of trouble<br>◯ Impossible<br>◯ Prefer not to answer
Going Shopping	◯ No trouble<br>◯ A little trouble<br>◯ Some trouble<br>◯ A lot of trouble<br>◯ Impossible<br>◯ Prefer not to answer
Getting to sleep at night and staying asleep	◯ No trouble<br>◯ A little trouble<br>◯ Some trouble<br>◯ A lot of trouble<br>◯ Impossible<br>◯ Prefer not to answer

Developmental Delay Screening/Surveillance

DURING THE PAST 12 MONTHS, did this child see a doctor, nurse, or other health care professional for sick-child care, well-child check-ups, physical exams, hospitalizations, or any other kind of medical care?	◯ No<br>◯ Yes<br>◯ Prefer not to answer
DURING THE PAST 12 MONTHS, did you, another family member or a friend have concerns about this child's learning, development, or behavior?	◯ No<br>◯ Yes<br>◯ Prefer not to answer
DURING THE PAST 12 MONTHS, did this child's doctors or other health care providers ask if you have concerns about this child's learning, development, or behavior?	◯ No<br>◯ Yes<br>◯ Prefer not to answer
DURING THE PAST 12 MONTHS, did you, another family member or a friend have concerns about this child's learning, development, or behavior that wasn't asked about by your provider?	◯ No<br>◯ Yes<br>◯ Prefer not to answer
DURING THE PAST 12 MONTHS, did a doctor or other health care provider have you or another caregiver fill out a questionnaire about observations or concerns you may have about this child's development, communication, or social behaviors?	◯ No<br>◯ Yes<br>◯ Prefer not to answer
If Yes, (2-5) Did the questionnaire ask about your concerns or observations about: Mark ALL that apply.	☐ Words and phrases this child uses and understands?<br>☐ How this child behaves and gets along with you and others?<br>☐ Prefer not to answer

Significant underlying medical conditions at the time of COVID-19 testing or diagnosis:

Diabetes type I	◯ No<br>◯ Yes<br>◯ Prefer not to answer
Diabetes type II	◯ No<br>◯ Yes<br>◯ Prefer not to answer
Obesity	◯ No<br>◯ Yes<br>◯ Prefer not to answer
Asthma	◯ No<br>◯ Yes<br>◯ Prefer not to answer
Cystic fibrosis	◯ No<br>◯ Yes<br>◯ Prefer not to answer
Cancer	◯ No<br>◯ Yes<br>◯ Prefer not to answer

Hematopoietic cell recipient/bone marrow transplant recipient	◯ No<br>◯ Yes<br>◯ Prefer not to answer
Solid organ transplant recipient	◯ No<br>◯ Yes<br>◯ Prefer not to answer
Rheumatologic conditions (e.g. rheumatoid arthritis, systemic lupus erythematosus, vasculitis)	◯ No<br>◯ Yes<br>◯ Prefer not to answer
Hypertension	◯ No<br>◯ Yes<br>◯ Prefer not to answer
Congenital heart disease	◯ No<br>◯ Yes<br>◯ Prefer not to answer
Heart failure	◯ No<br>◯ Yes<br>◯ Prefer not to answer
Cardiomyopathy	◯ No<br>◯ Yes<br>◯ Prefer not to answer
History of Kawasaki Disease (not a current diagnosis)	◯ No<br>◯ Yes<br>◯ Prefer not to answer
History of MIS-C (not a current diagnosis)	◯ No<br>◯ Yes<br>◯ Prefer not to answer
Inflammatory bowel disease	◯ No<br>◯ Yes<br>◯ Prefer not to answer
Feeding tube dependent	◯ No<br>◯ Yes<br>◯ Prefer not to answer
Sickle cell disease	◯ No<br>◯ Yes<br>◯ Prefer not to answer
Thrombotic disorders	◯ No<br>◯ Yes<br>◯ Prefer not to answer
Chronic liver disease	◯ No<br>◯ Yes<br>◯ Prefer not to answer

Seizure disorder/epilepsy	◯ No<br>◯ Yes<br>◯ Prefer not to answer
Eczema	◯ No<br>◯ Yes<br>◯ Prefer not to answer
Physical disability (including cerebral palsy)	◯ No<br>◯ Yes<br>◯ Prefer not to answer
Down syndrome	◯ No<br>◯ Yes<br>◯ Prefer not to answer
Congenital syndromes/anomalies or genetic conditions including other chromosomal syndromes	◯ No<br>◯ Yes<br>◯ Prefer not to answer
Premature or neonatal conditions	◯ No<br>◯ Yes<br>◯ Prefer not to answer
Other conditions (specify)	______________________

Premature or neonatal conditions

Fetal malnutrition	◯ No<br>◯ Yes<br>◯ Prefer not to answer
Extreme immaturity	◯ No<br>◯ Yes<br>◯ Prefer not to answer
Cerebral hemorrhage at birth	◯ No<br>◯ Yes<br>◯ Prefer not to answer
Spinal cord injury at birth	◯ No<br>◯ Yes<br>◯ Prefer not to answer
Birth asphyxia	◯ No<br>◯ Yes<br>◯ Prefer not to answer
Respiratory diseases	◯ No<br>◯ Yes<br>◯ Prefer not to answer
Hypoxic-ischemic encephalopathy	◯ No<br>◯ Yes<br>◯ Prefer not to answer

Other premature or neonatal condition	◯ No<br>◯ Yes<br>◯ Prefer not to answer

Tier2 Parent Guard

Date of Parent/Guardian Data Collection

____
(MM/DD/YYYY)

Which of the following changes in employment have occurred due to the COVID-19 pandemic?

Move to remote work, telework
- ◯ Yes (self)
- ◯ Yes (Partner only)
- ◯ Yes (Self and Partner)
- ◯ Neither (Self or Partner)
- ◯ N/A
- ◯ Prefer not to answer

Loss of hours
- ◯ Yes (self)
- ◯ Yes (Partner only)
- ◯ Yes (Self and Partner)
- ◯ Neither (Self or Partner)
- ◯ N/A
- ◯ Prefer not to answer

Decreased pay
- ◯ Yes (self)
- ◯ Yes (Partner only)
- ◯ Yes (Self and Partner)
- ◯ Neither (Self or Partner)
- ◯ N/A
- ◯ Prefer not to answer

Furloughed
- ◯ Yes (self)
- ◯ Yes (Partner only)
- ◯ Yes (Self and Partner)
- ◯ Neither (Self or Partner)
- ◯ N/A
- ◯ Prefer not to answer

Loss of job
- ◯ Yes (self)
- ◯ Yes (Partner only)
- ◯ Yes (Self and Partner)
- ◯ Neither (Self or Partner)
- ◯ N/A
- ◯ Prefer not to answer

Decreased job security
- ◯ Yes (self)
- ◯ Yes (Partner only)
- ◯ Yes (Self and Partner)
- ◯ Neither (Self or Partner)
- ◯ N/A
- ◯ Prefer not to answer

Disruptions due to childcare challenges
- ◯ Yes (self)
- ◯ Yes (Partner only)
- ◯ Yes (Self and Partner)
- ◯ Neither (Self or Partner)
- ◯ N/A
- ◯ Prefer not to answer

Increased hours	◯ Yes (self)<br>◯ Yes (Partner only)<br>◯ Yes (Self and Partner)<br>◯ Neither (Self or Partner)<br>◯ N/A<br>◯ Prefer not to answer
Another change	◯ Yes (self)<br>◯ Yes (Partner only)<br>◯ Yes (Self and Partner)<br>◯ Neither (Self or Partner)<br>◯ N/A<br>◯ Prefer not to answer
Specify	__________________________________

How difficult is/was it to meet each of the following needs for you and/or your family during the COVID-19 pandemic (since March 2020)?

Have enough money for food	◯ Not difficult<br>◯ Somewhat difficult<br>◯ Very difficult<br>◯ Prefer not to answer
Have enough money to pay for electricity, heating, or water	◯ Not difficult<br>◯ Somewhat difficult<br>◯ Very difficult<br>◯ Prefer not to answer
Have enough money to pay for housing	◯ Not difficult<br>◯ Somewhat difficult<br>◯ Very difficult<br>◯ Prefer not to answer
Get help from community organizations that I trust	◯ Not difficult<br>◯ Somewhat difficult<br>◯ Very difficult<br>◯ Prefer not to answer
Get help from family members and friends	◯ Not difficult<br>◯ Somewhat difficult<br>◯ Very difficult<br>◯ Prefer not to answer
See a healthcare provider if you or your family needs it	◯ Not difficult<br>◯ Somewhat difficult<br>◯ Very difficult<br>◯ Prefer not to answer
Get routine/essential medications	◯ Not difficult<br>◯ Somewhat difficult<br>◯ Very difficult<br>◯ Prefer not to answer

Question	Response
Get transportation when you need it	◯ Not difficult<br>◯ Somewhat difficult<br>◯ Very difficult<br>◯ Prefer not to answer
Use the internet for things like work, school, medical visits, socializing	◯ Not difficult<br>◯ Somewhat difficult<br>◯ Very difficult<br>◯ Prefer not to answer
Thinking about the future, over the next 3 months how challenging will it be to make ends meet?	◯ A lot more challenging than usual<br>◯ A little more challenging than usual<br>◯ No more challenging than usual<br>◯ Less challenging than usual<br>◯ Don't know<br>◯ Prefer not to answer
In the last 12 months, if you didn't have enough to eat or what you wanted to eat, why was that? Choose all that apply.	☐ Couldn't get out to buy food (for example, didn't have transportation, or had mobility or health problems that prevented you from getting out)<br>☐ Didn't want to go out to buy food<br>☐ Afraid to go out because of the chance of contracting COVID-19<br>☐ Couldn't get groceries or meals delivered to me<br>☐ The stores didn't have the food I wanted<br>☐ Other<br>☐ I always had enough to eat and what I wanted to eat<br>☐ Prefer not to answer
Specify	__________________________________
How [have/were] you and your child(ren) (been) getting along during the COVID-19 outbreak (since March 2020)?	◯ Very well - no problems or tension<br>◯ Well - occasional tension, some tension, but manageable<br>◯ Okay - some tension and sometimes things get out of hand (a few heated arguments)<br>◯ Not very well - tense, lots of arguing, unsettled feeling, definite problems<br>◯ Terribly<br>◯ Prefer not to answer
Is this a change from how you were getting along during the COVID-19 outbreak (since March 2020)?	◯ Yes<br>◯ No<br>◯ Prefer not to answer

For each of these statements, please state whether you strongly agree, agree, neither agree nor disagree, disagree or strongly disagree.

Question	Response
This is a close-knit neighborhood.	◯ Strongly Agree<br>◯ Agree<br>◯ Neither Agree nor Disagree<br>◯ Disagree<br>◯ Strongly Disagree<br>◯ Prefer not to answer

People around here are willing to help their neighbors.	◯ Strongly Agree<br>◯ Agree<br>◯ Neither Agree nor Disagree<br>◯ Disagree<br>◯ Strongly Disagree<br>◯ Prefer not to answer
People in this neighborhood generally don't get along with each other.	◯ Strongly Agree<br>◯ Agree<br>◯ Neither Agree nor Disagree<br>◯ Disagree<br>◯ Strongly Disagree<br>◯ Prefer not to answer
People in this neighborhood do not share the same values.	◯ Strongly Agree<br>◯ Agree<br>◯ Neither Agree nor Disagree<br>◯ Disagree<br>◯ Strongly Disagree<br>◯ Prefer not to answer
People in this neighborhood can be trusted.	◯ Strongly Agree<br>◯ Agree<br>◯ Neither Agree nor Disagree<br>◯ Disagree<br>◯ Strongly Disagree<br>◯ Prefer not to answer

For each of the following, state whether it is very likely, likely, neither likely nor unlikely, unlikely, or very unlikely that people in your neighborhood would act in the following manner.

If a group of neighborhood children were skipping school and hanging out on a street corner, how likely is it that your neighbors would do something about it?	◯ Very likely<br>◯ Likely<br>◯ Neither likely not unlikely<br>◯ Unlikely<br>◯ Very unlikely<br>◯ Prefer not to answer
If some children were spray-painting graffiti on a local building, how likely is it that your neighbors would do something about it?	◯ Very likely<br>◯ Likely<br>◯ Neither likely not unlikely<br>◯ Unlikely<br>◯ Very unlikely<br>◯ Prefer not to answer
If a child was showing disrespect to an adult, how likely is it that people in your neighborhoodwould scold that child?	◯ Very likely<br>◯ Likely<br>◯ Neither likely not unlikely<br>◯ Unlikely<br>◯ Very unlikely<br>◯ Prefer not to answer
If there was a fight in front of your house and someone was being beaten or threatened, howlikely is it that your neighbors would break it up?	◯ Very likely<br>◯ Likely<br>◯ Neither likely not unlikely<br>◯ Unlikely<br>◯ Very unlikely<br>◯ Prefer not to answer

Suppose that because of budget cuts the fire station closest to your home was going to be closed by the city. How likely is it that neighborhood residents would organize totry to do something to keep the fire station open?	◯ Very likely<br>◯ Likely<br>◯ Neither likely not unlikely<br>◯ Unlikely<br>◯ Very unlikely<br>◯ Prefer not to answer
If it were necessary, could a member of your household isolate themselves from the rest of your household due to suspected COVID-19 infection for as long as needed? (To effectively isolate during a COVID-19 infection, the infected family member would need to stay in a specific "sickroom" away from other people or animals and, if possible, use a separate bathroom.)	◯ Yes<br>◯ No<br>◯ Don't know<br>◯ Prefer not to answer
I believe that COVID-19 is a serious disease.	◯ Yes<br>◯ No<br>◯ Don't know<br>◯ Prefer not to answer
To the best of your knowledge, which of the following can protect you and your family from COVID-19? (Mark all that apply)	☐ Standing 6 feet from another person<br>☐ Wearing a face mask<br>☐ Working from home<br>☐ Distance learning (or taking school classes over the computer or remotely)<br>☐ Vaccination for COVID-19<br>☐ Prefer not to answer
Which of the following applies to your plans about the COVID vaccine for your child(ren)?	◯ My child(ren) is/are already vaccinated<br>◯ I plan on getting the COVID vaccine for my child(ren) as soon as it is available<br>◯ I plan on getting the COVID vaccine for my child(ren) eventually<br>◯ I do not plan on getting the COVID vaccine for my child(ren)<br>◯ I am unsure<br>◯ Prefer not to answer
Which of the following applies to your plans about the COVID vaccine for your child(ren) who are not yet eligible to receive the COVID vaccine?	◯ I plan on getting the COVID vaccine for my child(ren) eventually<br>◯ I plan on getting the COVID vaccine for my child(ren) as soon as they are eligible<br>◯ I do not plan on getting the COVID vaccine for my child(ren)<br>◯ I am unsure<br>◯ Prefer not to answer

If you do not plan on getting the COVID vaccine for your child(ren), why not? (Mark all that apply)	☐ Not available<br>☐ Doctor/healthcare provider did not recommend<br>☐ My friends and family did not recommend<br>☐ I have read information that suggests it is unsafe<br>☐ The vaccine was not well tested in ethnically diverse people<br>☐ The vaccine was not well tested among children<br>☐ I cannot afford the vaccine<br>☐ I do not have time to take my child to be vaccinated<br>☐ My child is at low risk and does not need it<br>☐ It is riskier to go and get it than to stay at home<br>☐ Worried about side effects<br>☐ The vaccine's technology hasn't been tested enough<br>☐ The vaccine was approved too fast<br>☐ No long-term safety data available<br>☐ Concerned about vaccine storage<br>☐ My child already had COVID-19<br>☐ Other (please specify)<br>☐ Prefer not to answer
Specify	______________________________
In general, how is your physical health?	◯ Excellent<br>◯ Very good<br>◯ Good<br>◯ Fair<br>◯ Poor<br>◯ Prefer not to answer
In general, how is your mental or emotional health?	◯ Excellent<br>◯ Very good<br>◯ Good<br>◯ Fair<br>◯ Poor<br>◯ Prefer not to answer

Tier2 Long Covid

Date of Long COVID Data Collection

____
(MM/DD/YYYY)

COVID Care

The following questions will ask you about the care you received for your COVID-19 infection(s).

What was the highest level of care you had for any of your COVID-19 infection(s)?

- ☐ Hospital ICU
- ☐ Hospital in-patient
- ☐ Emergency Department
- ☐ Urgent Care/ Walk-in clinic
- ☐ Primary doctor
- ☐ Community health center
- ☐ Stayed home (isolated, rested, drank water, etc.)
- ☐ I did not seek treatment (including self-treatment or isolation)
- ☐ Prefer not to answer

What month did your highest level of care start?

- ◯ January
- ◯ February
- ◯ March
- ◯ April
- ◯ May
- ◯ June
- ◯ July
- ◯ August
- ◯ September
- ◯ October
- ◯ November
- ◯ December
- ◯ Prefer not to answer

What year did your highest level of care start?

- ◯ 2019
- ◯ 2020
- ◯ 2021
- ◯ 2022
- ◯ 2023
- ◯ Prefer not to answer

Long COVID Symptoms

Instructions: Next we will ask you about symptoms you may have had since your first COVID-19 infection.

Since your first COVID-19 infection, have you had new or worsening problems breathing?

- ◯ Yes
- ◯ No
- ◯ Don't Know
- ◯ Prefer not to answer

Since your first COVID-19 infection, have you had new or worsening heart problems and/or heart attack?

- ◯ Yes
- ◯ No
- ◯ Don't Know
- ◯ Prefer not to answer

Since your first COVID-19 infection, have you had a stroke?	◯ Yes<br>◯ No<br>◯ Don't Know<br>◯ Prefer not to answer
Since your first COVID-19 infection, have you had new or worsening difficulty managing your blood sugar?	◯ Yes<br>◯ No<br>◯ Don't Know<br>◯ Prefer not to answer
Since your first COVID-19 infection, have you had new or worsening brain fog (confusion, lack of focus, being forgetful, etc.)?	◯ Yes<br>◯ No<br>◯ Don't Know<br>◯ Prefer not to answer
Since your first COVID-19 infection, have you had new or worsening fatigue (tire easily, decreased energy, etc.)?	◯ Yes<br>◯ No<br>◯ Don't Know<br>◯ Prefer not to answer
Since your first COVID-19 infection, have you had new or worsening problems sleeping?	◯ Yes<br>◯ No<br>◯ Don't Know<br>◯ Prefer not to answer
Since your first COVID-19 infection, have you had new, worsening, or more frequent headaches?	◯ Yes<br>◯ No<br>◯ Don't Know<br>◯ Prefer not to answer
Since your first COVID -19 infection, have you had any changes in your mood?	◯ Yes<br>◯ No<br>◯ Don't Know<br>◯ Prefer not to answer
Since your first COVID-19 infection, have you experienced changes to your menstrual cycle?	◯ Yes<br>◯ No<br>◯ Don't Know<br>◯ Prefer not to answer
Since your first COVID-19 infection, have you developed any other physical or mental health issues?	◯ Yes<br>◯ No<br>◯ Don't Know<br>◯ Prefer not to answer
Think about the symptoms above. How many weeks did you have these symptoms?	________________________
Have any of the symptoms gone away?	◯ Yes<br>◯ No<br>◯ Prefer not to answer
Since your first COVID-19 infection, have you started any new treatments related to any of the symptoms listed above?	◯ Yes<br>◯ No<br>◯ Don't Know<br>◯ Prefer not to answer

Question	Response
When your symptoms were the worst, which of the following applied:	◯ I was only short of breath with strenuous exercise<br>◯ I got short of breath when hurrying on level ground or walking up a slight hill<br>◯ On level ground, I walked slower than people of my age because of shortness of breath, or I had to stop for breath when walking at my own pace<br>◯ On level ground, I stopped for breath after walking about 100 yeards or after a few minutes<br>◯ I was too short of breath to leave the house or I was short of breath when dressing/undressing
How many weeks were you unable to go about your normal day?	__________________________

Long COVID Knowledge

The following questions will ask you about your experience with Long COVID.

Question	Response
Do you know what Long COVID is?	◯ Yes<br>◯ No<br>◯ Prefer not to answer

Long COVID is an illness that people may get after COVID-19. Common signs include fatigue, breathing problems, brain fog, stroke, heart attack, and poor control of blood sugar.

Long COVID is sometimes called long-haul COVID, post COVID-19, long-term effects of COVID, or chronic COVID.

Question	Response
Who do you trust to give you information about Long COVID?<br>(Check all that apply)	☐ Your doctor or healthcare provider<br>☐ Your faith leader<br>☐ Your close friends and members of your family<br>☐ People you go to work or class with or other people you know<br>☐ News on the radio, TV, online, or in newspapers<br>☐ Your contacts on social media<br>☐ The U.S. government<br>☐ Prefer not to answer
Has a doctor diagnosed you with Long COVID?	◯ Yes<br>◯ No<br>◯ Don't Know<br>◯ Prefer not to answer

Everyday Life Experience

Instructions: Next we will ask you about your everyday life.

Question	Response
How much has having Long COVID affected your everyday life?	◯ Not at all<br>◯ Slightly<br>◯ Moderately<br>◯ Very<br>◯ Extremely<br>◯ Prefer not to answer

Which of the following areas of your life have your Long COVID symptoms affected?<br>(Check one or more answers)	☐ None<br>☐ Family<br>☐ Work or school<br>☐ Spending time with friends<br>☐ Hobbies/activities<br>☐ Doing household duties or chores<br>☐ Exercising/being active<br>☐ Prefer not to answer
How much has having Long COVID (not COVID-19 itself) affected your family's everyday life?	◯ Not at all<br>◯ Slightly<br>◯ Moderately<br>◯ Very<br>◯ Extremely<br>◯ Prefer not to answer
How much do you think Long COVID (not COVID-19 itself) is affecting your friends and communities everyday lives?	◯ Not at all<br>◯ Slightly<br>◯ Moderately<br>◯ Very<br>◯ Extremely<br>◯ Don't know<br>◯ Prefer not to answer
Do you know of a place that specializes in Long COVID diagnosis and care near where you live?	◯ Yes<br>◯ No<br>◯ Prefer not to answer

Note to implementer: Use this space to provide list of Long COVID clinics or information/resources for Long COVID available in your area.
