# Supplementary material for: A community-engaged approach to developing common data elements: a case study from the RADx-UP Long COVID common data elements Task Force
Source: JAMIA Open. 2025 Jun 4;8(3):ooaf046. doi: 10.1093/jamiaopen/ooaf046 (PMC12136053; doi:10.1093/jamiaopen/ooaf046)
Supplement: ooaf046_Supplementary_Data [file ooaf046_supplementary_data.zip › Supplementary Survey 1_StarterPack(RECOVER)CDEs_Feedback_Survey.pdf]

Introduction

Thank you for your participation in this survey. The purpose of this survey is to get feedback on the Long COVID CDE Starter Pack. You are being asked to review the Long COVID CDE Starter Pack and provide feedback on the content and the overall wording of the questions. We are interested in knowing if the Starter Pack is easy to understand, comprehensive, and inclusive to a wide range of communities. Results of this survey will be used to inform the Long COVID CDE Task Force and help them prioritize Common Data Elements (CDEs) that need to be included, should be excluded, and need to be changed.

This survey is voluntary, and you are free to skip any questions you do not want to answer or stop completing the survey at any point. The survey should take approximately 30 - 45 minutes, and you will be compensated for your time.

You can move forward and backward within the survey and edit any of your responses until you submit the survey. You are welcome to go back and review the Long COVID CDE document as you complete the survey.

If you have any questions regarding the Long COVID CDE Starter Pack or this survey, please contact Allyn Damman (allyn.damman@duke.edu).

User Information

These questions help us to better understand who is completing the survey. The information will also help us ensure that we obtain feedback from a diverse group of users.

First Name:

Last Name:

Email:

General PASC Symptoms

The next questions focus on the **General Post-Acute Sequelae of COVID-19 (PASC)** Symptoms section of the Long COVID CDE Starter Pack. As a reminder, you can use the CDE Starter Pack to refer to the items that are being asked about.

Do you want to skip this section?

- Yes
- No

You answered YES -- please indicate why you decided to skip this set of questions

- This section is not relevant to me.
- This section is fine – I have no comments or concerns.
- Other: Please provide explanation

The PASC questions were easy to understand.

- Strongly disagree
- Disagree
- Neither agree nor disagree
- Agree
- Strongly agree

Please provide additional feedback on how the wording of the questions in this section could be easier to understand.

The items are relevant to Long COVID general symptoms.

- Strongly disagree
- Disagree
- Neither agree nor disagree
- Agree
- Strongly agree

Please provide additional feedback on how the items in this section could be more relevant to general symptoms of Long COVID.

All items in this subsection address all aspects of General PASC Symptoms of Long COVID.

- Strongly disagree
- Disagree
- Neither agree nor disagree
- Agree
- Strongly agree

Please explain any additional aspects of General PASC Symptoms of Long COVID not addressed in this section.

Are there any additional items you suggest we add that are missing from this section? If yes, please list them in the space provided.

Pain

The next questions focus on the **Pain** symptoms section of the Long COVID CDE Starter Pack. As a reminder, you can use the CDE Starter Pack to refer to the items that are being asked about

Do you want to skip this section?

- Yes
- No

You answered YES -- please indicate why you decided to skip this set of questions

- This section is not relevant to me.
- This section is fine – I have no comments or concerns.

- Other: Please provide explanation

The pain questions were easy to understand.

- Strongly disagree
- Disagree
- Neither agree nor disagree
- Agree
- Strongly agree

Please provide additional feedback on how the wording of the questions in this section could be easier to understand.

The items are relevant to Long COVID pain symptoms.

- Strongly disagree
- Disagree
- Neither agree nor disagree
- Agree
- Strongly agree

Please provide additional feedback on how the items in this section could be more relevant to pain symptoms of Long COVID.

All items in this subsection address all aspects of pain symptoms of Long COVID.

- Strongly disagree
- Disagree
- Neither agree nor disagree
- Agree
- Strongly agree

Please explain any additional aspects of pain symptoms of Long COVID not addressed in this section.

Are there any additional items you suggest we add that are missing from this section? If yes, please list them in the space provided.

Headache

The next questions focus on the **Headache** symptoms section of the Long COVID CDE Starter Pack. As a reminder, you can use the CDE Starter Pack to refer to the items that are being asked about.

Do you want to skip this section?

- Yes
- No

You answered YES -- please indicate why you decided to skip this set of questions

- This section is not relevant to me.
- This section is fine – I have no comments or concerns.
- Other: Please provide explanation

The headache questions were easy to understand.

- Strongly disagree
- Disagree
- Neither agree nor disagree
- Agree
- Strongly agree

Please provide additional feedback on how the wording of the questions in this section could be easier to understand.

The items are relevant to Long COVID headache symptoms.

- Strongly disagree
- Disagree
- Neither agree nor disagree
- Agree
- Strongly agree

Please provide additional feedback on how the items in this section could be more relevant to headache symptoms of Long COVID.

All items in this subsection address all aspects of headache symptoms of Long COVID.

- Strongly disagree
- Disagree
- Neither agree nor disagree
- Agree
- Strongly agree

Please explain any additional aspects of headache symptoms of Long COVID not addressed in this section.

Are there any additional items you suggest we add that are missing from this section? If yes, please list them in the space provided.

Chest Pain

The next questions focus on the **Chest Pain** symptoms section of the Long COVID CDE Starter Pack. As a reminder, you can use the CDE Starter Pack to refer to the items that are being asked about.

Do you want to skip this section?

- Yes
- No

You answered YES -- please indicate why you decided to skip this set of questions

- This section is not relevant to me.
- This section is fine – I have no comments or concerns.
- Other: Please provide explanation

The chest pain questions were easy to understand.

- Strongly disagree
- Disagree
- Neither agree nor disagree
- Agree
- Strongly agree

Please provide additional feedback on how the wording of the questions in this section could be easier to understand.

The items are relevant to Long COVID chest pain symptoms.

- Strongly disagree
- Disagree
- Neither agree nor disagree
- Agree
- Strongly agree

Please provide additional feedback on how the items in this section could be more relevant to chest pain symptoms of Long COVID.

All items in this subsection address all aspects of chest pain symptoms of Long COVID.

- Strongly disagree
- Disagree
- Neither agree nor disagree
- Agree
- Strongly agree

Please explain any additional aspects of chest pain symptoms of Long COVID not addressed in this section.

Are there any additional items you suggest we add that are missing from this section? If yes, please list them in the space provided.

Shortness of Breath

The next questions focus on the **Shortness of Breath** symptoms section of the Long COVID CDE Starter Pack. As a reminder, you can use the CDE Starter Pack to refer to the items that are being asked about.

Do you want to skip this section?

- Yes

- No

You answered YES -- please indicate why you decided to skip this set of questions

- This section is not relevant to me.
- This section is fine – I have no comments or concerns.
- Other: Please provide explanation

The shortness of breath questions were easy to understand.

- Strongly disagree
- Disagree
- Neither agree nor disagree
- Agree
- Strongly agree

Please provide additional feedback on how the wording of the questions in this section could be easier to understand.

The items are relevant to Long COVID shortness of breath symptoms.

- Strongly disagree
- Disagree
- Neither agree nor disagree
- Agree

- Strongly agree

Please provide additional feedback on how the items in this section could be more relevant to shortness of breath symptoms of Long COVID.

All items in this subsection address all aspects of shortness of breath symptoms of Long COVID.

- Strongly disagree
- Disagree
- Neither agree nor disagree
- Agree
- Strongly agree

Please explain any additional aspects of shortness of breath symptoms of Long COVID not addressed in this section.

Are there any additional items you suggest we add that are missing from this section? If yes, please list them in the space provided.

Nerve Problems

The next questions focus on the **Nerve Problems** symptoms section of the Long COVID CDE Starter Pack. As a reminder, you can use the CDE Starter Pack to refer to the items that are being asked about.

Do you want to skip this section?

- Yes
- No

You answered YES -- please indicate why you decided to skip this set of questions

- This section is not relevant to me.
- This section is fine – I have no comments or concerns.
- Other: Please provide explanation

The nerve problems questions were easy to understand.

- Strongly disagree
- Disagree
- Neither agree nor disagree
- Agree
- Strongly agree

Please provide additional feedback on how the wording of the questions in this section could be easier to understand.

The items are relevant to Long COVID nerve problems symptoms.

- Strongly disagree
- Disagree
- Neither agree nor disagree
- Agree
- Strongly agree

Please provide additional feedback on how the items in this section could be more relevant to nerve problems symptoms of Long COVID.

All items in this subsection address all aspects of nerve problems symptoms of Long COVID.

- Strongly disagree
- Disagree
- Neither agree nor disagree
- Agree
- Strongly agree

Please explain any additional aspects of nerve problems symptoms of Long COVID not addressed in this section.

Are there any additional items you suggest we add that are missing from this section? If yes, please list them in the space provided.

Neuropathy

The next questions focus on the **Neuropathy** symptoms section of the Long COVID CDE Starter Pack. As a reminder, you can use the CDE Starter Pack to refer to the items that are being asked about.

Do you want to skip this section?

- Yes
- No

You answered YES -- please indicate why you decided to skip this set of questions

- This section is not relevant to me.
- This section is fine – I have no comments or concerns.

- Other: Please provide explanation

The neuropathy questions were easy to understand.

- Strongly disagree
- Disagree
- Neither agree nor disagree
- Agree
- Strongly agree

Please provide additional feedback on how the wording of the questions in this section could be easier to understand.

The items are relevant to Long COVID neuropathy symptoms.

- Strongly disagree
- Disagree
- Neither agree nor disagree
- Agree
- Strongly agree

Please provide additional feedback on how the items in this section could be more relevant to neuropathy symptoms of Long COVID.

All items in this subsection address all aspects of neuropathy symptoms of Long COVID.

- Strongly disagree
- Disagree
- Neither agree nor disagree
- Agree
- Strongly agree

Please explain any additional aspects of neuropathy symptoms of Long COVID not addressed in this section.

Are there any additional items you suggest we add that are missing from this section? If yes, please list them in the space provided.

Weakness, Numbness and Tingling in Arms and Legs

The next questions focus on the **Weakness, Numbness and Tingling in Arms and Legs** Symptoms section of the Long COVID CDE Starter Pack. As a reminder, you can use the CDE Starter Pack to refer to the items that are being asked about.

Do you want to skip this section?

- Yes
- No

You answered YES -- please indicate why you decided to skip this set of questions

- This section is not relevant to me.
- This section is fine – I have no comments or concerns.
- Other: Please provide explanation

The Weakness, Numbness and Tingling in Arms and Legs wording of the questions is easy to understand.

- Strongly disagree
- Disagree
- Neither agree nor disagree
- Agree
- Strongly agree

Please provide additional feedback on how the wording of the questions in this section could be easier to understand.

The items are relevant to Long COVID weakness, numbness and tingling in arms and legs symptoms.

- Strongly disagree
- Disagree
- Neither agree nor disagree
- Agree
- Strongly agree

Please provide additional feedback on how the items in this section could be more relevant to weakness, numbness and tingling in arms and legs symptoms of Long COVID.

All items in this subsection address all aspects of weakness, numbness and tingling in arms and legs symptoms of Long COVID.

- Strongly disagree
- Disagree
- Neither agree nor disagree
- Agree
- Strongly agree

Please explain any additional aspects of weakness, numbness and tingling in arms and legs symptoms of Long COVID not addressed in this section.

Are there any additional items you suggest we add that are missing from this section? If yes, please list them in the space provided.

Cognitive Function

The next questions focus on the **Cognitive Function** Symptoms section of the Long COVID CDE Starter Pack. As a reminder, you can use the CDE Starter Pack to refer to the items that are being asked about.

Do you want to skip this section?

- Yes
- No

You answered YES -- please indicate why you decided to skip this set of questions

- This section is not relevant to me.
- This section is fine – I have no comments or concerns.
- Other: Please provide explanation

The cognitive function questions were easy to understand.

- Strongly disagree
- Disagree
- Neither agree nor disagree
- Agree
- Strongly agree

Please provide additional feedback on how the wording of the questions in this section could be easier to understand.

The items are relevant to Long COVID cognitive function symptoms.

- Strongly disagree
- Disagree
- Neither agree nor disagree
- Agree
- Strongly agree

Please provide additional feedback on how the items in this section could be more relevant to cognitive function symptoms of Long COVID.

All items in this subsection address all aspects of cognitive function symptoms of Long COVID.

- Strongly disagree
- Disagree
- Neither agree nor disagree
- Agree
- Strongly agree

Please explain any additional aspects of cognitive function symptoms of Long COVID not addressed in this section.

Are there any additional items you suggest we add that are missing from this section? If yes, please list them in the space provided.

Sleep Symptoms

The next questions focus on the **Sleep** Symptoms section of the Long COVID CDE Starter Pack. As a reminder, you can use the CDE Starter Pack to refer to the items that are being asked about.

Do you want to skip this section?

- Yes
- No

You answered YES -- please indicate why you decided to skip this set of questions

- This section is not relevant to me.
- This section is fine – I have no comments or concerns.
- Other: Please provide explanation

The sleep questions were easy to understand.

- Strongly disagree
- Disagree
- Neither agree nor disagree
- Agree
- Strongly agree

Please provide additional feedback on how the wording of the questions in this section could be easier to understand.

The items are relevant to Long COVID sleep symptoms.

- Strongly disagree
- Disagree
- Neither agree nor disagree

- Agree
- Strongly agree

Please provide additional feedback on how the items in this section could be more relevant to sleep symptoms of Long COVID.

All items in this subsection address all aspects of sleep symptoms of Long COVID.

- Strongly disagree
- Disagree
- Neither agree nor disagree
- Agree
- Strongly agree

Please explain any additional aspects of sleep symptoms of Long COVID not addressed in this section.

Are there any additional items you suggest we add that are missing from this section? If yes, please list them in the space provided.

Hypersomnia (Daytime Sleepiness) and Insomnia Symptoms

The next questions focus on the **Hypersomnia (Daytime Sleepiness) and Insomnia** Symptoms section of the Long COVID CDE Starter Pack. As a reminder, you can use the CDE Starter Pack to refer to the items that are being asked about.

Do you want to skip this section?

- Yes
- No

You answered YES -- please indicate why you decided to skip this set of questions

- This section is not relevant to me.
- This section is fine – I have no comments or concerns.
- Other: Please provide explanation

The Hypersomnia (Daytime Sleepiness) and Insomnia questions were easy to understand.

- Strongly disagree
- Disagree
- Neither agree nor disagree
- Agree
- Strongly agree

Please provide additional feedback on how the wording of the questions in this section could be easier to understand.

The items are relevant to Long COVID hypersomnia and insomnia symptoms.

- Strongly disagree
- Disagree
- Neither agree nor disagree
- Agree
- Strongly agree

Please provide additional feedback on how the items in this section could be more relevant to hypersomnia and insomnia symptoms of Long COVID.

All items in this subsection address all aspects of hypersomnia and insomnia symptoms of Long COVID.

- Strongly disagree
- Disagree
- Neither agree nor disagree
- Agree
- Strongly agree

Please explain any additional aspects of hypersomnia and insomnia symptoms of Long COVID not addressed in this section.

Are there any additional items you suggest we add that are missing from this section? If yes, please list them in the space provided.

Vision Problems

The next questions focus on the **Vision Problems** Symptoms section of the Long COVID CDE Starter Pack. As a reminder, you can use the CDE Starter Pack to refer to the items that are being asked about.

Do you want to skip this section?

- Yes
- No

You answered YES -- please indicate why you decided to skip this set of questions

- This section is not relevant to me.
- This section is fine – I have no comments or concerns.

- Other: Please provide explanation

The vision problems questions were easy to understand.

- Strongly disagree
- Disagree
- Neither agree nor disagree
- Agree
- Strongly agree

Please provide additional feedback on how the wording of the questions in this section could be easier to understand.

The items are relevant to Long COVID vision problems.

- Strongly disagree
- Disagree
- Neither agree nor disagree
- Agree
- Strongly agree

Please provide additional feedback on how the items in this section could be more relevant to vision problem symptoms of Long COVID.

All items in this subsection address all aspects of vision problem symptoms of Long COVID.

- Strongly disagree
- Disagree
- Neither agree nor disagree
- Agree
- Strongly agree

Please explain any additional aspects of vision problem symptoms of Long COVID not addressed in this section.

Are there any additional items you suggest we add that are missing from this section? If yes, please list them in the space provided.

Feeling Dizzy, Faint or Goofy

The next questions focus on the **Feeling Dizzy, Faint or Goofy** Symptoms section of the Long COVID CDE Starter Pack. As a reminder, you can use the CDE Starter Pack to refer to the items that are being asked about.

Do you want to skip this section?

- Yes
- No

You answered YES -- please indicate why you decided to skip this set of questions

- This section is not relevant to me.
- This section is fine – I have no comments or concerns.
- Other: Please provide explanation

The Feeling Dizzy, Faint or Goofy questions were easy to understand.

- Strongly disagree
- Disagree
- Neither agree nor disagree
- Agree
- Strongly agree

Please provide additional feedback on how the wording of the questions in this section could be easier to understand.

The items are relevant to Long COVID feeling dizzy, faint, or goofy symptoms.

- Strongly disagree
- Disagree
- Neither agree nor disagree
- Agree
- Strongly agree

Please provide additional feedback on how the items in this section could be more relevant to feeling dizzy, faint, or goofy symptoms of Long COVID.

All items in this subsection address all aspects of feeling dizzy, faint, or goofy symptoms of Long COVID.

- Strongly disagree
- Disagree
- Neither agree nor disagree
- Agree
- Strongly agree

Please explain any additional aspects of feeling dizzy, faint, or goofy symptoms of Long COVID not addressed in this section.

Are there any additional items you suggest we add that are missing from this section? If yes, please list them in the space provided.

Skin Problems

The next questions focus on the **Skin Problems** Symptoms section of the Long COVID CDE Starter Pack. As a reminder, you can use the CDE Starter Pack to refer to the items that are being asked about.

Do you want to skip this section?

- Yes
- No

You answered YES -- please indicate why you decided to skip this set of questions

- This section is not relevant to me.
- This section is fine – I have no comments or concerns.
- Other: Please provide explanation

The skin problems questions were easy to understand.

- Strongly disagree
- Disagree
- Neither agree nor disagree
- Agree
- Strongly agree

Please provide additional feedback on how the wording of the questions in this section could be easier to understand.

The items are relevant to Long COVID skin problem symptoms.

- Strongly disagree
- Disagree
- Neither agree nor disagree
- Agree
- Strongly agree

Please provide additional feedback on how the items in this section could be more relevant to skin problem symptoms of Long COVID.

All items in this subsection address all aspects of skin problem symptoms of Long COVID.

- Strongly disagree
- Disagree
- Neither agree nor disagree
- Agree
- Strongly agree

Please explain any additional aspects of skin problem symptoms of Long COVID not addressed in this section.

Are there any additional items you suggest we add that are missing from this section? If yes, please list them in the space provided.

Dry Mouth

The next questions focus on the **Dry Mouth** Symptoms section of the Long COVID CDE Starter Pack. As a reminder, you can use the CDE Starter Pack to refer to the items that are being asked about.

Do you want to skip this section?

- Yes

- No

You answered YES -- please indicate why you decided to skip this set of questions

- This section is not relevant to me.
- This section is fine – I have no comments or concerns.
- Other: Please provide explanation

The dry mouth questions were easy to understand.

- Strongly disagree
- Disagree
- Neither agree nor disagree
- Agree
- Strongly agree

Please provide additional feedback on how the wording of the questions in this section could be easier to understand.

The items are relevant to Long COVID dry mouth symptoms.

- Strongly disagree
- Disagree
- Neither agree nor disagree
- Agree

- Strongly agree

Please provide additional feedback on how the items in this section could be more relevant to dry mouth symptoms of Long COVID.

All items in this subsection address all aspects of dry mouth symptoms of Long COVID.

- Strongly disagree
- Disagree
- Neither agree nor disagree
- Agree
- Strongly agree

Please explain any additional aspects of dry mouth symptoms of Long COVID not addressed in this section.

Are there any additional items you suggest we add that are missing from this section? If yes, please list them in the space provided.

Stomach Problems

The next questions focus on the **Stomach Problem** Symptoms section of the Long COVID CDE Starter Pack. As a reminder, you can use the CDE Starter Pack to refer to the items that are being asked about.

Do you want to skip this section?

- Yes
- No

You answered YES -- please indicate why you decided to skip this set of questions

- This section is not relevant to me.
- This section is fine – I have no comments or concerns.
- Other: Please provide explanation

The stomach problems questions were easy to understand.

- Strongly disagree
- Disagree
- Neither agree nor disagree
- Agree
- Strongly agree

Please provide additional feedback on how the wording of the questions in this section could be easier to understand.

The items are relevant to Long COVID stomach problem symptoms.

- Strongly disagree
- Disagree
- Neither agree nor disagree
- Agree
- Strongly agree

Please provide additional feedback on how the items in this section could be more relevant to stomach problem symptoms of Long COVID.

All items in this subsection address all aspects of stomach problem symptoms of Long COVID.

- Strongly disagree
- Disagree
- Neither agree nor disagree
- Agree
- Strongly agree

Please explain any additional aspects of stomach problem symptoms of Long COVID not addressed in this section.

Are there any additional items you suggest we add that are missing from this section? If yes, please list them in the space provided.

Bladder Problems

The next questions focus on the **Bladder Problems** Symptoms section of the Long COVID CDE Starter Pack. As a reminder, you can use the CDE Starter Pack to refer to the items that are being asked about.

Do you want to skip this section?

- Yes
- No

You answered YES -- please indicate why you decided to skip this set of questions

- This section is not relevant to me.
- This section is fine – I have no comments or concerns.

- Other: Please provide explanation

The bladder problems questions were easy to understand.

- Strongly disagree
- Disagree
- Neither agree nor disagree
- Agree
- Strongly agree

Please provide additional feedback on how the wording of the questions in this section could be easier to understand.

The items are relevant to Long COVID bladder problem symptoms.

- Strongly disagree
- Disagree
- Neither agree nor disagree
- Agree
- Strongly agree

Please provide additional feedback on how the items in this section could be more relevant to bladder problem symptoms of Long COVID.

All items in this subsection address all aspects of bladder problem symptoms of Long COVID.

- Strongly disagree
- Disagree
- Neither agree nor disagree
- Agree
- Strongly agree

Please explain any additional aspects of bladder problem symptoms of Long COVID not addressed in this section.

Are there any additional items you suggest we add that are missing from this section? If yes, please list them in the space provided.

PTSD Symptoms

The next questions focus on the **Post-Traumatic Stress Disorder (PTSD)** Symptoms section of the Long COVID CDE Starter Pack. As a reminder, you can use the CDE Starter Pack to refer to the items that are being asked about.

Do you want to skip this section?

- Yes
- No

You answered YES -- please indicate why you decided to skip this set of questions

- This section is not relevant to me.
- This section is fine – I have no comments or concerns.
- Other: Please provide explanation

The PTSD questions were easy to understand.

- Strongly disagree
- Disagree
- Neither agree nor disagree
- Agree
- Strongly agree

Please provide additional feedback on how the wording of the questions in this section could be easier to understand.

The items are relevant to Long COVID PTSD symptoms.

- Strongly disagree
- Disagree
- Neither agree nor disagree
- Agree
- Strongly agree

Please provide additional feedback on how the items in this section could be more relevant to PTSD symptoms of Long COVID.

All items in this subsection address all aspects of PTSD symptoms of Long COVID.

- Strongly disagree
- Disagree
- Neither agree nor disagree
- Agree
- Strongly agree

Please explain any additional aspects of PTSD symptoms of Long COVID not addressed in this section.

Are there any additional items you suggest we add that are missing from this section? If yes, please list them in the space provided.

Depression

The next questions focus on the **Depression** Symptoms section of the Long COVID CDE Starter Pack. As a reminder, you can use the CDE Starter Pack to refer to the items that are being asked about.

Do you want to skip this section?

- Yes
- No

You answered YES -- please indicate why you decided to skip this set of questions

- This section is not relevant to me.
- This section is fine – I have no comments or concerns.
- Other: Please provide explanation

The depression questions were easy to understand.

- Strongly disagree
- Disagree
- Neither agree nor disagree
- Agree
- Strongly agree

Please provide additional feedback on how the wording of the questions in this section could be easier to understand.

The items are relevant to Long COVID depression symptoms.

- Strongly disagree
- Disagree
- Neither agree nor disagree
- Agree
- Strongly agree

Please provide additional feedback on how the items in this section could be more relevant to depression symptoms of Long COVID.

All items in this subsection address all aspects of depression symptoms of Long COVID.

- Strongly disagree
- Disagree
- Neither agree nor disagree
- Agree
- Strongly agree

Please explain any additional aspects of depression symptoms of Long COVID not addressed in this section.

Are there any additional items you suggest we add that are missing from this section? If yes, please list them in the space provided.

Anxiety

The next questions focus on the **Anxiety** Symptoms section of the Long COVID CDE Starter Pack. As a reminder, you can use the CDE Starter Pack to refer to the items that are being asked about.

Do you want to skip this section?

- Yes

- No

You answered YES -- please indicate why you decided to skip this set of questions

- This section is not relevant to me.
- This section is fine – I have no comments or concerns.
- Other: Please provide explanation

The anxiety questions were easy to understand.

- Strongly disagree
- Disagree
- Neither agree nor disagree
- Agree
- Strongly agree

Please provide additional feedback on how the wording of the questions in this section could be easier to understand.

The items are relevant to Long COVID anxiety symptoms.

- Strongly disagree
- Disagree
- Neither agree nor disagree
- Agree

- Strongly agree

Please provide additional feedback on how the items in this section could be more relevant to anxiety symptoms of Long COVID.

All items in this subsection address all aspects of anxiety symptoms of Long COVID.

- Strongly disagree
- Disagree
- Neither agree nor disagree
- Agree
- Strongly agree

Please explain any additional aspects of anxiety symptoms of Long COVID not addressed in this section.

Are there any additional items you suggest we add that are missing from this section? If yes, please list them in the space provided.

Overall Impression

The following questions will help us understand your **overall impression** of the Long COVID CDE Starter Pack. When answering, please consider the Starter Pack as a whole.

The Long COVID CDE Starter Pack accomplished its purpose of producing long-COVID CDEs that are appropriate for the RADx-UP Community.

- Strongly disagree
- Disagree
- Neither agree nor disagree
- Agree
- Strongly agree

The Long COVID CDE Starter Pack is clearly organized.

- Strongly disagree
- Disagree
- Neither agree nor disagree
- Agree
- Strongly agree

Please provide any additional feedback related to the organization of the starter pack.

The Long COVID CDE Starter Pack is useful to capture data with the community I identify and/or work with.

- Strongly disagree
- Disagree
- Neither agree nor disagree
- Agree
- Strongly agree

Please provide any additional feedback related to how the starter pack could be more useful to capture data with the community I identify and/or work with.

The Long COVID CDE Starter Pack is comprehensive, or includes all elements that encompass Long COVID symptoms.

- Strongly disagree
- Disagree
- Neither agree nor disagree
- Agree
- Strongly agree

Please provide any additional feedback related to how the starter pack could be more comprehensive.

The Long COVID CDE Starter Pack is inclusive to a wide range of communities.

- Strongly disagree
- Disagree
- Neither agree nor disagree
- Agree
- Strongly agree

Please provide any additional feedback on how the Long COVID CDE Starter Pack could be more inclusive to a wide range of communities.

The items in the Long COVID CDE Starter Pack were easy to understand.

- Strongly disagree
- Disagree
- Neither agree nor disagree
- Agree
- Strongly agree

Please provide any additional feedback related to how the items in the starter pack could be easier to understand.

Please provide any additional feedback on how the Long COVID CDE Starter Pack could be improved.

Powered by Qualtrics
