# Supplementary material for: A community-engaged approach to developing common data elements: a case study from the RADx-UP Long COVID common data elements Task Force
Source: JAMIA Open. 2025 Jun 4;8(3):ooaf046. doi: 10.1093/jamiaopen/ooaf046 (PMC12136053; doi:10.1093/jamiaopen/ooaf046)
Supplement: ooaf046_Supplementary_Data [file ooaf046_supplementary_data.zip › Supplementary Survey 2_NovelLongCOVIDCDEs_Feedback_Survey.pdf]

Introduction

Thank you for your participation in this survey. The purpose of this survey is to get feedback on the revised Long COVID CDEs. The revised CDEs contain three sets of questions: Introductory, Symptom-based, and Quality of Life.

You are being asked to review the Long COVID CDEs and provide feedback on the content and the overall wording of the questions. We are interested in knowing if the revised set of CDEs is easy to understand, comprehensive, and inclusive to a wide range of communities. This survey is voluntary, and you are free to skip any questions you do not want to answer or stop completing the survey at any point. The survey should take approximately 20 minutes.

You can move forward and backward within the survey and edit any of your responses until you submit the survey. You are welcome to go back and review the Long COVID CDE document as you complete the survey.

If you have any questions about this activity, please contact Allyn Damman (allyn.damman@duke.edu).

User Information

These questions help us to better understand who is completing the survey. The information will also help us ensure that we obtain feedback from a diverse group of users.

First Name:

Last Name:

Email:

Long COVID Definition

We are interested in getting feedback on our definition of Long COVID. Below we have provided our definition of Long COVID. Please review the definition and comment on the language.

Long COVID is a condition that occurs when patients do not fully recover from COVID 19. Most people recover from COVID-19 within a couple of weeks, but people with Long COVID may experience new or lingering symptoms four or more weeks after being infected. Common symptoms include fatigue, difficulty breathing, brain fog, stroke, heart attack, problems controling blood sugar, and other symptoms that have an impact on everyday life. Long COVID may also be referred to as long-haul COVID, post-acute COVID-19, long-term effects of COVID or chronic COVID.

The definition accurately describes Long COVID.

- Strongly Disagree
- Disagree
- Netiher agree nor disagree
- Agree
- Strongly Agree

Please provide additional feedback on how the definition could more accurately describe Long COVID.

The language used in the definition is easy to understand.

- Strongly Disagree
- Disagree

- Neither agree nor disagree
- Agree
- Strongly Agree

Please provide additional feedback on how the language used in the definition could be easier to understand.

Introductory Questions

The next questions focus on the **Introduction** section. As a reminder, you can refer to the CDEs to review the items that are being asked about.

The Introductory questions were easy to understand.

- Strongly disagree
- Disagree
- Neither agree nor disagree
- Agree
- Strongly agree

Please provide additional feedback on how the wording of the questions in this section could be easier to understand.

Please explain any additional aspects of the Introduction questions not addressed in this section.

Are there any additional items you suggest we add that are missing from this section? If yes, please list them in the space provided.

Symptoms-based questions

The next questions focus on the **Long COVID Symptoms** questions of the Long COVID CDEs. As a reminder, you can use the CDEs to refer to the items that are being asked about

The Symptoms-based items are relevant to the most critical symptoms of Long COVID.

- Strongly disagree
- Disagree
- Neither agree nor disagree
- Agree
- Strongly agree

Please provide additional feedback on how the Symptom-based items in this section could be more relevant to the most critical symptoms of Long COVID.

The Symptoms-based items were easy to understand.

- Strongly disagree
- Disagree
- Neither agree nor disagree
- Agree
- Strongly agree

Please provide additional feedback on how the wording of the Symptom-based items could be easier to understand.

The Symptoms-based items in this section adequately described the individual symptoms.

- Strongly disagree
- Disagree
- Neither agree nor disagree
- Agree
- Strongly agree

Please provide additional feedback on how the wording of questions for specific symptoms could be improved.

The items in this section address all aspects of the most critical symptoms of Long COVID.

- Strongly disagree
- Disagree
- Neither agree nor disagree
- Agree
- Strongly agree

Please explain any additional aspects of Long COVID not addressed in this section.

Are there any additional items you suggest we add that are missing from this section? If yes, please list them in the space provided.

Quality of life questions

The next questions focus on the **Quality of Life (QOL)** questions of the Long COVID CDEs. As a reminder, you can use the CDEs to refer to the items that are being asked about.

The Quality of life questions were easy to understand.

- Strongly disagree
- Disagree
- Neither agree nor disagree
- Agree
- Strongly agree

Please provide additional feedback on how the wording of the Quality of Life questions could be easier to understand.

Please provide additional feedback on how the Quality of Life questions in this section could be more relevant.

Are there any additional items you suggest we add that are missing from this section? If yes, please list them in the space provided.

Overall Feedback

The next questions focus on Overall Feedback for the Long COVID CDEs. As a reminder, you can use the CDEs to refer to the items that are being asked about.

Considering all Sections 1-3, on a scale from 1 to 5, in your opinion, how inclusive are the Long COVID CDEs to a wide range of communities?

- 1 (Very Poor)
- 2 (Poor)
- 3 (Fair)
- 4 (Good)
- 5 (Excellent)

The Long COVID CDEs accomplished its purpose of producing Long COVID CDEs that are appropriate for the RADx-UP Community.

- Yes
- No

Powered by Qualtrics
