# Supplementary material for: A community-engaged approach to developing common data elements: a case study from the RADx-UP Long COVID common data elements Task Force
Source: JAMIA Open. 2025 Jun 4;8(3):ooaf046. doi: 10.1093/jamiaopen/ooaf046 (PMC12136053; doi:10.1093/jamiaopen/ooaf046)
Supplement: ooaf046_Supplementary_Data [file ooaf046_supplementary_data.zip › Supplementary Survey 3_NovelLongCOVIDCDEs_Feedback_Survey.pdf]

Introduction

Thank you for your time completing the **RADx-UP Long-COVID CDE Focus Group Feedback Survey**. The purpose of this survey is to assess focus group members' experience and satisfaction with the content, coordination, member composition and overall engagement while developing the Long-COVID CDEs.

You are being asked to complete this survey because you were invited to participate in the focus group as a member of the Long-COVID CDE taskforce.

Your participation is entirely **voluntary**. This means you may choose to skip any question or stop answering this survey at any time without penalty. Your responses will be a) **confidential** and b) **aggregated before reporting**. There are no expected risks to responding to this survey.

The survey will take about **10 minutes** to complete and will close on **Monday, February 20, 2023** at **5pm**.

If you have questions about how to complete this survey, please reach out to **Adriana Parker (adriana_parker@med.unc.edu)** of the CDCC Tracking and Evaluation Team.

Block 1

Please check the role(s) that **best** describe the background that you brought to the Long-COVID CDE focus group:

- ☐ RADx-UP project investigator
- ☐ RADx-UP community partner
- ☐ Long-COVID patient
- ☐ Long-COVID clinician
- ☐ RADx-UP CDCC staff
- ☐ Other operational staff not affiliated with RADx-UP
- ☐ ________ Other (please specify):

Please check all of the activities that you attended:

- ☐ Orientation - October 6
- ☐ Session 1 - October 20
- ☐ Session 2 - October 27
- ☐ Session 3 - December 8

What factors, if any, motivated you to continue participating in the sessions?

What factors, if any, kept you from participating in any session or in additional sessions?

Block 2

Please rate your level of agreement with each of the following statements about your experiences with the Long-COVID CDE focus group:

	Strongly disagree	Disagree	Neither agree nor disagree	Agree	Strongly agree
I understood the directions for reviewing	○	○	○	○	○

	Strongly disagree	Disagree	Neither agree nor disagree	Agree	Strongly agree
the starter pack of CDEs.					
I had the support I needed to review the starter pack of CDEs.					
I had enough time to review the starter pack of CDEs.					
I knew the deadline for reviewing the starter pack of CDEs.					
I felt satisfied with the amount of effort that was required for reviewing the CDEs.					
	Strongly disagree	Disagree	Neither agree nor disagree	Agree	Strongly agree
I felt comfortable bringing up any questions or concerns I might have had about the work.					
I was able to offer my expertise at the Zoom session(s).					
I felt like my opinion was valued at the Zoom sessions.					
The composition of the Long-COVID CDE focus group adequately reflected the communities impacted by Long-COVID.					

Who else should have been recruited when convening community-engaged focus groups to develop COVID-related CDEs?

Block 3

What can RADx-UP do, if anything, to engage community members in future focus groups to address other COVID-related issues?

Please check any of the future activities that you would like to participate in:

- ☐ I am interested in joining a writing team to further consider the process of developing CDEs, using a community-engaged approach.
- ☐ I am interested in reporting on the work of the Long-COVID CDE focus group to the RADx-UP consortium in a brief presentation (e.g., the Project-Wide Meeting).
- ☐ None of the above

Powered by Qualtrics
