# Supplementary material for: A community-engaged approach to developing common data elements: a case study from the RADx-UP Long COVID common data elements Task Force
Source: JAMIA Open. 2025 Jun 4;8(3):ooaf046. doi: 10.1093/jamiaopen/ooaf046 (PMC12136053; doi:10.1093/jamiaopen/ooaf046)
Supplement: ooaf046_Supplementary_Data [file ooaf046_supplementary_data.zip › Supplementary Survey Report 1_NovelLongCOVIDCDEs_FeedbackSurveyReport.pdf]

Default Report

Long COVID - Feedback

Q37 - The Introductory questions were easy to understand.

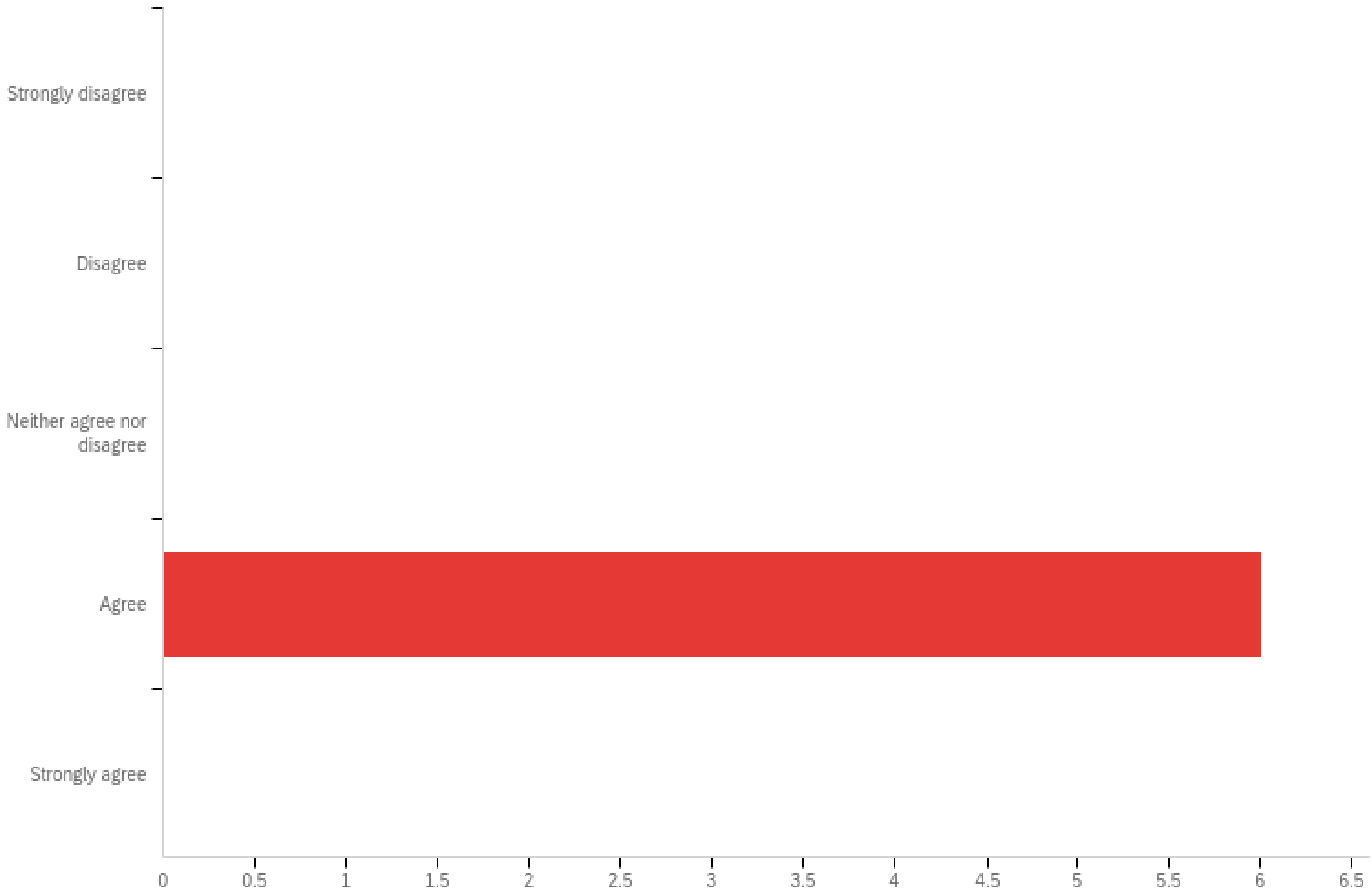

Q37 - The Introductory questions were easy to understand.

#	Field	Minimum	Maximum	Mean	Std Deviation	Variance	Count
1	The Introductory questions were easy to understand.	4.00	4.00	4.00	0.00	0.00	6

Q37 - The Introductory questions were easy to understand.

#	Answer	%	Count
1	Strongly disagree	0.00%	0
2	Disagree	0.00%	0
3	Neither agree nor disagree	0.00%	0
4	Agree	100.00%	6
5	Strongly agree	0.00%	0
	Total	100%	6

Q55 - Please provide additional feedback on how the wording of the questions in this section could be easier to understand.

Please provide additional feedback on how the wording of the questions in this section could be easier to understand.

Q57 - Please explain any additional aspects of the Introduction questions not addressed in this section.

Please explain any additional aspects of the Introduction questions not addressed in this section.
Where did you go for treatment for COVID-19? This seems out of place in this section since the section is about not being diagnosed but thinking that you might have had COVID-19.
For the question “Where did you go for treatment for COVID-19?”: Adding more options may be beneficial to include a larger range of healthcare services (ex: community health centers, walk-in clinics). Underserved areas may not have reliable access to hospital institutions and may not have a designated primary doctor and may seek help from a general practice that is nearby.
Where did you go for treatment for COVID-19?<br>(1) Hospital ICU<br>(2) Hospital in-patient<br>(3) Emergency Department<br>(4) Urgent Care<br>(5) Primary doctor<br>(6) I did not seek treatment<br>(99) Prefer not to answer"<br><br>Some people may consider "treatment" as being at home. They may isolate and just take care of themselves at home (rest, water, etc.).
"Even though you have never tested positive for COVID, was there a point in time when you think you had COVID symptoms, or believe you had COVID? " is this only for those who have selected they do not have a lab confirmed covid positive?

Q58 - Are there any additional items you suggest we add that are missing from this section? If yes, please list them in the space provided.

Are there any additional items you suggest we add that are missing from this section? If yes, please list them in the space provided.
None
Maybe can ask who are some trusted sources they trust to explain what long COVID is
for the long covid definition, can it also include Long covid does not have to be diagnoses by a PCP/healthcare professional for this study.

Q319 - The definition accurately describes Long COVID.

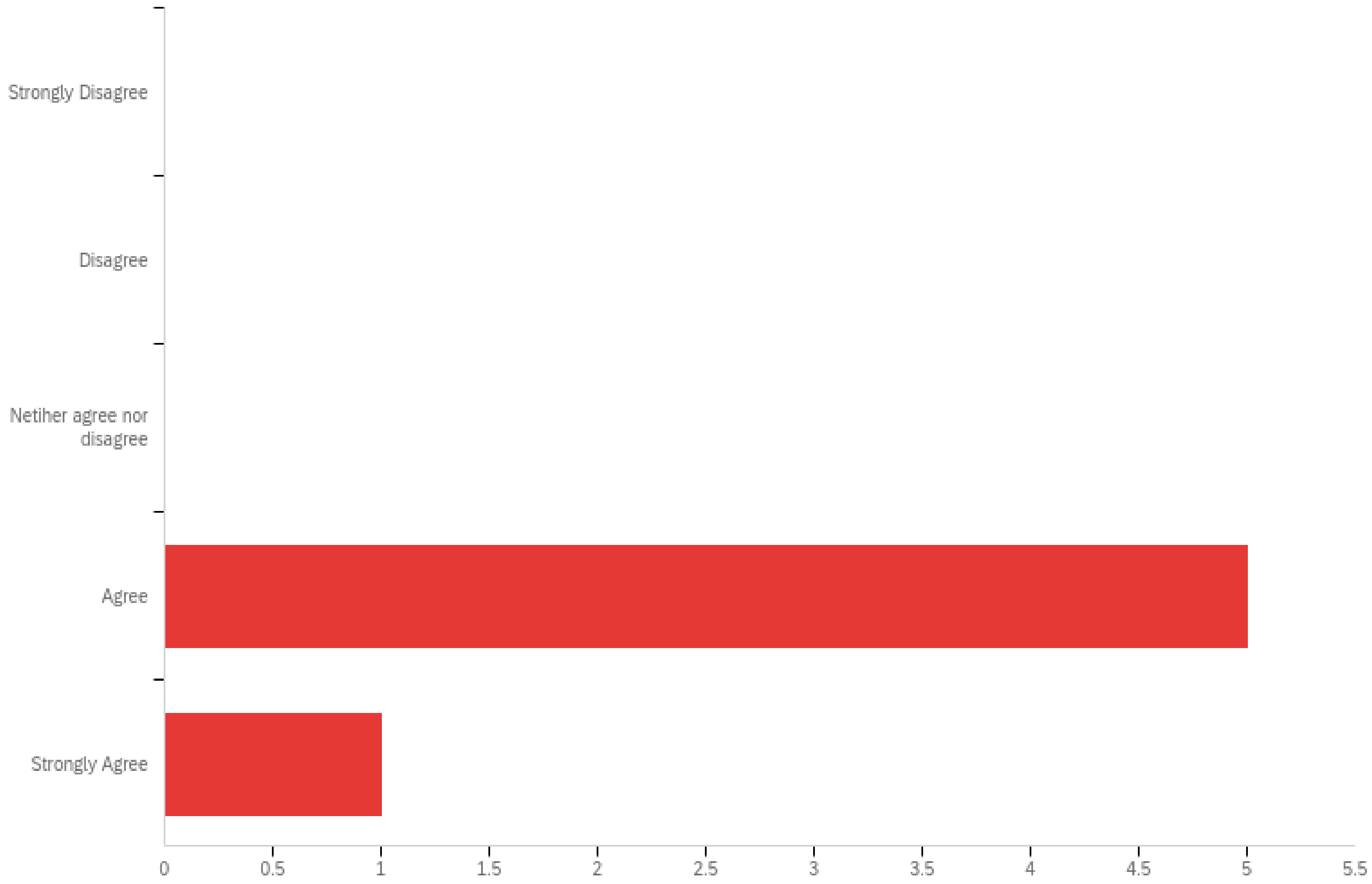

Q319 - The definition accurately describes Long COVID.

#	Field	Minimum	Maximum	Mean	Std Deviation	Variance	Count
1	The definition accurately describes Long COVID.	4.00	5.00	4.17	0.37	0.14	6

Q319 - The definition accurately describes Long COVID.

#	Answer	%	Count
1	Strongly Disagree	0.00%	0
2	Disagree	0.00%	0
3	Netiher agree nor disagree	0.00%	0
4	Agree	83.33%	5
5	Strongly Agree	16.67%	1
	Total	100%	6

Q320 - Please provide additional feedback on how the definition could more accurately describe Long COVID.

Please provide additional feedback on how the definition could more accurately describe Long COVID.

Q321 - The language used in the definition is easy to understand.

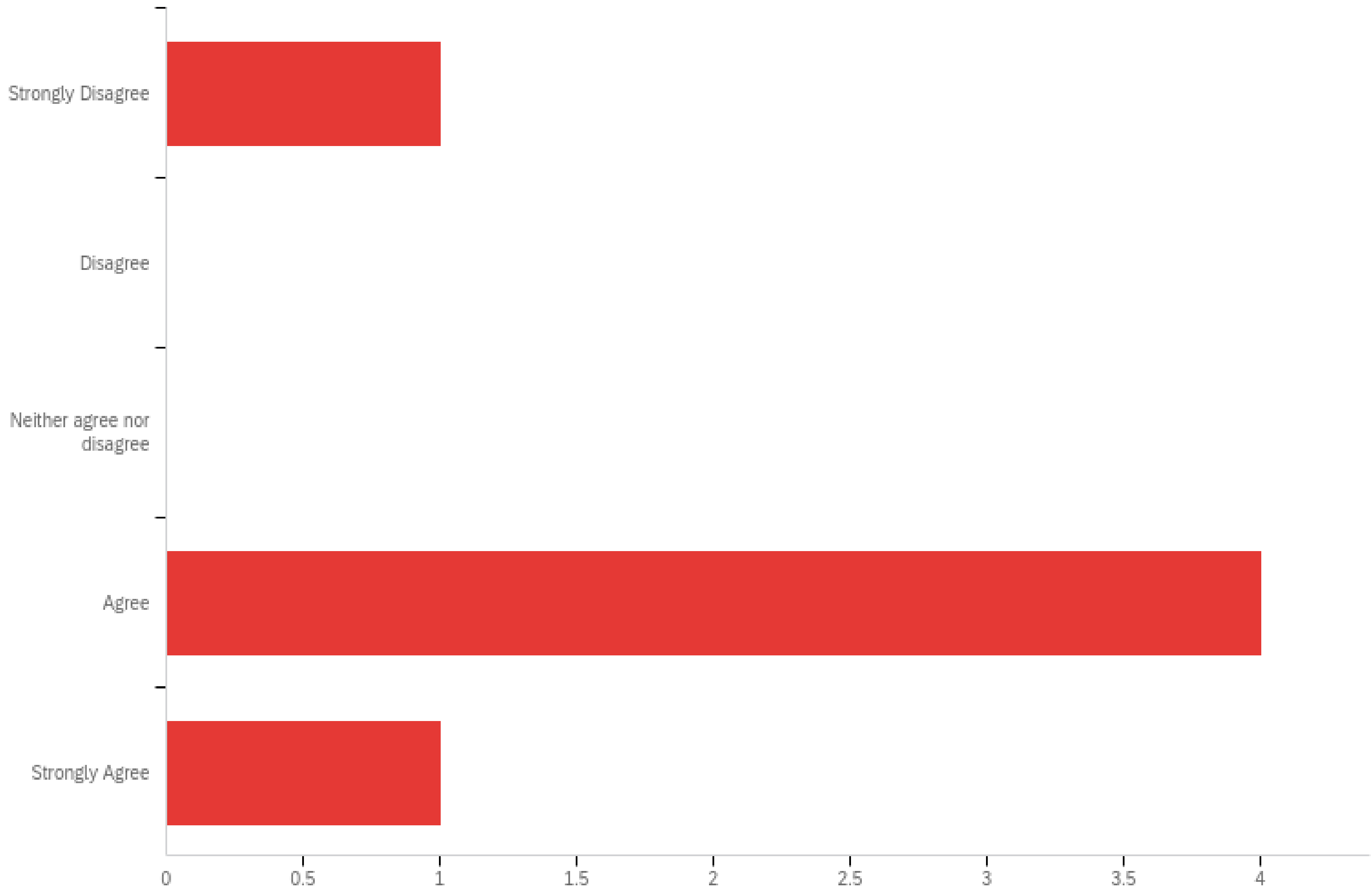

Q321 - The language used in the definition is easy to understand.

#	Field	Minimum	Maximum	Mean	Std Deviation	Variance	Count
1	The language used in the definition is easy to understand.	1.00	5.00	3.67	1.25	1.56	6

Q321 - The language used in the definition is easy to understand.

#	Answer	%	Count
1	Strongly Disagree	16.67%	1
2	Disagree	0.00%	0
3	Neither agree nor disagree	0.00%	0
4	Agree	66.67%	4
5	Strongly Agree	16.67%	1
	Total	100%	6

Q322 - Please provide additional feedback on how the language used in the definition could be easier to understand.

Please provide additional feedback on how the language used in the definition could be easier to understand.
I entered your definition into a Flesh Kincaid readability calculator and it's reading at grade 13.1 (college). Most Americans read at a 5th or 6th grade level. I think the definition is good but it should be simplified.

Q293 - The Symptoms-based items are relevant to the most critical symptoms of Long COVID.

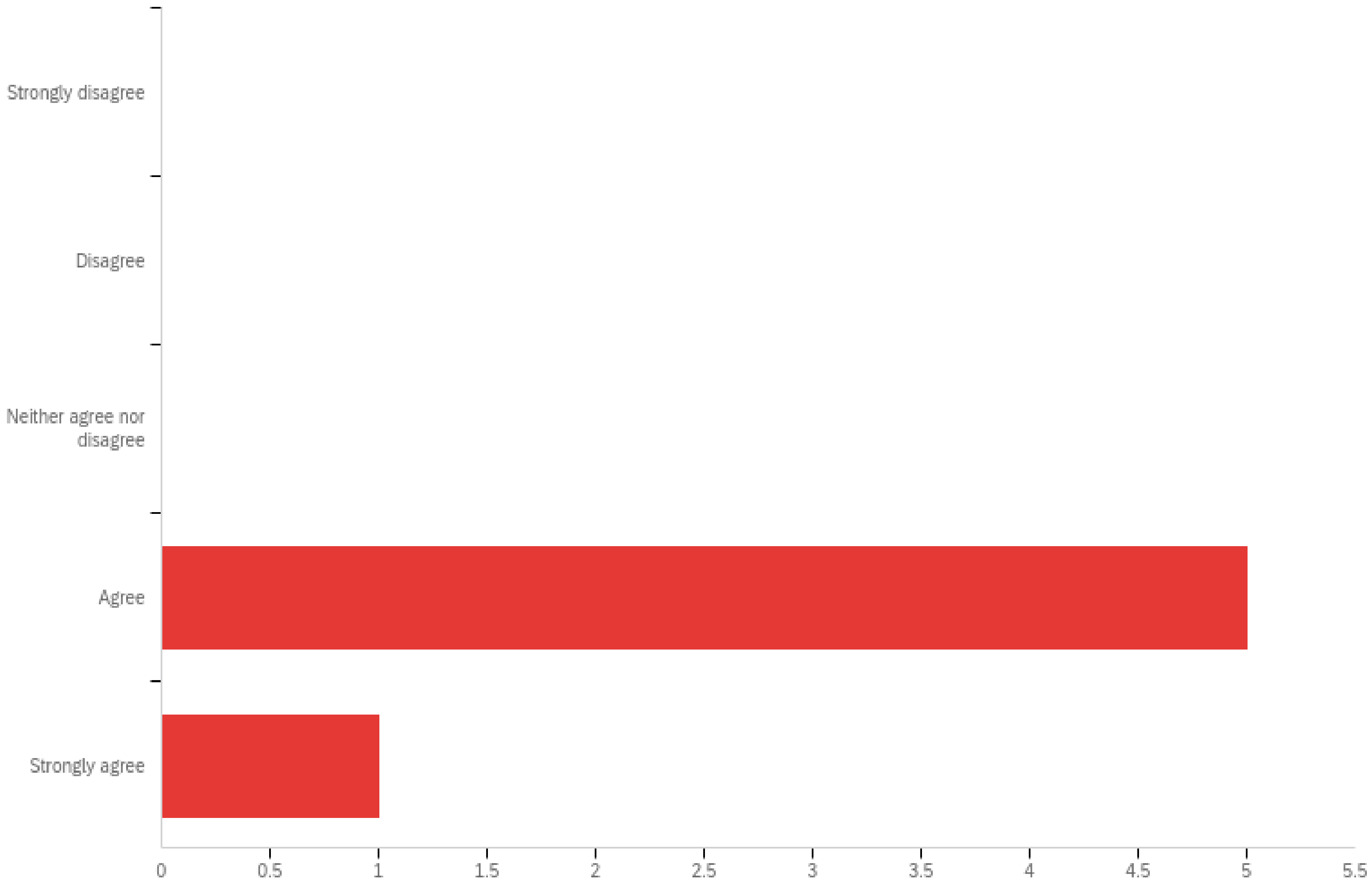

Q293 - The Symptoms-based items are relevant to the most critical symptoms of Long COVID.

#	Field	Minimum	Maximum	Mean	Std Deviation	Variance	Count
1	The Symptoms-based items are relevant to the most critical symptoms of Long COVID.	4.00	5.00	4.17	0.37	0.14	6

Q293 - The Symptoms-based items are relevant to the most critical symptoms of Long COVID.

#	Answer	%	Count
1	Strongly disagree	0.00%	0
2	Disagree	0.00%	0
3	Neither agree nor disagree	0.00%	0
4	Agree	83.33%	5
5	Strongly agree	16.67%	1
	Total	100%	6

Q316 - Please provide additional feedback on how the Symptom-based items in this section could be more relevant to the most critical symptoms of Long COVID.

Please provide additional feedback on how the Symptom-based items in this section could be more relevant to the most critical symptoms of Long COVID.

Q291 - The Symptoms-based items were easy to understand.

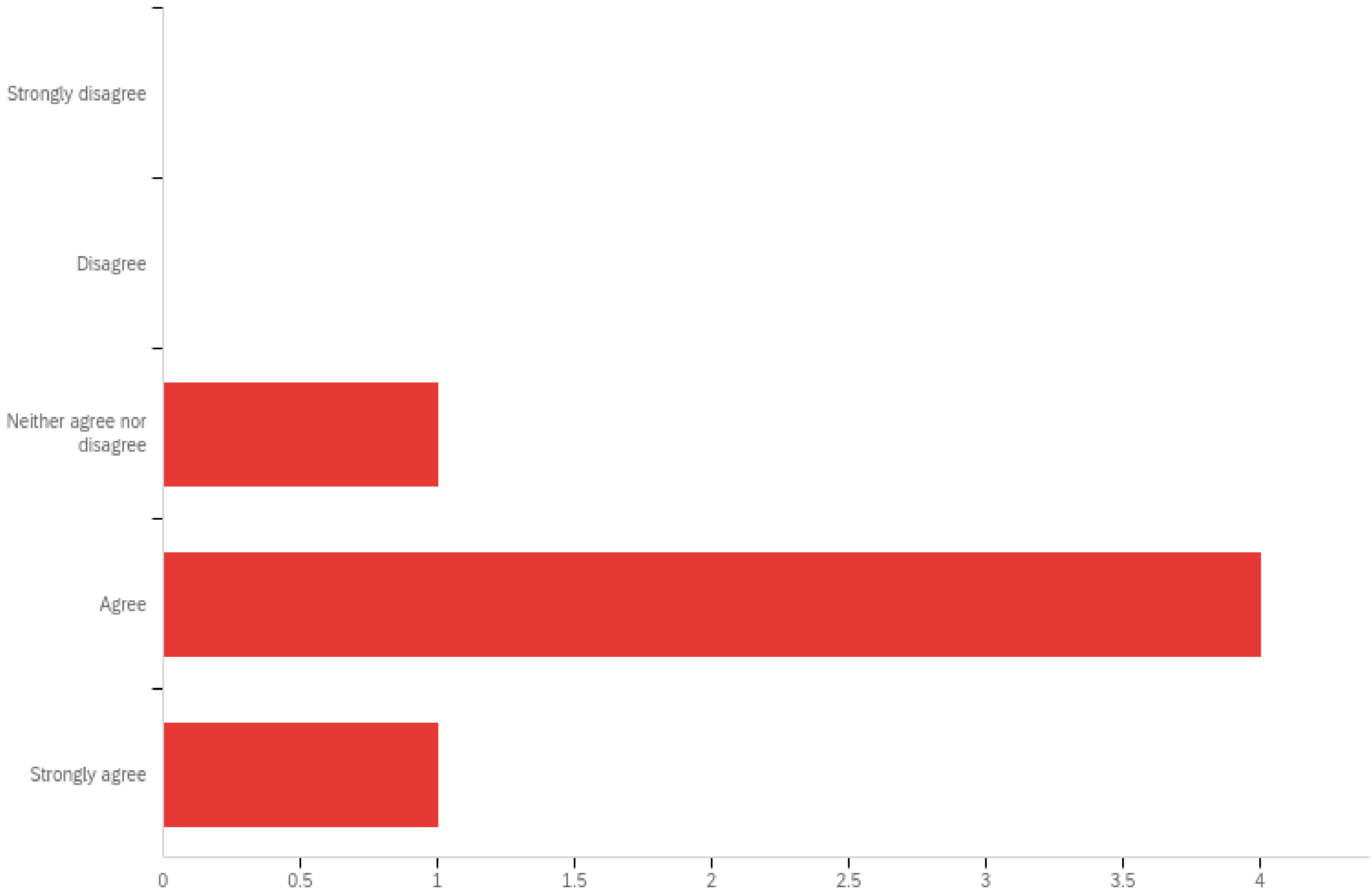

Q291 - The Symptoms-based items were easy to understand.

#	Field	Minimum	Maximum	Mean	Std Deviation	Variance	Count
1	The Symptoms-based items were easy to understand.	3.00	5.00	4.00	0.58	0.33	6

Q291 - The Symptoms-based items were easy to understand.

#	Answer	%	Count
1	Strongly disagree	0.00%	0
2	Disagree	0.00%	0
3	Neither agree nor disagree	16.67%	1
4	Agree	66.67%	4
5	Strongly agree	16.67%	1
	Total	100%	6

Q292 - Please provide additional feedback on how the wording of the Symptom-based items could be easier to understand.

Please provide additional feedback on how the wording of the Symptom-based items could be easier to understand.
Indicate time in number of weeks using the symptom you have had the longest. Not clear how we will be able to interpret this if not tied to a specific symptom.

Q306 - The Symptoms-based items in this section adequately described the individual symptoms.

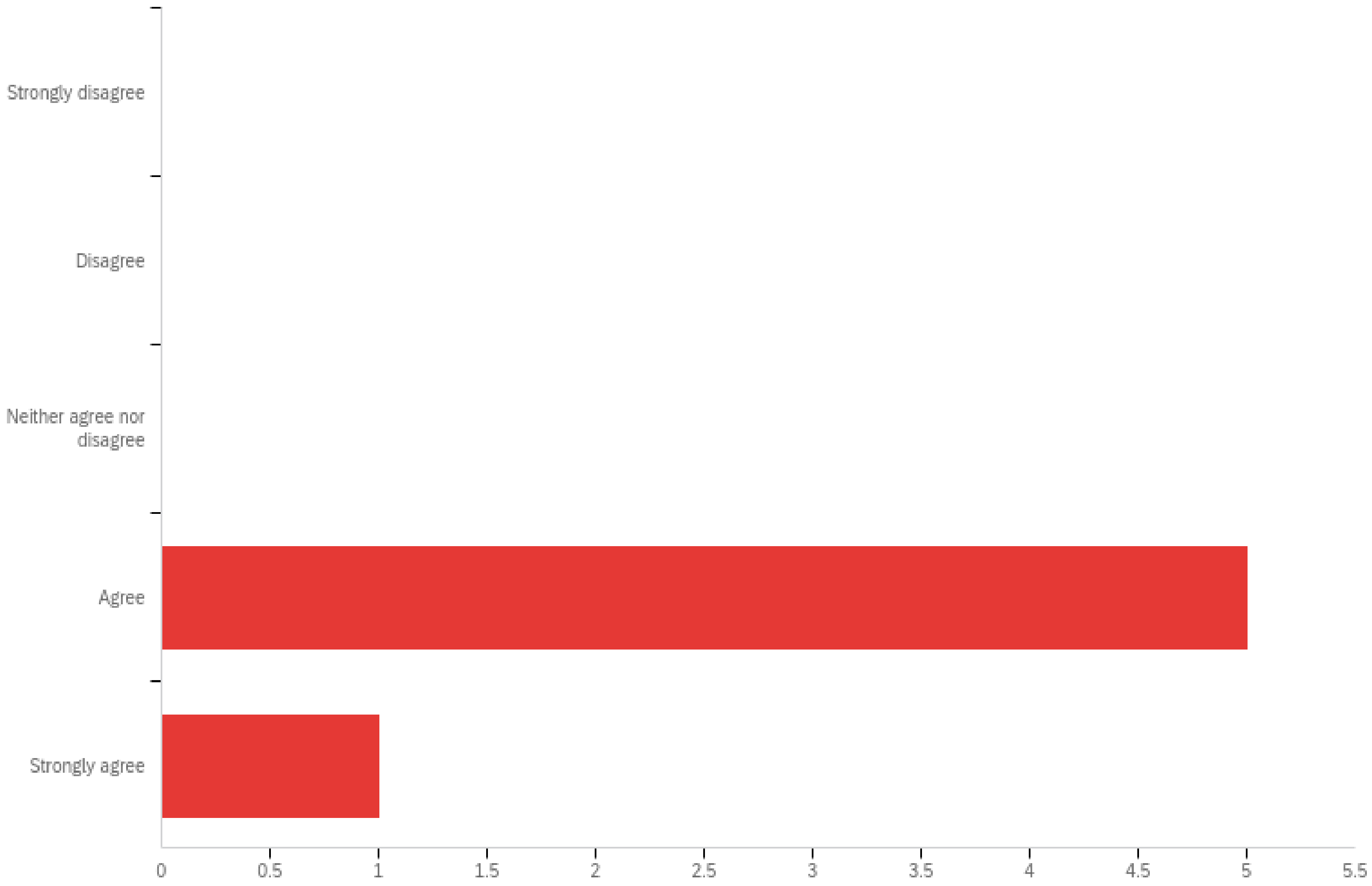

Q306 - The Symptoms-based items in this section adequately described the individual symptoms.

#	Field	Minimum	Maximum	Mean	Std Deviation	Variance	Count
1	The Symptoms-based items in this section adequately described the individual symptoms.	4.00	5.00	4.17	0.37	0.14	6

Q306 - The Symptoms-based items in this section adequately described the individual symptoms.

#	Answer	%	Count
1	Strongly disagree	0.00%	0
2	Disagree	0.00%	0
3	Neither agree nor disagree	0.00%	0
4	Agree	83.33%	5
5	Strongly agree	16.67%	1
	Total	100%	6

Q307 - Please provide additional feedback on how the wording of questions for specific symptoms could be improved.

Please provide additional feedback on how the wording of questions for specific symptoms could be improved.

Q294 - The items in this section address all aspects of the most critical symptoms of Long COVID.

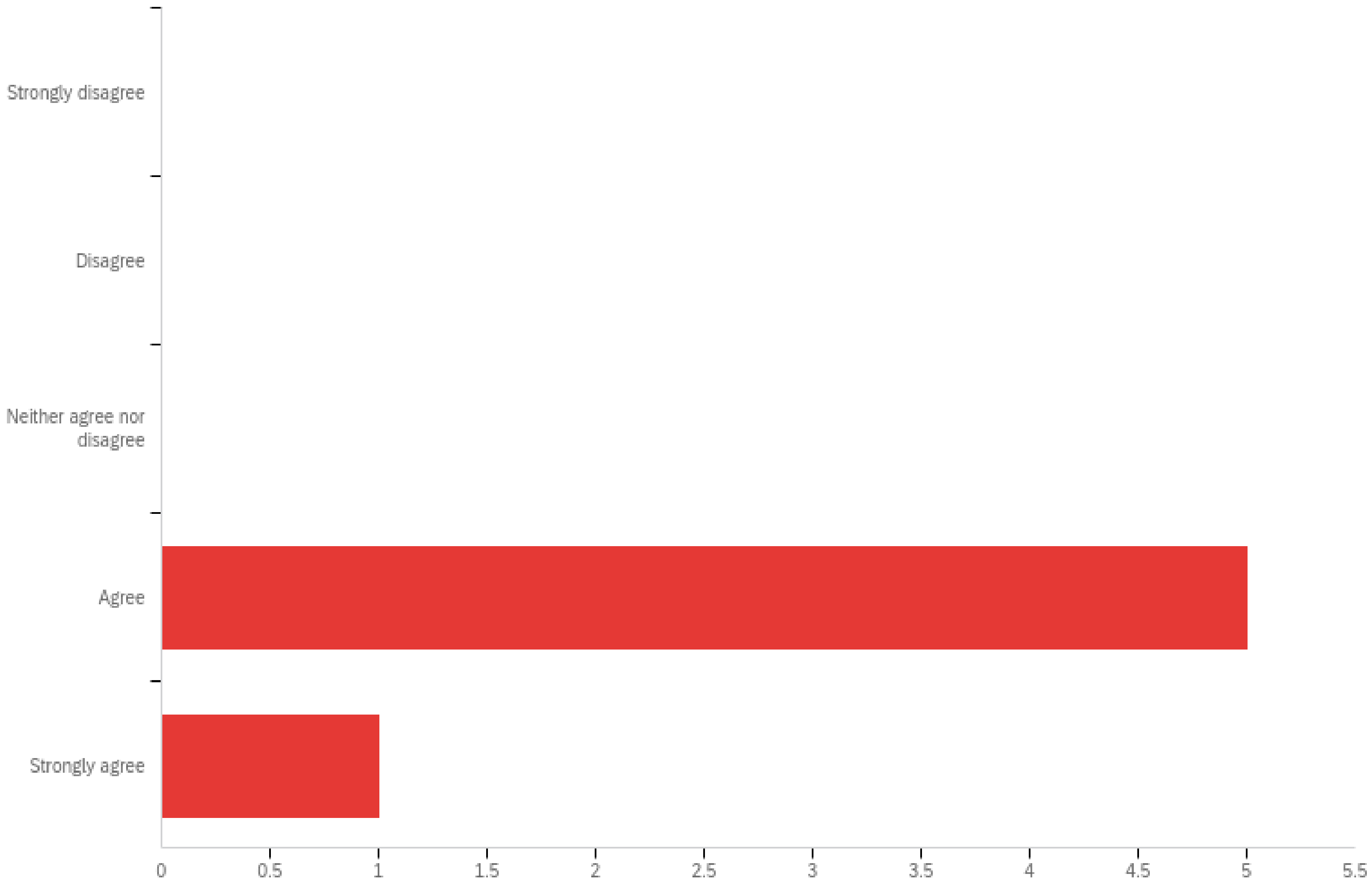

Q294 - The items in this section address all aspects of the most critical symptoms of Long COVID.

#	Field	Minimum	Maximum	Mean	Std Deviation	Variance	Count
1	The items in this section address all aspects of the most critical symptoms of Long COVID.	4.00	5.00	4.17	0.37	0.14	6

Q294 - The items in this section address all aspects of the most critical symptoms of Long COVID.

#	Answer	%	Count
1	Strongly disagree	0.00%	0
2	Disagree	0.00%	0
3	Neither agree nor disagree	0.00%	0
4	Agree	83.33%	5
5	Strongly agree	16.67%	1
	Total	100%	6

Q295 - Please explain any additional aspects of Long COVID not addressed in this section.

Please explain any additional aspects of Long COVID not addressed in this section.

Q315 - Are there any additional items you suggest we add that are missing from this section? If yes, please list them in the space provided.

Are there any additional items you suggest we add that are missing from this section? If yes, please list them in the space provided.
No
not at the moment. this section is pretty concise

Q298 - The Quality of life questions were easy to understand.

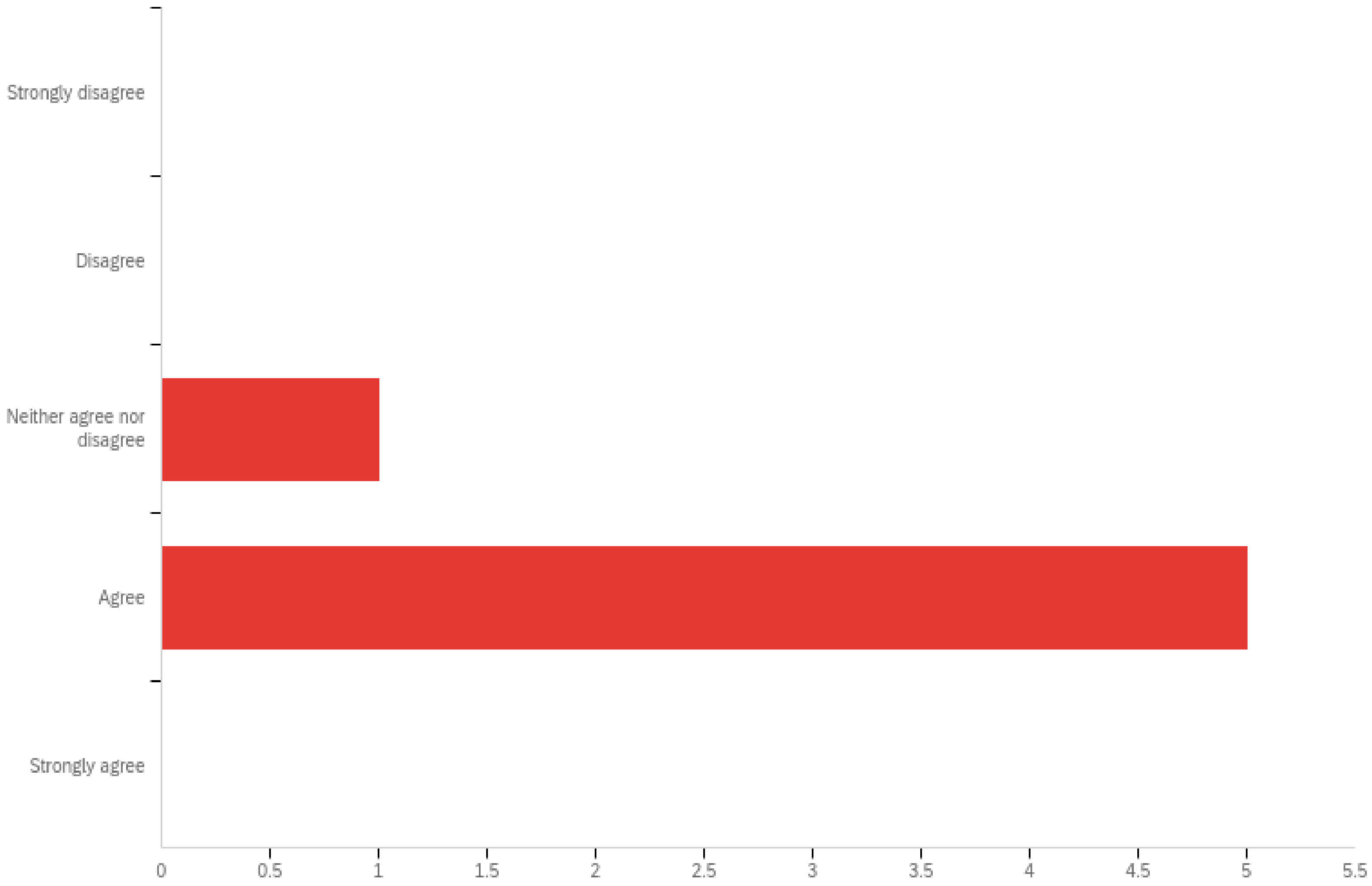

Q298 - The Quality of life questions were easy to understand.

#	Field	Minimum	Maximum	Mean	Std Deviation	Variance	Count
1	The Quality of life questions were easy to understand.	3.00	4.00	3.83	0.37	0.14	6

Q298 - The Quality of life questions were easy to understand.

#	Answer	%	Count
1	Strongly disagree	0.00%	0
2	Disagree	0.00%	0
3	Neither agree nor disagree	16.67%	1
4	Agree	83.33%	5
5	Strongly agree	0.00%	0
	Total	100%	6

Q299 - Please provide additional feedback on how the wording of the Quality of Life questions could be easier to understand.

Please provide additional feedback on how the wording of the Quality of Life questions could be easier to understand.
It is not clear if questions about family QOL and friend and neighbor QOL refers to the influence of the individual with long COVID on the QOL of other or refers to the long COVID of others.

Q301 - Please provide additional feedback on how the Quality of Life questions in this section could be more relevant.

Please provide additional feedback on how the Quality of Life questions in this section could be more relevant.
My only suggestion is maybe changing “quality of life” to “everyday life” if we’re concerned about how Long COVID has impacted their typical lives. For underserved populations, they may not resonate with the term “quality of life” if their living situations and socioeconomic status do not provide the highest quality. I think phrasing the questions as “How does Long COVID impact your every day life” may make it more understandable to make connections in how this has impacted them and their communities.
Are the QoL questions part of a skip logic? Do people who say they've never had COVID or never been diagnosed with Long COVID get these questions?<br><br>If saying "yes" to "Has a doctor diagnosed you with Long COVID?" leads to the QoL questions, then it is good.<br><br>However, if saying "no" to this question means participants don't answer the QoL questions then we'll miss out on a lot of data. For example, those most vulnerable to the effects of Long COVID may not have gone to see a doctor (lack of time, access to health care provider, etc.) and thus, would not have been diagnosed with Long COVID.
an introduction statement for this set .. such as quality of life is defined as "the standard of health, comfort, and happiness experienced by an individual or group." the next set of questions will ask about your quality of life and long covid.

Q304 - Are there any additional items you suggest we add that are missing from this section? If yes, please list them in the space provided.

**Are there any additional items you suggest we add that are missing from this section? If yes, please list them in the space provided.**
No

Q327 - Considering all Sections 1-3, on a scale from 1 to 5, in your opinion, how inclusive are the Long COVID CDEs to a wide range of communities?

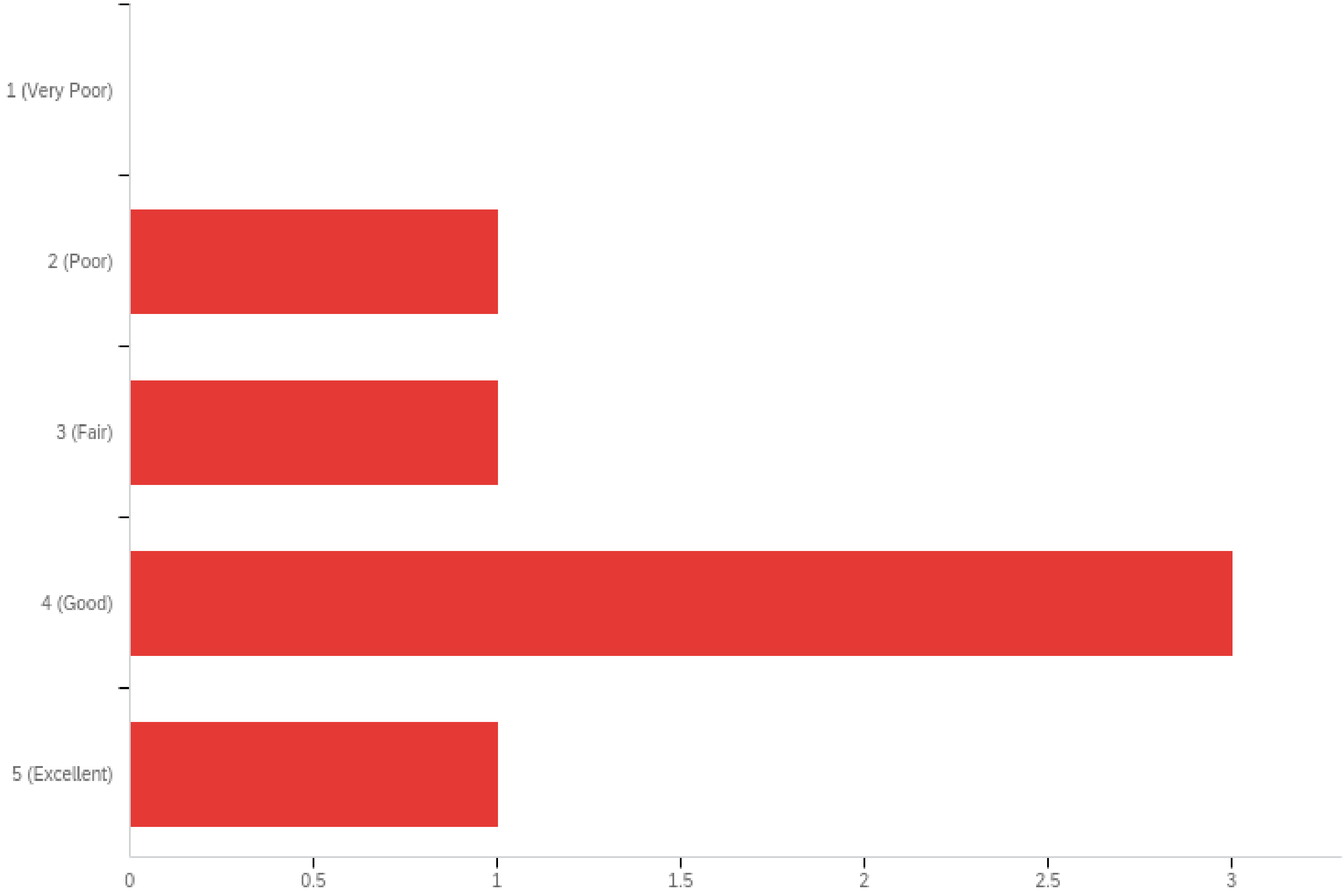

Q327 - Considering all Sections 1-3, on a scale from 1 to 5, in your opinion, how inclusive are the Long COVID CDEs to a wide range of communities?

#	Field	Minimum	Maximum	Mean	Std Deviation	Variance	Count
1	Considering all Sections 1-3, on a scale from 1 to 5, in your opinion, how inclusive are the Long COVID CDEs to a wide range of communities?	2.00	5.00	3.67	0.94	0.89	6

Q327 - Considering all Sections 1-3, on a scale from 1 to 5, in your opinion, how inclusive are the Long COVID CDEs to a wide range of communities?

#	Answer	%	Count
1	1 (Very Poor)	0.00%	0
2	2 (Poor)	16.67%	1
3	3 (Fair)	16.67%	1
4	4 (Good)	50.00%	3
5	5 (Excellent)	16.67%	1
	Total	100%	6

Q328 - The Long COVID CDEs accomplished its purpose of producing Long COVID CDEs that are appropriate for the RADx-UP Community.

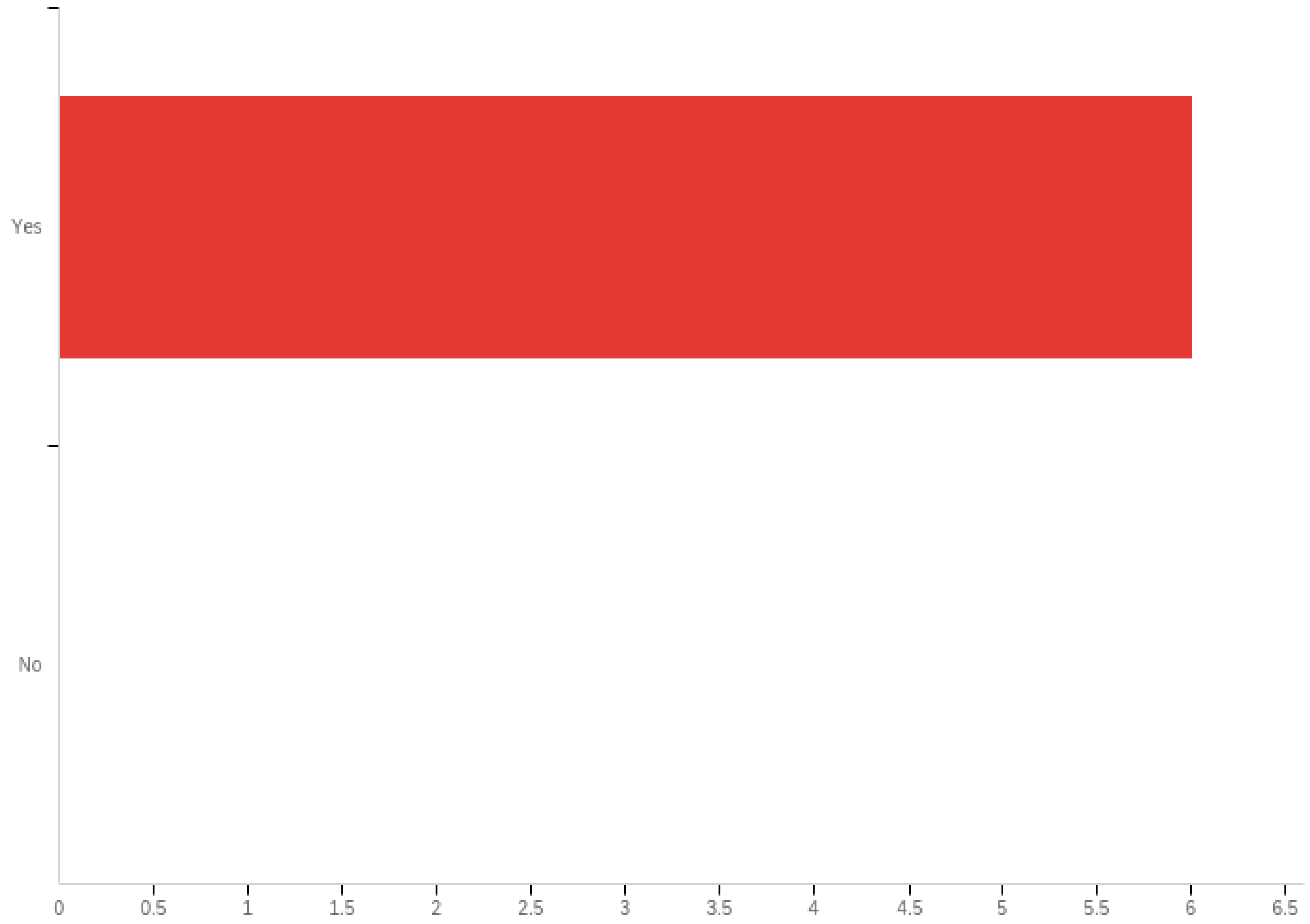

Q328 - The Long COVID CDEs accomplished its purpose of producing Long COVID CDEs that are appropriate for the RADx-UP Community.

#	Field	Minimum	Maximum	Mean	Std Deviation	Variance	Count
1	The Long COVID CDEs accomplished its purpose of producing Long COVID CDEs that are appropriate for the RADx-UP Community.	1.00	1.00	1.00	0.00	0.00	6

Q328 - The Long COVID CDEs accomplished its purpose of producing Long COVID CDEs that are appropriate for the RADx-UP Community.

#	Answer	%	Count
1	Yes	100.00%	6
2	No	0.00%	0
	Total	100%	6
