# Supplementary material for: A community-engaged approach to developing common data elements: a case study from the RADx-UP Long COVID common data elements Task Force
Source: JAMIA Open. 2025 Jun 4;8(3):ooaf046. doi: 10.1093/jamiaopen/ooaf046 (PMC12136053; doi:10.1093/jamiaopen/ooaf046)
Supplement: ooaf046_Supplementary_Data [file ooaf046_supplementary_data.zip › Supplementary Survey Report 2_LongCovidCDEs_EvalSurveyResults.pdf]

Default Report

RADx-UP Long-COVID CDE Focus Group Feedback Survey

Q1 - Please check the role(s) that best describe the background that you brought to the Long-COVID CDE focus group:

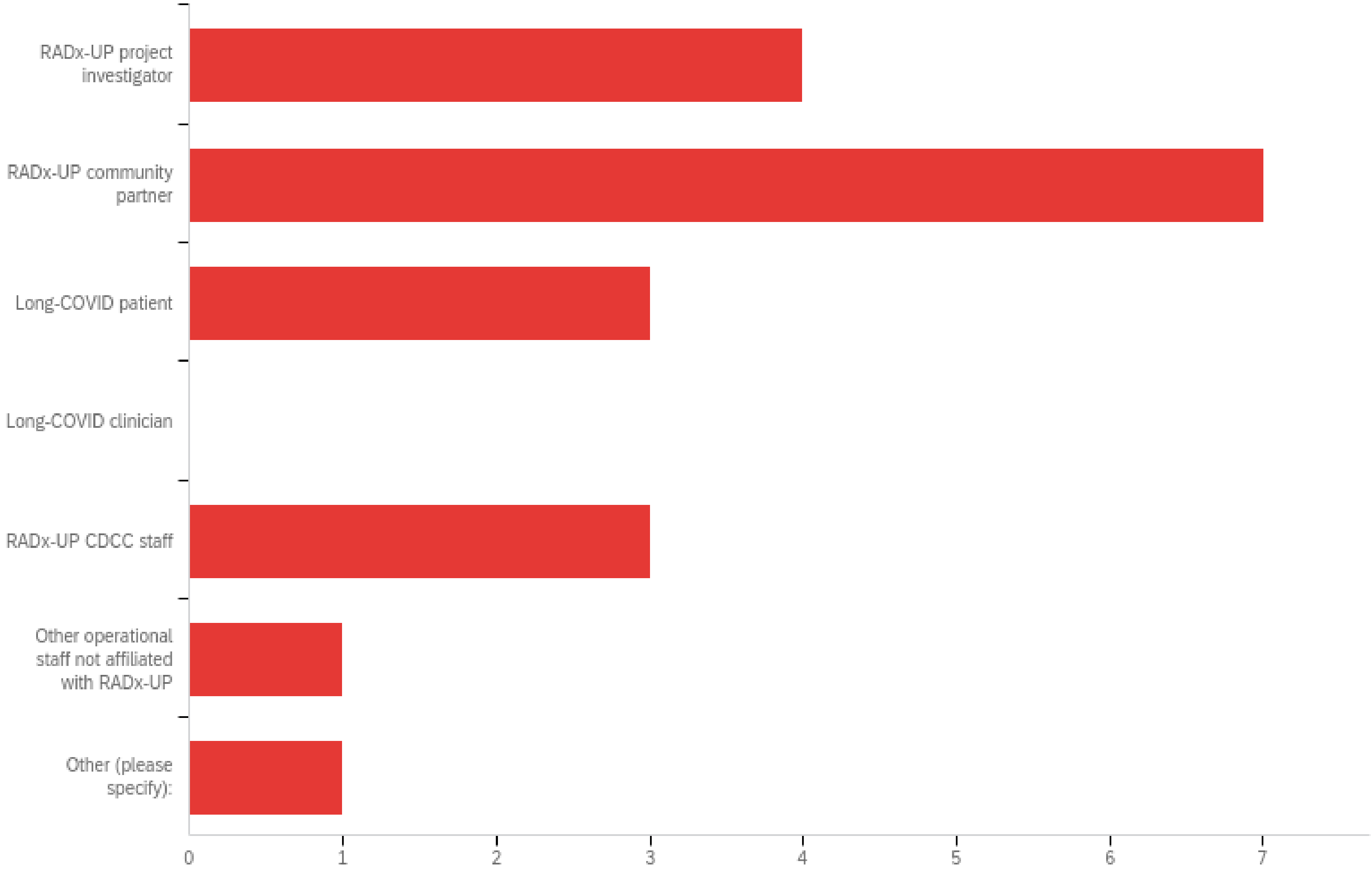

Q1 - Please check the role(s) that best describe the background that you brought to the Long-COVID CDE focus group:

#	Answer	%	Count
1	RADx-UP project investigator	21.05%	4
2	RADx-UP community partner	36.84%	7
3	Long-COVID patient	15.79%	3
4	Long-COVID clinician	0.00%	0
5	RADx-UP CDCC staff	15.79%	3
6	Other operational staff not affiliated with RADx-UP	5.26%	1
7	Other (please specify):	5.26%	1
	Total	100%	19

Q1 - Please check the role(s) that best describe the background that you brought to the Long-COVID CDE focus group:

Q1_7_TEXT - Other (please specify):

Other (please specify): - Text
RADx-UP project coordinator

Q2 - Please check all of the activities that you attended:

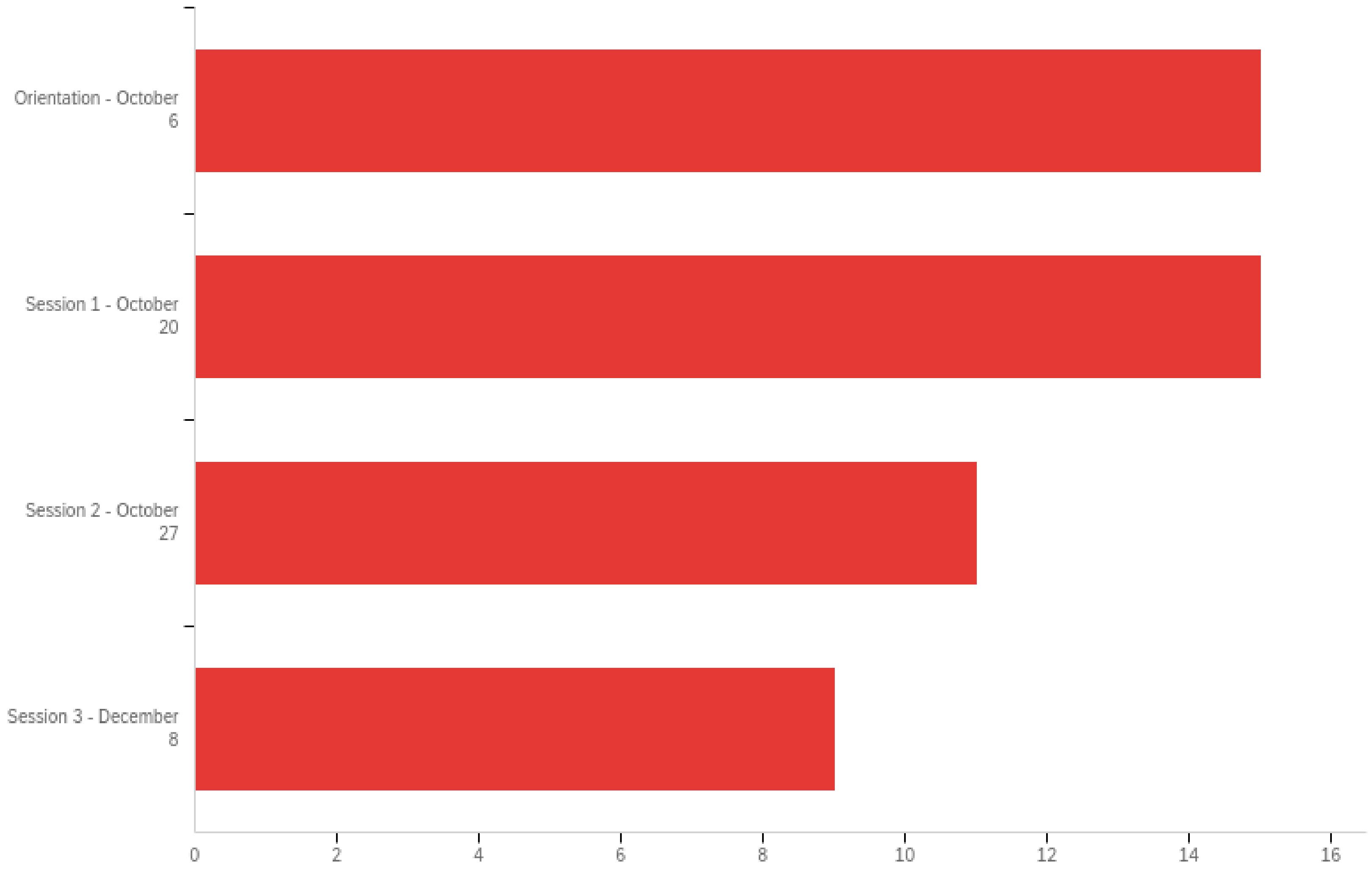

Q2 - Please check all of the activities that you attended:

#	Answer	%	Count
1	Orientation - October 6	30.00%	15
2	Session 1 - October 20	30.00%	15
3	Session 2 - October 27	22.00%	11
4	Session 3 - December 8	18.00%	9
	Total	100%	50

Q3a - What factors, if any, motivated you to continue participating in the sessions?

What factors, if any, motivated you to continue participating in the sessions?
Long COVID is an often-debilitating disease occurring in at least 10% of severe acute respiratory syndrome coronavirus 2 (SARS-CoV-2) infections. More than 200 symptoms have been identified so far, impacting multiple organ systems. Nowadays, more than 65 million individuals worldwide are estimated to have long COVID. Although research has evolved extensively in identifying several pathophysiological variations and risk factors and characterizing the disease, similarities with other viral-onset illnesses have laid the groundwork for research in the field. Even though these critical findings are crucial to understanding long COVID, current diagnostic and treatment options are scarce.
As a long COVID patient, I was motivated to have CDEs that other long COVID patients could benefit from with resources to give them possible clinics or other information to begin to get help for this condition, and so they know they are not alone. As a member of the CDCC, I think it is important that we learn more about this condition, which may further future research or give insight into long COVID.
This was an important matter to discuss how we can better serve our communities in need that were heavily impacted by COVID.
Important project, opportunity to represent the communities I live and work in
Continued support with the virtual breakout rooms

Q3a - What factors, if any, motivated you to continue participating in the sessions?

What factors, if any, motivated you to continue participating in the sessions?
Passion for the community that I serve..
Long COVID is an important topic for my study and our communities.
Understanding the prevalence of Long COVID in our communities will have lasting impact.
We were having incredibly important discussions, and I saw this project as an opportunity to assist in making sure that the CDEs were inclusive and relevant to the communities we serve.
The opportunity to contribute to the group.
Importance of the topic, my background in measurement and in CDEs
to hear from other members how to define long covid. As well as share my perspective on how to best build the research/study/survey questions

Q3b - What factors, if any, kept you from participating in any session or in additional sessions?

What factors, if any, kept you from participating in any session or in additional sessions?
Not sure what these are

Q4 - Please rate your level of agreement with each of the following statements about your experiences with the Long-COVID CDE focus group:

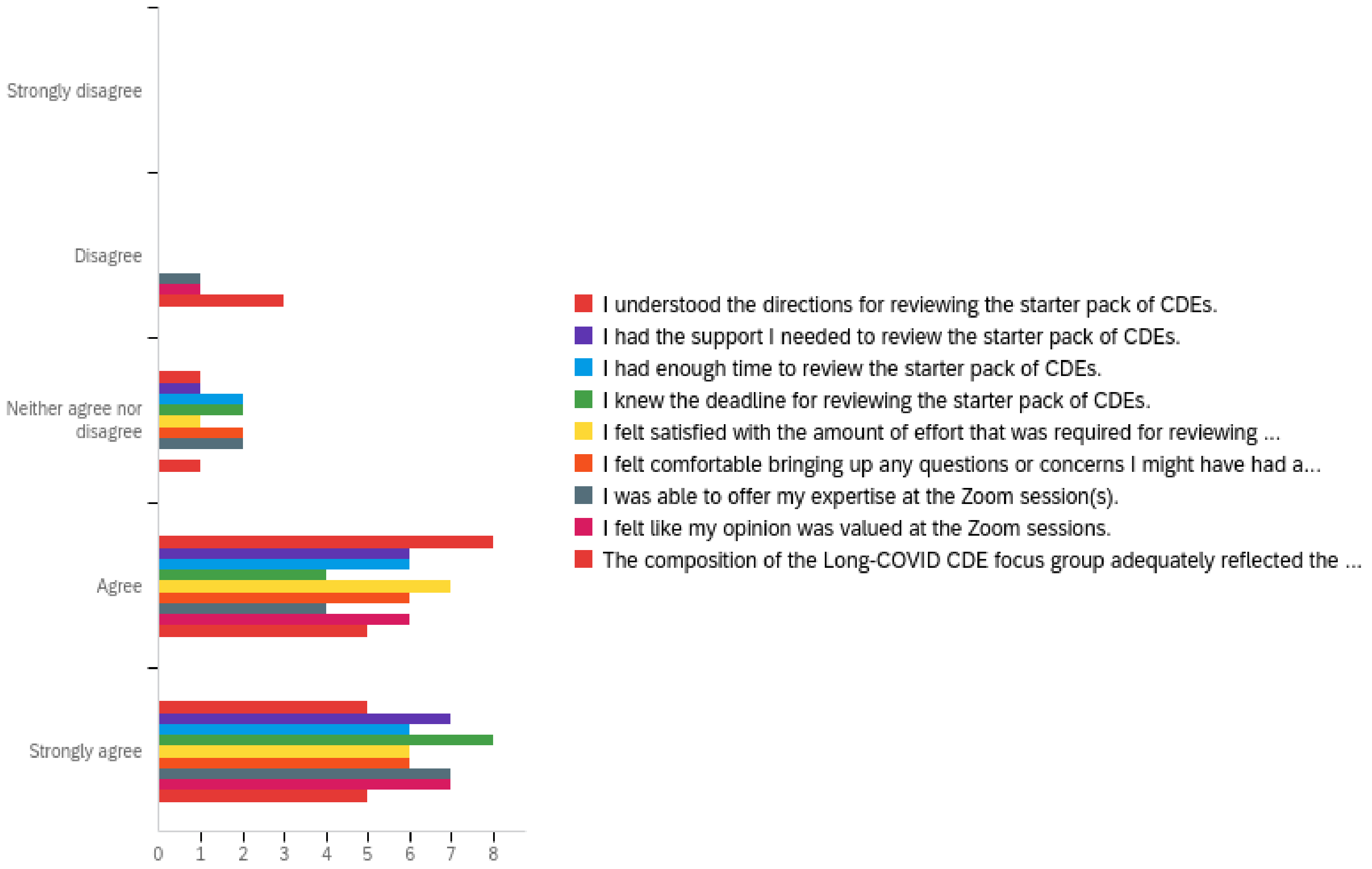

Q4 - Please rate your level of agreement with each of the following statements about your experiences with the Long-COVID CDE focus group:

#	Field	Minimum	Maximum	Mean	Std Deviation	Variance	Count
1	I understood the directions for reviewing the starter pack of CDEs.	3.00	5.00	4.29	0.59	0.35	14
2	I had the support I needed to review the starter pack of CDEs.	3.00	5.00	4.43	0.62	0.39	14
3	I had enough time to review the starter pack of CDEs.	3.00	5.00	4.29	0.70	0.49	14
4	I knew the deadline for reviewing the starter pack of CDEs.	3.00	5.00	4.43	0.73	0.53	14

Q4 - Please rate your level of agreement with each of the following statements about your experiences with the Long-COVID CDE focus group:

#	Field	Minimum	Maximum	Mean	Std Deviation	Variance	Count
5	I felt satisfied with the amount of effort that was required for reviewing the CDEs.	3.00	5.00	4.36	0.61	0.37	14
6	I felt comfortable bringing up any questions or concerns I might have had about the work.	3.00	5.00	4.29	0.70	0.49	14
7	I was able to offer my expertise at the Zoom session(s).	2.00	5.00	4.21	0.94	0.88	14

Q4 - Please rate your level of agreement with each of the following statements about your experiences with the Long-COVID CDE focus group:

#	Field	Minimum	Maximum	Mean	Std Deviation	Variance	Count
8	I felt like my opinion was valued at the Zoom sessions.	2.00	5.00	4.36	0.81	0.66	14
9	The composition of the Long-COVID CDE focus group adequately reflected the communities impacted by Long-COVID.	2.00	5.00	3.86	1.12	1.27	14

Q4 - Please rate your level of agreement with each of the following statements about your experiences with the Long-COVID CDE focus group:

#	Question	Strongly disagree		Disagree		Neither agree nor disagree		Agree		Strongly agree		Total
1	I understood the directions for reviewing the starter pack of CDEs.	0.00%	0	0.00%	0	7.14%	1	57.14%	8	35.71%	5	14
2	I had the support I needed to review the starter pack of CDEs.	0.00%	0	0.00%	0	7.14%	1	42.86%	6	50.00%	7	14
3	I had enough time to review the starter pack of CDEs.	0.00%	0	0.00%	0	14.29%	2	42.86%	6	42.86%	6	14
4	I knew the deadline for reviewing the starter pack of CDEs.	0.00%	0	0.00%	0	14.29%	2	28.57%	4	57.14%	8	14

Q4 - Please rate your level of agreement with each of the following statements about your experiences with the Long-COVID CDE focus group:

#	Question	Strongly disagree		Disagree		Neither agree nor disagree		Agree		Strongly agree		Total
5	I felt satisfied with the amount of effort that was required for reviewing the CDEs.	0.00%	0	0.00%	0	7.14%	1	50.00%	7	42.86%	6	14
6	I felt comfortable bringing up any questions or concerns I might have had about the work.	0.00%	0	0.00%	0	14.29%	2	42.86%	6	42.86%	6	14
7	I was able to offer my expertise at the Zoom session(s).	0.00%	0	7.14%	1	14.29%	2	28.57%	4	50.00%	7	14

Q4 - Please rate your level of agreement with each of the following statements about your experiences with the Long-COVID CDE focus group:

#	Question	Strongly disagree		Disagree		Neither agree nor disagree		Agree		Strongly agree		Total
8	I felt like my opinion was valued at the Zoom sessions.	0.00%	0	7.14%	1	0.00%	0	42.86%	6	50.00%	7	14
9	The composition of the Long-COVID CDE focus group adequately reflected the communities impacted by Long-COVID.	0.00%	0	21.43%	3	7.14%	1	35.71%	5	35.71%	5	14

Q4b - Who else should have been recruited when convening community-engaged focus groups to develop COVID-related CDEs?

Who else should have been recruited when convening community-engaged focus groups to develop COVID-related CDEs?
I wasn't able to determine if other community members were invited. There wasn't a way to identify
I do not think that there were any members from community-based organizations present.
More community partners and medical physicians.
individuals with long COVID

Q5 - What can RADx-UP do, if anything, to engage community members in future focus groups to address other COVID-related issues?

What can RADx-UP do, if anything, to engage community members in future focus groups to address other COVID-related issues?
These CDEs are very important. As the COVID pandemic evolves, findings through these CDEs will be critical to understanding long COVID from a community-engaged approach.
I thoroughly enjoyed being a part of this process. It really was a quick process, obviously not as quick as picking CDEs and rolling them out, but to have a truly collaborative, community-involved processed, this process should be used in the future to address other COVID-related issues. I thought the community members and experts had fantastic feedback and it was valuable to creating a meaningful set of CDEs.
Possibly include key community leaders to be apart of these sessions in identified areas and/or representative of certain minority/indigenous groups.
Provide simple, inexpensive incentives, such as prizes
none
If you've invited some representatives from community members, ask them each to identify one other person in their circles of influence that represent the community in a manner other than what they represent the community themselves.
Invite more community members to join and have sessions only for them.

Q5 - What can RADx-UP do, if anything, to engage community members in future focus groups to address other COVID-related issues?

What can RADx-UP do, if anything, to engage community members in future focus groups to address other COVID-related issues?
An invitation should be extended via the RADx investigators.
more outreach with community partners.
Referral from projects
Further outreach to bring in further community members to give raw, unedited feedback not scholar produced feedback.
